# Chemistry of polyhalogenated nitrobutadienes, 17: Efficient synthesis of persubstituted chloroquinolinyl-1*H*-pyrazoles and evaluation of their antimalarial, anti-SARS-CoV-2, antibacterial, and cytotoxic activities

**DOI:** 10.3762/bjoc.18.54

**Published:** 2022-05-09

**Authors:** Viktor A Zapol’skii, Isabell Berneburg, Ursula Bilitewski, Melissa Dillenberger, Katja Becker, Stefan Jungwirth, Aditya Shekhar, Bastian Krueger, Dieter E Kaufmann

**Affiliations:** 1 Institute of Organic Chemistry, Clausthal University of Technology, Leibnizstr. 6, 38678 Clausthal-Zellerfeld, Germany; 2 Biochemistry and Molecular Biology Interdisciplinary Research Center, Justus Liebig University Giessen, Heinrich-Buff-Ring 26-32, 35392 Giessen, Germany; 3 Helmholtz Centre for Infection Research (HZI), Inhoffenstr. 7, 38124 Braunschweig, Germany

**Keywords:** antimalarial activity, anti-SARS-CoV-2 activity, chloroquine, 2-nitroperchlorobutadiene, nucleophilic vinylic substitution, 1*H*-pyrazoles

## Abstract

A series of 26 novel 1-(7-chloroquinolin-4-yl)-4-nitro-1*H*-pyrazoles bearing a dichloromethyl and an amino or thio moiety at C3 and C5 has been prepared in yields up to 72% from the reaction of 1,1-bisazolyl-, 1-azolyl-1-amino-, and 1-thioperchloro-2-nitrobuta-1,3-dienes with 7-chloro-4-hydrazinylquinoline. A new way for the formation of a pyrazole cycle from 3-methyl-2-(2,3,3-trichloro-1-nitroallylidene)oxazolidine (**6**) is also described. In addition, the antimalarial activity of the synthesized compounds has been evaluated in vitro against the protozoan malaria parasite *Plasmodium falciparum*. Notably, the 7-chloro-4-(5-(dichloromethyl)-4-nitro-3-(1*H*-1,2,4-triazol-1-yl)-1*H*-pyrazol-1-yl)quinoline (**3b**) and 7-chloro-4-(3-((4-chlorophenyl)thio)-5-(dichloromethyl)-4-nitro-1*H*-pyrazol-1-yl)quinoline (**9e**) inhibited the growth of the chloroquine-sensitive *Plasmodium falciparum* strain 3D7 with EC_50_ values of 0.2 ± 0.1 µM (85 ng/mL, 200 nM) and 0.2 ± 0.04 µM (100 ng/mL, 200 nM), respectively. Two compounds (**3b** and **10d**) have also been tested for anti-SARS-CoV-2, antibacterial, and cytotoxic activity.

## Introduction

**Figure 1:**
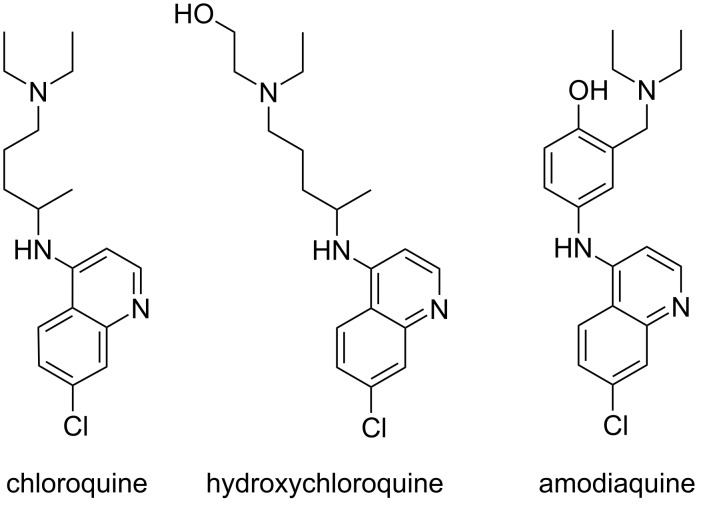
The structures of chloroquine, hydroxychloroquine, and amodiaquine.

Tropical malaria remains one of the most devastating human diseases with over half of the world’s population being at risk of infection. In 2019, there were 229 million cases of malaria worldwide resulting in an estimated 409,000 fatalities [1]. Chloroquine has been utilized extensively for decades because of its efficacy, safety, and low cost. However, the widespread resistance of *Plasmodium falciparum* to chloroquine has hampered efforts to combat malaria [2] resulting in artemisinin-based combination therapies as currently recommended standard. Notably, the use of chloroquine, hydroxychloroquine, and amodiaquine (Figure 1) against a SARS-CoV-2 infection is currently under discussion [3–7].

As the 4-amino-substituted 7-chloroquinoline is the essential fragment of chloroquine and its derivatives, we have designed and synthesized a series of pyrazoles containing this fragment in position 1 of the pyrazole ring with the aim to obtain new compounds with antimalarial and/or anti-SARS-CoV activity. Polyhalo-1,3-butadienes, carrying at least one nitro group, are valuable starting materials for the directed synthesis of highly functionalized heterocycles. During the past years, we have reported on the syntheses of a wide range of diverse substance classes applying this useful starting material. In the course of our studies concerning polyhalogenated nitrodienes, in many cases 2-nitroperchlorobuta-1,3-diene (**1**) [8–10] or nitrotrichloroethylene [11–13] have proved as appropriate precursors for a diverse variety of synthetically and/or physiologically interesting chemical compounds. Recently, we have developed an efficient method for the synthesis of persubstituted nitropyrazoles from diene **1** [14].

In this paper we are describing the formation of uniquely persubstituted 1*H*-pyrazoles with four different substituents: in position 1 of the pyrazol cycle a 7-chloroquinolin-4-yl unit, in position 3 various amino or thio fragments, in position 4 a nitro and in position 5 a dichloromethyl group. Some biological activities of 7-chloroquinolinyl-substituted pyrazole derivatives have already been described in the literature [15–18]. The antimalarial activity of chloroquinolinyl-pyrazoles, synthesized from the reaction of 1,1,1-trifluoro-4-methoxy-3-alken-2-ones with 4-hydrazinyl-7-chloroquinoline, has been evaluated in vitro against a chloroquine-resistant *Plasmodium falciparum* strain [19]. Certain 7-chloroquinolinyl-pyrazole derivatives have also shown antibacterial [20], hypoglycemic as well as antioxidant activity [21] or can be used as selective nonpeptide neurotensin receptor type 2 compounds [22]. Some azolylquinolines have been used as agrochemical fungicides, such as 7-chloro-4-(4-trimethylsilylethynyl-1-pyrazolyl)quinoline at 100 ppm which gave a 100% curative effect in barley infected with barley powdery mildew [23]. A statistical model to predict the structural requirement of 4-(5-trifluoromethyl-1*H*-pyrazol-1-yl)chloroquine derivatives to inhibit *Plasmodium* has been developed and is reported in the literature [24].

## Results and Discussion

**Scheme 1:**
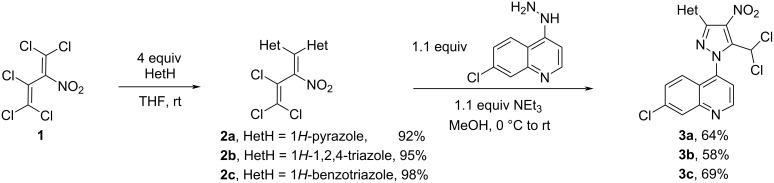
Synthesis of 3-azolylpyrazoles **3a**–**c**.

The vinylic S_N_ reaction of 2-nitroperchlorobutadiene (**1**) with four equivalents of the azoles such as 1*H*-pyrazole, 1*H*-1,2,4-triazole, or 1*H*-benzotriazole affords similarly to [25] the corresponding 1,1-bisazolylbutadienes **2a**–**c** with up to 98% yield (Scheme 1). The regiospecificity is caused by the fact that the LUMO of diene **1** is located preferentially at the dichloronitrovinyl fragment, and to an extent of 67% (by using MINDO/3 as semi-empirical method for the quantum calculation of the molecular electronic structure) to 85% (by using MNDO) at the C1 carbon atom [8]. The treatment of the azolylbutadienes **2** with 7-chloro-4-hydrazinylquinoline in methanol at room temperature using triethylamine as a base leads to the formation of the 7-chloro-4-(5-(dichloromethyl)-3-azolyl-4-nitro-1*H*-pyrazol-1-yl)quinolines **3a**–**c** in moderate yields (58–69%).

**Scheme 2:**
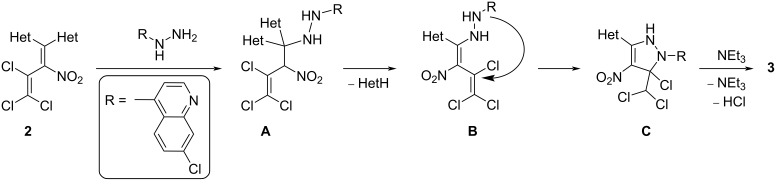
Assumed mechanism for the formation of 1*H*-pyrazoles **3a**–**c**.

A conceivable mechanistic pathway for the reaction cascade to pyrazoles **3** is shown in Scheme 2. Initially, a first molecule of the strong nucleophile 7-chloro-4-hydrazinylquinoline is assumed to react with the nitrodiazolylvinyl subunit of **2** to give butene **A**. 1,2-Elimination of an azole from **A** leads to formation of an isolable diene **B**. Upon further heating, the amino group attacks the electrophilic C–Cl position of the trichlorovinylic group intramolecularly, leading to a 2,3-dihydro-1*H*-pyrazole **C**. Finally, pyrazoles **3** are obtained upon 1,3-elimination of hydrochloric acid.

**Scheme 3:**
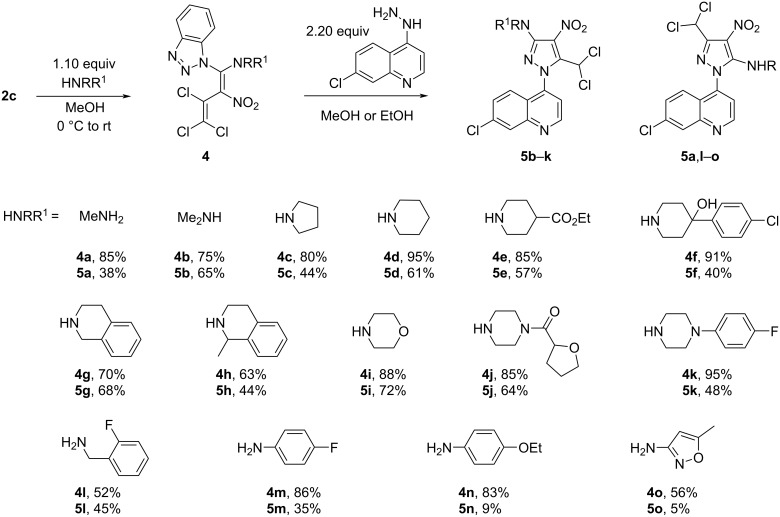
Synthesis of 3-aminopyrazoles **5b**–**k** and 5-aminopyrazoles **5a** and **5l**–**o**.

The next step of our investigation was the two-step synthesis of persubstituted aminopyrazoles **5** upon reaction of the benzotriazolyl derivative **2c** with primary, secondary, aliphatic, or (het)aromatic amines (first step) and successive reaction of the obtained dienes **4** with 7-chloro-4-hydrazinylquinoline. Thus, the reaction of compound **2c** with methylamine, 1,2,3,4-tetrahydroisoquinoline, 1-methyl-1,2,3,4-tetrahydroisoquinoline, (2-fluorophenyl)methylamine, and 5-methylisoxazolyl-3-amine proceeded smoothly under mild conditions (methanol, 0 °C to rt), similarly to the previously obtained dienes **4b**–**e** [14], **4f** [9], **4i** [14], **4j** [9], **4k** [26], and **4m**,**n** [27], and led to dienes **4a**, **4g**, **4h**, **4l**, and **4o**, respectively, in yields of 52–85%. The subsequent treatment of the butadienes **4** with 2.20 equivalents of 7-chloro-4-hydrazinylquinoline in methanol or ethanol led to the formation of pyrazoles **5** in strongly varying yields (5–72%) and dual orientation (either 3,5- or 5,3-positions) of an amino and dichloromethyl unit in the pyrazole (Scheme 3).

**Scheme 4:**
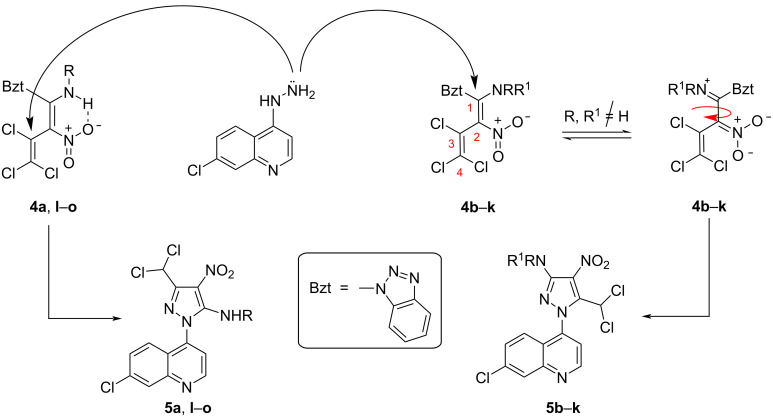
Orientation of nucleophilic attack of 7-chloro-4-hydrazinylquinoline on nitrobutadienes **4**.

We suggest that dienes **4a**,**l**–**o**, obtained from **2c** and primary amines are stabilized by formation of intramolecular hydrogen bonds between the NH and the NO_2_ group forming a six-membered ring system that upon reaction with 7-chloro-4-hydrazinylquinoline formed 5-aminopyrazoles **5a**,**l**–**o**. Here, a nucleophilic attack of the NH_2_ group of the arylhydrazine on the C3 position of the butadiene chain is observed due to the sterically hindered rigidized six-membered ring system in the dienes **4a**,**l**–**o** (Scheme 4). The obtained 5-aminopyrazoles **5a**,**l**–**o** show the following ^13^C NMR shifts of the pyrazole ring and the dichloromethyl group: 149.5–149.9 ppm (C-NHR), 114.7–116.1 ppm (C-NO_2_), 147.0–147.2 ppm (C=N), and 62.2–62.3 ppm (CHCl_2_). On the other hand, the dienes **4b**–**k**, obtained from reaction of **2c** and secondary amines, are obviously stabilized by the effective conjugation between the amino and nitro groups and react with 7-chloro-4-hydrazinylquinoline to give 3-aminopyrazoles **5b**–**k**. In this case, the nucleophilic attack of the NH_2_ group of ArNHNH_2_ on the C1 position of the butadiene chain is more likely due to the possibility of a rotation around the C1–C2 bond in **4b**–**k**. The 3-aminopyrazoles **5b**–**k** show considerable differences of the ^13^C shifts of C-NO_2_-, *C*-CHCl_2_- and CHCl_2_-groups in comparison with the corresponding ^13^C shifts of 5-aminopyrazoles **5a**,**l–o**. The ^13^C NMR shifts of the pyrazole ring and the dichloromethyl group of 3-aminopyrazoles **5b**–**k** are 151.1–154.0 ppm (C-NRR^1^), 120.6–122.2 ppm (C-NO_2_), 136.7–136.9 ppm (*C*-CHCl_2_), and 57.5–57.7 ppm (CHCl_2_).

The obtained ^13^C NMR data of pyrazoles **5** match the calculated NMR shifts (Table S1 in Supporting Information File 1). The predicted and found data for compounds **5a** and **5b** correspond well and thus support our assumptions regarding the orientation of the nucleophilic attack of 7-chloro-4-hydrazinylquinoline to nitrodienes **4**.

**Scheme 5:**
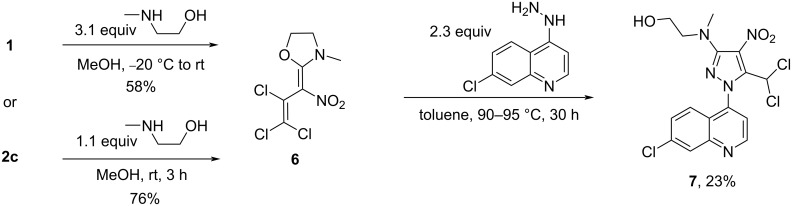
Synthesis of oxazolidine **6** and pyrazole **7**.

Further, we developed a new way for the formation of a pyrazole cycle from oxazolidine **6** and a hetarylhydrazine. The oxazolidine **6** was synthetized under mild reaction conditions either from nitrodiene **1** (yield 58%) or from the benzotriazolyl derivative **2c** (yield 76%). The increase in yield in case of **2c** supports the suggestion that the benzotriazolyl subunit is a better leaving group compared to chlorine [28]. The reaction of oxazolidine **6** with 2.3 equivalents of 7-chloro-4-hydrazinylquinoline in toluene at 90–95 °C for 30 h led to the formation of pyrazole **7** (yield 23%) together with considerable amounts of a resin (Scheme 5). We suggest that the lower yield of alcohol **7** is due to the poor leaving group quality of the formal alkoxide.

**Scheme 6:**
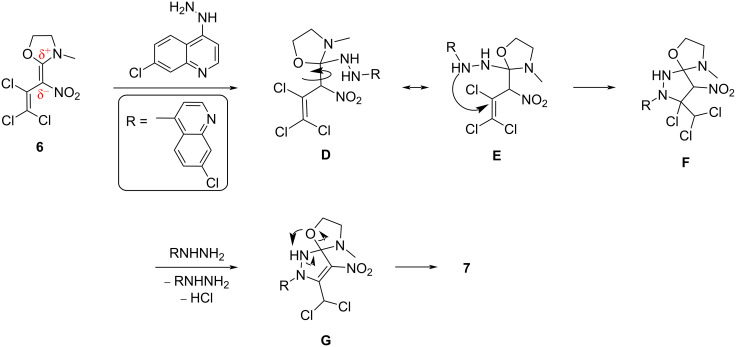
A plausible mechanism for the formation of pyrazole **7**.

A conceivable mechanistic pathway for the reaction cascade to pyrazole **7** is shown in Scheme 6. Initially, a nucleophilic attack of the NH_2_ group of 7-chloro-4-hydrazinylquinoline on the C2 position of the oxazolidine ring of **6** leads to the formation of imidoacetal **D**. Due to free rotation of the (O,N,N)C–C(NO_2_) axis the suitable conformer **E** can be formed. Thus, the less hindered NH group of intermediate **E** interacts with the C3 position giving an intramolecular, five-membered addition product **F**. Upon elimination of HCl from **F** by means of the second equivalent of 7-chloro-4-hydrazinylquinoline as base the triazaoxaspiro system **G** is formed. Finally, pyrazole **7** is obtained via intramolecular rearrangement of **G** under thermodynamic control.

**Scheme 7:**
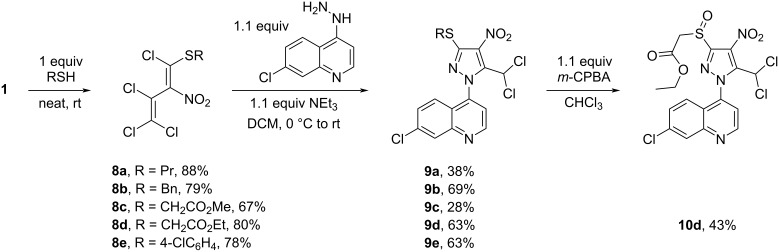
Synthesis of pyrazoles **9** and sulfoxide **10d**.

Further, the solventless treatment of nitrodiene **1** with equimolar amounts of thiols led to the formation of sulfanes **8a**–**e** in 67–88% yields (Scheme 7). The sulfanes **8a**–**e** were formed as single isomers, among them 1,3,4,4-tetrachloro-1-(4-chlorophenylsulfanyl)-2-nitrobuta-1,3-diene (**8e**) as *E*-isomer (X-ray) [29]. Thiodienes **8a**–**e** reacted with equimolar amounts of 7-chloro-4-hydrazinylquinoline in DCM at room temperature using triethylamine as base to give the corresponding 3-thiopyrazoles **9a**–**e** in moderate yields (28–69%). By oxidation of sulfane **9d** with *m*-chloroperbenzoic acid in chloroform at room temperature the sulfoxide **10d** was obtained in 43% yield (Scheme 7).

**Scheme 8:**
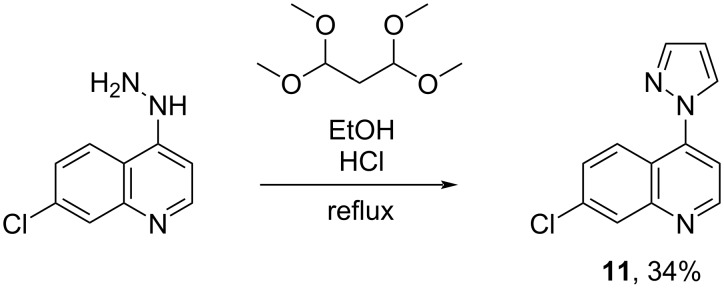
Synthesis of pyrazole **11**.

For comparing the biological activity of the newly synthesized, push–pull-substituted pyrazoles with the monosubstituted parent system, **11** was synthesized in 34% yield according to the literature [18] (Scheme 8). This proceeding corresponds well with our earlier publications on the microbiological activity of highly substituted pyrazoles [30–33]. Therefore, we hypothesize that the (NO_2_)C–C(CHCl_2_) and similar subunits could be valuable pharmacophores. Further investigations are on the way.

The persubstituted pyrazoles **3a**–**c**, **5a**–**o**, **7**, **9a**–**e**, and **10d** (total 25 examples) own unique substitution patterns. In total more than four million persubstituted pyrazoles are known, whereas the number of persubstituted 4-nitropyrazoles with 3-mercapto-, 3-amino-, and 3-amino-5-dihalomethyl substituents is limited to about one hundred for each case. 1-Heterocyclo-3-amino-4-nitro-5-dihalomethyl or 1-heterocyclo-3-thio-4-nitro-5-dihalomethyl representatives of persubstituted pyrazoles were unknown until now. In addition, it is known that polyhalogenated nitrobutadienes are versatile synthetic precursors for many bioactive heterocycles such as insecticidal neonicotinoids [34].

### Evaluation of biological properties

#### Antibacterial and cytotoxic properties

The general cytotoxic activities of compounds **3b** and **10d** were studied, as they influence the applicability of the compounds in other assays comprising mammalian cells. We incubated the murine fibroblast L929 cell line with different concentrations of the compounds for 72 h and quantified the residual viability of the cells. Non-linear regression of the dose–response data led to IC_50_ values of 1.4 µM for **3b** and of 0.6 µM for **10d**. Thus, for both compounds similar cytotoxic properties in the low µM range were determined.

The compounds were also tested in a viral infection model comprising SARS-CoV-2 and VeroE6 cells. However, no specific antiviral properties could be discovered, as the results were dominated by the cytotoxicity of the compounds. For details see Supporting Information File 1.

Furthermore, we evaluated the antibacterial properties of **3b** and **10d**. Growth of the Gram-positive bacterial strain *Staphylococcus aureus* was clearly inhibited with IC_50_ values of 15 µM (**3b**) and 30 µM (**10d**), whereas the growth of *Escherichia coli*, which was used as representative of Gram-negative strain, was hardly affected.

#### Antimalarial properties

The activity of the 26 pyrazoles was tested against the asexual blood stages of the malaria parasite *P. falciparum* (3D7 strain) using the SYBR Green I-based fluorescence assay [35]. Table 1 summarizes the activity of the compounds against the red blood cell stages of *P. falciparum* with EC_50_ values ranging from high nanomolar (200 nM) to low micromolar concentrations (4 µM). The compounds with the highest antimalarial activity were **3b**, **9e**, **3a**, and **10d** with EC_50_ values of 0.2 ± 0.1 µM, 0.2 ± 0.04 µM, 0.3 ± 0.1 µM and 0.34 ± 0.01 µM, respectively. Unsubstituted 1-(7-chloroquinolinyl)pyrazole **11** was the only compound of this series that showed no inhibition up to a tested concentration of 220 µM (50 µg/mL).

**Table 1:** EC_50_ values of newly synthesized inhibitors on *P. falciparum* 3D7.

compound	EC_50_ [µM]^a^

**3b**	0.2 ± 0.1
**9e**	0.2 ± 0.04
**3a**	0.3 ± 0.1
**10d**	0.34 ± 0.01
**3c**	0.41 ± 0.1
**9b**	0.44 ± 0.1
**5g**	0.46 ± 0.1
**5o**	0.46 ± 0.1
**9a**	0.48 ± 0.1
**5k**	0.52 ± 0.2
**5h**	0.61 ± 0.2
**5l**	0.83 ± 0.1
**5e**	0.9 ± 0.2
**5i**	0.97 ± 0.8
**9d**	1.1 ± 0.04
**5f**	1.1 ± 0.2
**5b**	1.2 ± 0.2
**5m**	1.27 ± 0.1
**5c**	1.28 ± 0.2
**7**	1.34 ± 0.5
**5d**	1.53 ± 0.4
**5n**	1.82 ± 0.3
**9c**	2.07 ± 0.3
**5j**	2.1 ± 0.3
**5a**	4.1 ± 1.0
**11**	nI^b^
AQ^c^	0.006 ± 0.001

^a^EC_50_ values were determined on *P. falciparum* 3D7 parasites using the SYBR Green I-based fluorescence assay. Values are expressed as mean ± SD from at least three independent determinations with different batches of inhibitors each including at least two measurements; ^b^nI = no Inhibition; ^c^AQ = amodiaquine.

Among the three 3-azolylpyrazoles **3a**–**c**, the triazole derivative **3b** showed the best activity with an EC_50_ = 0.2 ± 0.1 µM. The 3-aminopyrazoles **5a**–**o** showed a rather broad activity range (EC_50_ = 0.5–4.1 µM) and were all less active than the azolylpyrazoles **3**. The most active 3-aminopyrazoles **5** with very similar EC_50_ values of about 0.5 μM are representatives of the different classes of compounds: 3,4-dihydroisoquinoline **5g**, 4-fluorophenylpiperazine **5k**, and isoxazole **5o**. Furthermore, among the sulfides **9**, the 4-chlorophenylthiopyrazole **9e** stands out with an EC_50_ of 0.2 ± 0.04 µM. Interestingly, the oxidation of the sulfur atom in pyrazole **9d** (EC_50_ = 1.1 ± 0.04 µM) improved the antimalarial activity by a factor of more than three (sulfoxide **10d**, EC_50_ = 0.3 ± 0.01 µM).

In our experiments we used the established antimalarial drug amodiaquine as control. As shown in Table 1, the EC_50_ of amodiaquine was with 6 nM about two orders of magnitude more active than the newly synthesized and tested compounds.

## Conclusion

A two or three-step synthesis of novel 1-(7-chloroquinolin-4-yl)-3(5)-R-4-nitro-5(3)-(dichloromethyl)-1*H*-pyrazoles **3a**–**c**, **5a**–**o**, and **9a**–**e** has been developed, starting from our synthetic building block 2-nitroperchlorobutadiene (**1**). 3-(Alkyl)(2-hydroxyethyl)aminopyrazoles **7** are accessible from the reaction of oxazolidine derivative **6** with 7-chloro-4-hydrazinylquinoline. Oxidation of sulfane **9d** led to the formation of the pharmacologically interesting sulfoxide **10d**. The newly formed pyrazoles deserve additional synthetic interest as starting materials, also due to their nitro and dichloromethyl groups.

All persubstituted pyrazoles tested in this study showed pronounced antimalarial activity against the asexual blood stages of *P. falciparum* with EC_50_ values ranging from 200 nM to 4 µM (Table 1). Thus, the antiparasitic activity of persubstituted pyrazoles is about two orders of magnitude lower compared to the antimalarial amodiaquine (EC_50_ = 6 nM). Also many other antimalarials such as chloroquine, quinine, mefloquine, and artesunate, tested in the SYBR Green or comparable test systems, show EC_50_ values in the lower nM range [36–39]. Thus, the newly reported compounds are a valuable enrichment from the chemical perspective and have promising potential for further optimization or combination with other compounds.

## Supporting Information

File 1 Experimental procedures, characterization data (^1^H, ^13^C, ^14^N, ^15^N NMR, IR, MS and HRMS), copies of spectra, and detailed procedures of biological assays.
