# Supplementary material for: Chemistry of polyhalogenated nitrobutadienes, 17: Efficient synthesis of persubstituted chloroquinolinyl-1H-pyrazoles and evaluation of their antimalarial, anti-SARS-CoV-2, antibacterial, and cytotoxic activities
Source: Beilstein J Org Chem. 2022 May 9;18:524–32. doi: 10.3762/bjoc.18.54 (PMC9112185; doi:10.3762/bjoc.18.54)
Supplement: File 1 — Experimental procedures, characterization data (1H, 13C, 14N, 15N NMR, IR, MS and HRMS), copies of spectra, and detailed procedures of biological assays. [file Beilstein_J_Org_Chem-18-524-s001.pdf]

BEILSTEIN JOURNAL OF ORGANIC CHEMISTRY

Supporting Information

for

Chemistry of polyhalogenated nitrobutadienes, 17: Efficient synthesis of persubstituted chloroquinolinyl-1*H*-pyrazoles and evaluation of their antimalarial, anti-SARS-CoV-2, antibacterial, and cytotoxic activities

Viktor A. Zapol'skii, Isabell Berneburg, Ursula Bilitewski, Melissa Dillenberger, Katja Becker, Stefan Jungwirth, Aditya Shekhar, Bastian Krueger and Dieter E. Kaufmann

Beilstein J. Org. Chem. **2022,** *18,* 524–532. doi:10.3762/bjoc.18.54

Experimental procedures, characterization data (^{1}H, ^{13}C, ^{14}N, ^{15}N NMR, IR, MS and HRMS), copies of spectra, and detailed procedures of biological assays

License and Terms: This is a supporting information file under the terms of the Creative Commons Attribution License (https://creativecommons.org/licenses/by/4.0). Please note that the reuse, redistribution and reproduction in particular requires that the author(s) and source are credited and that individual graphics may be subject to special legal provisions.
The license is subject to the *Beilstein Journal of Organic Chemistry* terms and conditions: (https://www.beilstein-journals.org/bjoc/terms)

Table of contents

1. General methods (chemical synthesis)..S2

2. Experimental procedures and characterization data...S3

3. Copies of ^{1}H, ^{13}C, ^{14}N, ^{15}N NMR, and HRMS spectra..S25

4. Experimental procedures of biological assays S58

5. Table S1: Predicted and found (100 MHz) ^{13}C NMR shifts for

pyrazoles **5a**,**b** S62

6. References...S63

1. General methods (chemical synthesis).

All chemicals were purchased and used without further purification unless otherwise mentioned. TLC was performed with Merck aluminium-backed TLC plates with silica gel 60, F254. Flash column chromatography was performed with Macherey–Nagel silica gel 60 M (0.040–0.063 mm) with appropriate mixtures of petroleum ether (PE, boiling range 60–70 °C) and ethyl acetate as eluents. Melting points were determined in capillary tubes with a Büchi B-520 instrument and were not corrected. The ATR-IR spectra were measured on Bruker "Alpha-T" spectrometer with a Bruker "Alpha Platinum ATR" single reflection diamond ATR module in the range of 400 to 4000 cm^{-1}. ^{1}H NMR and ^{13}C NMR spectra at 600 and 150 MHz, respectively, were recorded with an "Avance III" 600 MHz FT–NMR spectrometer (Bruker, Rheinstetten, Germany). ^{1}H NMR and ^{13}C NMR spectra at 400 and 100 MHz, respectively, were recorded with an "Avance" 400 MHz FT–NMR spectrometer (also Bruker). ^{14}N and ^{15}N NMR spectra were measured at their appropriate resonance frequency on the aforementioned spectrometers; ^{15}N measurements were taken as gs-^{1}H,^{15}N–HSQC or HMBC experiments with inverse detection. ^{1}H and ^{13}C NMR spectra were referenced to the residual solvent peak: $CDCl_3$, δ = 7.26 (^{1}H) and 77.0 ppm (^{13}C); DMSO-d_6, δ = 2.50 (^{1}H), and 39.7 ppm (^{13}C). Mass spectra were obtained with a Hewlett–Packard MS 5989B spectrometer, usually in direct mode with electron impact ionization (70 eV). For chlorinated compounds, all peak values of molecular ions and fragments refer to the isotope ^{35}Cl. The HRMS spectra were obtained with a Bruker Impact II, a Bruker Daltonik Tesla-Fourier transform-ion cyclotron resonance mass spectrometer.

2. Experimental procedures and characterization data (chemical synthesis)

Pentachloro-2-nitro-1,3-butadiene (**1**) was prepared from 2*H*-pentachloro-1,3-butadiene in 53% yield (b.p. 69–71 °C / 1 mbar) according to the literature [1,2]. The dimerization of trichloroethene was performed using the optimized procedure [3]. 1,1-Bis(1*H*-pyrazol-1-yl)-3,4,4-trichloro-2-nitrobuta-1,3-diene (**2a**, 92%), 1,1-bis(1*H*-1,2,4-triazol-1-yl)-3,4,4-trichloro-2-nitrobuta-1,3-diene (**2b**, 95%), and 1,1-bis(1*H*-benzotriazol-1-yl)-3,4,4-trichloro-2-nitrobuta-1,3-diene (**2c**, 98%) were prepared from pentachloro-2-nitro-1,3-butadiene (**1**) and pyrazole, 1,2,4-triazole, and benzotriazole, respectively, similar to literature [4].

7-Chloro-4-(5'-(dichloromethyl)-4'-nitro-1'*H*-[1,3'-bipyrazol]-1'-yl)quinoline (**3a**). At 0 °C 7-chloro-4-hydrazinylquinoline (213 mg, 1.10 mmol) was added to a suspension of diene **2a** (335 mg, 1.00 mmol) in 10 mL methanol portionwise within 5 min. At first, the resulting mixture was stirred for 1 h at 0 °C, then the cooling bath was removed. After 5 h at room temperature (rt), the reaction mixture was cooled to 0 °C and a solution of triethylamine (111 mg, 1.10 mmol) in 1 mL methanol was added dropwise within 5 min. After stirring for 10 h at rt, water (50 mL) and 10 drops of conc. hydrochloric acid were added. After extraction with dichloromethane (3 × 20 mL), treatment of the combined organic layers twice with water (20 mL each portion), and drying with anhydrous calcium chloride the obtained product was purified by flash column chromatography (petroleum ether/ethyl acetate 2:1) and finally dried *in vacuo* to yield pyrazole **3a** (271 mg, 0.64 mmol, 64%) as light yellow solid. M.p. 205–206 °C. IR: $\tilde{\nu}_{max}$ 2940, 1581, 1533, 1391, 1041, 932, 759 cm^{-1}. ^1H NMR (400 MHz, $CDCl_3$): δ 9.16 (d, J = 4.6 Hz, 1 H, CH; $^1J_{C\text{-}H}$ = 184.3 Hz), 8.28 (d, J = 2.0 Hz, 1 H, CH; $^1J_{C\text{-}H}$ = 170.0 Hz), 8.03 (d, J = 2.6 Hz, 1 H, CH), 7.84 (d, J = 1.6 Hz, 1 H, CH), 7.78 (d, J = 4.6 Hz, 1 H, CH), 7.60 (dd, J = 2.0, 9.0 Hz, 1 H, CH), 7.44 (d, J = 9.0 Hz, 1 H, CH), 7.41

(s, $^{1}J_{C-H}$ = 183.0 Hz, 1H, $CHCl_2$), 6.55 (dd, J = 2.0, 2.6 Hz, 1 H, CH; $^{1}J_{C-H}$ = 179.8 Hz) ppm. ^{13}C NMR (100 MHz, $CDCl_3$): δ 151.0 (CH), 150.0, 143.3, 143.2 (CH), 141.7, 140.4, 137.2, 131.0 (CH), 129.9 (CH), 129.1 (CH), 124.0 (CH), 123.6 (CNO_2), 122.7, 120.5 (CH), 108.4 (CH), 56.8 ($CHCl_2$) ppm. MS: *m*/*z* (%) = 422 [M^+] (95), 405 $[M\text{-}OH]^+$ (4), 387 $[M^+\text{-}Cl]^+$ (58), 339 $[M\text{-}CHCl_2]^+$ (20), 322 (45), 162 [7-chloroquinolin-4-ylium]$^+$ (93), 99 (100). HRMS (ESI): calcd. for $C_{32}H_{18}Cl_6N_{12}O_4Na$ $[2M + Na]^+$: 868.9568; found: 868.9586.

7-Chloro-4-(5-(dichloromethyl)-4-nitro-3-(1*H*-1,2,4-triazol-1-yl)-1*H*-pyrazol-1-yl)quinoline (**3b**). Compound **3b** was synthesized from diene **2b** (337 mg, 1.00 mmol), 7-chloro-4-hydrazinylquinoline (213 mg, 1.10 mmol) and triethylamine (111 mg, 1.10 mmol) according to the procedure employed for compound **3a**. After addition of a solution of triethylamine in methanol the resulting mixture was stirred for 2 d at rt. Yield: 246 mg (0.58 mmol, 58%). M.p. 209–210 °C; colorless solid. IR: $\tilde{\nu}_{max}$ 2964, 1578, 1500, 1323, 1076, 832, 755 cm^{-1}. ^{1}H NMR (400 MHz, $CDCl_3$): δ 9.18 (d, J = 4.6 Hz, 1 H, CH; $^{1}J_{C-H}$ = 185.1 Hz), 8.73 (s, $^{1}J_{C-H}$ = 217.8 Hz, 1H, CH_{triaz}), 8.30 (d, J = 2.0 Hz, 1 H, CH; $^{1}J_{C-H}$ = 169.4 Hz), 8.21 (s, $^{1}J_{C-H}$ = 211.0 Hz, 1H, CH_{triaz}), 7.79 (d, J = 4.6 Hz, 1 H, CH), 7.62 (dd, J = 2.0, 9.0 Hz, 1 H, CH), 7.50 (s, 1 H, $CHCl_2$), 7.39 (d, J = 9.0 Hz, 1 H, CH) ppm. ^{13}C NMR (100 MHz, $CDCl_3$): δ 153.5 (CH), 151.0 (CH), 150.0, 145.0 (CH), 141.4, 141.2, 140.4, 137.4, 130.2 (CH), 129.3 (CH), 123.8 (CNO_2), 123.6 (CH), 122.4, 120.0 (CH), 56.6 ($CHCl_2$) ppm. MS: *m*/*z* (%) = 423 [M^+] (77), 406 $[M\text{-}OH]^+$ (3), 388 $[M\text{-}Cl]^+$ (59), 371 $[M\text{-}Cl\text{-}OH]^+$ (17), 340 $[M\text{-}CHCl_2]^+$ (18), 325 (20), 162 [7-chloroquinolin-4-ylium]$^+$ (100). HRMS (ESI): calcd. for $C_{15}H_8Cl_3N_7O_2Na$ $[M + Na]^+$: 445,9697; found: 445.9702.

4-(3-(1*H*-Benzotriazol-1-yl)-5-(dichloromethyl)-4-nitro-1*H*-pyrazol-1-yl)-7-chloroquinoline (**3c**). Compound **3c** was synthesized from diene **2c** (437 mg, 1.00 mmol), 7-chloro-4-hydrazinylquinoline (213 mg, 1.10 mmol) and triethylamine (111 mg, 1.10 mmol) according to the procedure employed for compound **3a**. After addition of a solution of triethylamine in methanol the resulting mixture was stirred for 2 d at rt. Yield: 285 mg (0.69 mmol, 69%). M.p. 198–199 °C; colorless solid. IR: $\tilde{\nu}_{max}$ 3006, 1575, 1511, 1375, 1047, 827, 744 cm^{-1}. ^{1}H NMR (400 MHz, $CDCl_3$): δ 9.20 (d, *J* = 4.6 Hz, 1 H, CH), 8.31 (d, *J* = 2.1 Hz, 1 H, CH), 8.19 (d, *J* = 8.3 Hz, 1 H, $CH_{benzotriaz}$), 7.88 (d, *J* = 4.6 Hz, 1 H, CH), 7.66 (d, *J* = 8.3 Hz, 1 H, $CH_{benzotriaz}$), 7.65 (dd, *J* = 2.1, 8.9 Hz, 1 H, CH), 7.59 (ddd, *J* = 8.3, 7.0, 1.1 Hz, 1 H, $CH_{benzotriaz}$), 7.57 (s, 1 H, $CHCl_2$), 7.49 (ddd, *J* = 8.0, 7.7, 0.9 Hz, 1 H, $CH_{benzotriaz}$), 7.48 (d, *J* = 8.9 Hz, 1 H, CH) ppm. ^{13}C NMR (100 MHz, $CDCl_3$): δ 151.1 (CH), 150.1, 145.6, 141.7, 141.1, 140.3, 137.3, 132.7, 130.1 (CH), 129.5 (CH), 129.3 (CH), 125.2 (CH), 124.4 (CNO_2), 123.7 (CH), 122.6, 120.6 (CH), 120.5 (CH), 110.5 (CH), 56.7 ($CHCl_2$) ppm. MS: *m*/*z* (%) = 473 [M^+] (7), 445 $[M-N_2]^+$ (2), 399 $[M-N_2-NO_2]^+$ (3), 364 $[M-N_2-NO_2-Cl]^+$ (7), 329 $[M-N_2-NO_2-2Cl]^+$ (10), 162 [7-chloroquinolin-4-ylium]$^+$ (21). HRMS (ESI): calcd. for $C_{19}H_{10}Cl_3N_7O_2Na$ $[M + Na]^+$: 495.9854; found: 495.9856.

1-(1*H*-Benzotriazol-1-yl)-1-methyamino-3,4,4-trichloro-2-nitrobuta-1,3-diene (4a). At 0 °C, a suspension of 4.37 g (10.0 mmol) bis(benzotriazolyl) compound **2c** in 50 mL of MeOH was treated with a solution of 1.03 g (11.0 mmol) methylamine (33% in ethanol) in 5 mL MeOH. The resulting mixture was kept at 0 °C for 1 h and then at rt for 5 h. After cooling to 10 °C, a solution of 5 mL hydrochloric acid in 250 mL of cold water was added under stirring. After 1 h, the precipitate was isolated and washed with HCl (5%, 30 mL), cold water (2 × 50 mL), and cold methanol (5 mL). Drying under reduced pressure afforded the diene **4a** (2.97 g, 85%) as yellow solid, as a mixture of

Z- and *E*-isomers (1:1). M.p. 133–135 °C. IR: $\tilde{\nu}_{max}$ 3181, 1626, 1587 (NO_2), 1492, 1361 (NO_2), 1297, 1197, 1036, 1004, 906, 837, 743, 431 cm^{-1}. ^1H NMR (400 MHz, $CDCl_3$): δ 10.43 (br s, 1 H, NH), 10.36 (br s, 1 H, NH), 8.22 (d, *J* = 8.3 Hz, 1 H, CH), 8.20 (d, *J* = 8.1 Hz, 1 H, CH), 7.69 (d, *J* = 8.3 Hz, 1 H, CH), 7.68 (d, *J* = 8.3 Hz, 1 H, CH), 7.63–7.51 (m, 4 H, CH), 2.90 (d, *J* = 5.3 Hz, 3 H, Me), 2.80 (d, *J* = 5.5 Hz, 3 H, Me) ppm. ^{13}C NMR (100 MHz, $CDCl_3$): δ 150.5 (C1), 150.1 (C1), 145.5, 145.2, 132.2, 132.1, 130.1 (CH), 130.0 (CH), 128.2, 128.0, 125.7 (2 CH), 121.1 (CH), 120.9 (CH), 116.0, 114.6, 109.8 (CH), 109.5 (CH), 31.3 (Me), 31.0 (Me) ppm. C-atoms of C-NO_2 – group could not be detected. MS: *m*/*z* (%) = 347 [M^+] (3), 312 $[M-Cl]^+$ (7), 237 $[M-Cl-NO_2-NHMe+H]^+$ (17), 119 [benzotriazole] [100). HRMS (ESI): calcd. for $C_{11}H_8Cl_3N_5O_2Na$ $[M + Na]^+$: 369.9636; found: 369.9640.

2-(1-(1*H*-Benzotriazol-1-yl)-3,4,4-trichloro-2-nitrobuta-1,3-dien-1-yl)-1,2,3,4-tetrahydroisoquinoline (4g). Compound **4g** was synthesized from diene **2c** and 1,2,3,4-tetrahydroisoquinoline according to the procedure employed for compound **4a**. Yield: 70%. M.p. 155–156 °C; yellow solid. IR: $\tilde{\nu}_{max}$ 3027, 1594, 1558, 1500, 1287, 1202, 1040, 906, 873, 789 cm^{-1}. ^1H NMR (200 MHz, $CDCl_3$): δ 8.16 (d, *J* = 7.8 Hz, 1 H, CH), 7.70–7.42 (m, 3 H), 7.35–6.95 (m, 4 H), 4.63 (br s, 2 H, CH_2), 3.82–2.90 (m, 4 H, 2 CH_2) ppm. ^{13}C NMR (50 MHz, $CDCl_3$): δ 147.6, 146.2, 132.7, 132.4, 130.6 (CH), 130.0, 128.7 (CH), 127.8 (CH), 127.1 (CH), 126.1 (CH), 125.9 (CH), 125.1, 124.2, 121.0 (CH), 120.6, 110.4 (CH), 52.3 (CH_2), 48.9 (CH_2), 29.1 (CH_2) ppm. MS: *m*/*z* (%) = 449 [M^+] (3), 375 $[M-N_2-NO_2]^+$ (2), 304 (25), 285 $[M\text{-benzotriazole-}NO_2]^+$ (17), 132 [tetrahydroisoquinoline] (50). HRMS (ESI): calcd. for $C_{19}H_{14}Cl_3N_5O_2Na$ $[M + Na]^+$: 472.0105; found: 472.0101.

2-(1-(1*H*-Benzotriazol-1-yl)-3,4,4-trichloro-2-nitrobuta-1,3-dien-1-yl)-1-methyl-1,2,3,4-tetrahydroisoquinoline (4h) was synthesized from diene **2c** and 1-methyl-1,2,3,4-tetrahydroisoquinoline according to the procedure employed for compound **4a** using purification with column chromatography (petroleum ether/ethyl acetate 10:1). Yield: 63%. M.p. 143–144 °C; yellow solid. IR: $\tilde{\nu}_{max}$ 2835, 1599, 1559, 1451, 1284, 1045, 828, 741 cm^{-1}. Compound **4h** is only stable for a short time in organic solvents. ^{1}H NMR (400 MHz, CD_2Cl_2): δ 8.16 (d, J = 7.9 Hz, 1 H, CH), 7.85–7.40 (m, 3 H), 7.38–6.77 (m, 4 H), 4.01 (br s, 1H, CH), 3.67–2.88 (m, 4 H, 2 CH_2), 1.61 (br s, 3 H, CH_3) ppm. ^{13}C NMR (100 MHz, CD_2Cl_2): δ 147.3, 146.5, 134.8, 130.5, 130.1 (CH), 129.1 (CH), 129.0, 127.6 (CH), 127.0 (CH), 126.3, 126.2 (CH), 126.0 (CH), 125.3, 121.3, 121.0 (CH), 110.7 (CH), 57.4 (CH), 46.2 (CH_2), 29.7 (CH_2), 24.9 (CH_3) ppm. MS: m/z (%) = 463 [M^+] (2), 317 [M- methyltetrahydroisoquinoline]$^+$ (3), 299 [M-benzotriazole-NO_2]$^+$ (8), 284 [M-benzotriazole-NO_2-Me]$^+$ (18). HRMS (ESI): calcd. for $C_{20}H_{16}Cl_3N_5O_2Na$ [M + Na]$^+$: 486.0262; found: 486.0266.

1-(1*H*-Benzotriazol-1-yl)-3,4,4-trichloro-*N*-(2-fluorobenzyl)-2-nitrobuta-1,3-dien-yl-1-amine (4l) was synthesized from diene **2c** and 2-fluorobenzylamine according to the procedure employed for compound **4a** using purification with column chromatography (petroleum ether/ethyl acetate 2:1). Yield: 52%, mixture of two rotamers (1: 1). M.p. 134–135 °C; yellow solid. IR: $\tilde{\nu}_{max}$ 2980, 1614, 1584, 1438, 1176, 1038, 825, 741 cm^{-1}. ^{1}H NMR (400 MHz, $CDCl_3$): δ 10.61 (br s, 1 H, NH), 8.22–814 (m, 1 H, CH), 7.65–7.44 (m, 3 H), 7.32–7.21 (m, 1 H), 7.06–6.82 (m, 3H), 4.41–4.14 (m, 2H, CH_2) ppm. ^{13}C NMR (100 MHz, $CDCl_3$): δ 160.7 (CF, $^{1}J_{(C-F)}$ = 249.3 Hz), 160.6 (CF, $^{1}J_{(C-F)}$ = 249.8 Hz), 149.2, 148.8, 147.3, 145.5, 145.2, 132.4, 130.9 (CH, $^{3}J_{(C-F)}$ = 7.9 Hz), 129.9 (CH), 129.7 (CH), 129.6 (CH), 125.6 (CH), 124.7 (CH), 121.6 (Cq, $^{2}J_{(C-F)}$ = 14.9 Hz), 120.9 (CH), 120.6 (CH), 118.2, 115.9 (CH, $^{2}J_{(C-F)}$ = 21.5 Hz), 110.0 (CH),

109.7 (CH), 43.2 (CH_2), 43.0 (CH_2) ppm. MS: *m*/*z* (%) = 441 [M^+] (1), 405 $[M\text{-}HCl]^+$ (3), 331 $[M\text{-}ArCH_2\text{-}H]^+$ (2), 119 [benzotriazole] (7), 109 $[o\text{-}F\text{-}C_6H_4\text{-}CH_2]^+$ (100). HRMS (ESI): calcd. for $C_{17}H_{11}Cl_3FN_5O_2Na$ $[M + Na]^+$: 463.9855; found: 463.9850.

(*E*)-*N*-(1-(1*H*-Benzotriazol-1-yl)-3,4,4-trichloro-2-nitrobuta-1,3-dien-1-yl)-5-methylisoxazolyl-3-amine (4o). At rt, to a solution of 4.37 g (10.0 mmol) bis(benzotriazolyl) compound **2c** in 50 mL of tetrahydrofuran was added 5-methylisoxazolyl-3-amine (1.47 g, 15.0 mmol) portionwise. The resulting mixture was kept at rt for 3 d and then the solvent was evaporated *in vacuo*. To the resulting oil was added 40 mL cold methanol and then dropwise 5 mL conc. hydrochloric acid under stirring. After 1 h, the precipitate was isolated and washed with cold water (2 × 50 mL), and cold methanol (10 mL). Drying under reduced pressure afforded the diene **4o** (2.33 g, 56%), mp 134–135°C; yellow solid. IR: $\tilde{\nu}_{max}$ 3119, 1608, 1477, 1348, 1180, 1035, 858, 546 cm^{-1}. ^1H NMR (400 MHz, $CDCl_3$): δ 11.43 (br s, 1 H, NH), 8.15 (d, *J* = 8.3 Hz, 1 H, CH), 7.59 (ddd, *J* = 8.3, 6.9, 1.1 Hz, 1 H, CH), 7.52–7.40 (m, 2 H, CH), 4.77 (d, *J* = 0.7 Hz, $^1J_{C\text{-}H}$ = 184.8 Hz, 1 H, isoxazole-H), 2.12 (d, *J* = 0.7 Hz, 3 H, Me) ppm. ^{13}C NMR (100 MHz, $CDCl_3$): δ 172.0 (Me-<u>C</u>), 155.9 (NH-C), 145.5, 143.9, 132.4, 130.2 (CH), 125.8 (CH), 122.1 ($C\text{-}NO_2$), 120.9 (CH), 119.4, 119.2, 109.7 (CH), 95.2 (CH), 12.5 (Me) ppm. MS: *m*/*z* (%) = 414 [M^+] (0.3), 332 [M-methylisoxazole]$^+$ (0.5), 252 $[M\text{-benzotriazole-}NO_2]^+$ (4), 119 [benzotriazole] (100). HRMS (ESI): calcd. for $C_{14}H_9Cl_3N_6O_3Na$ $[M + Na]^+$: 436.9694; found: 436.9696.

1-(7-Chloroquinolin-4-yl)-3-(dichloromethyl)-*N*-methyl-4-nitro-1*H*-pyrazolyl-5-amine (5a). At 0 °C, 7-chloro-4-hydrazinylquinoline (445 mg, 2.30 mmol) was added to a suspension of diene **4a** (349 mg, 1.00 mmol) in 10 mL methanol portionwise within 5 min. The resulting mixture was stirred for 2 h at 0 °C, then for 12 h at rt, and

additionally 6 h under reflux. After cooling to rt, water (50 mL) and 10 drops of conc. hydrochloric acid were added under stirring. After extraction with dichloromethane (3 × 20 mL), treatment of the combined organic layers twice with water (20 mL each portion), and drying with anhydr. calcium chloride the obtained product was purified by flash column chromatography (petroleum ether/ethyl acetate 2:1) and finally dried *in vacuo* to yield pyrazole **5a** (147 mg, 38%). M.p. 195–196 °C; light brown solid. IR: $\tilde{\nu}_{max}$ 3362, 1628, 1528, 1449, 1348, 1120, 867, 739 cm^{-1}. ^1H NMR (400 MHz, $CDCl_3$): δ 9.09 (d, J = 4.5 Hz, 1 H, CH; $^1J_{C\text{-}H}$ = 183.6 Hz), 8.25 (d, J = 2.2 Hz, 1 H, CH; $^1J_{C\text{-}H}$ = 169.5 Hz), 7.69 (d, J = 8.7 Hz, 1 H, CH), 7.65 (dd, J = 9.0, 1.9 Hz, 1 H, CH), 7.56 (d, J = 4.6 Hz, 1 H, CH), 7.41 (q, J = 5.6 Hz, 1H, NH), 7.32 (s, 1 H, $CHCl_2$), 2.46 (d, J = 5.6 Hz, $^1J_{C\text{-}H}$ = 140.3 Hz, 3 H, Me) ppm. ^{13}C NMR (100 MHz, $CDCl_3$): δ 151.3 (CH), 149.9, 149.4, 147.2, 142.4, 137.3, 130.0 (CH), 129.2 (CH), 123.9 (CH), 123.0, 119.6 (CH), 114.7 (CNO_2), 62.3 ($CHCl_2$), 31.3 (Me) ppm. MS: m/z (%) = 385 [M^+] (85), 368 $[M\text{-}OH]^+$ (15), 350 $[M\text{-}Cl]^+$ (30), 304 $[M\text{-}Cl\text{-}NO_2]^+$ (20), 162 [7-chloroquinolin-4-ylium] (85), 99 (100). HRMS (ESI): calcd. for $C_{14}H_{10}Cl_3N_5O_2Na$ $[M + Na]^+$: 407.9792; found: 407.9790.

1-(7-Chloroquinolin-4-yl)-3-(dichloromethyl)-*N,N*-dimethyl-4-nitro-1*H*-pyrazolyl-5-amine (5b) was synthesized from diene **4b** and 7-chloro-4-hydrazinylquinoline according to the procedure employed for compound **5a** (reflux in methanol 16 h) using purification with column chromatography (petroleum ether/ethyl acetate 5:1). Yield: 65%. M.p. 141–142 °C; yellow solid. IR: $\tilde{\nu}_{max}$ 3004, 1560, 1486, 1344, 1071, 823, 752 cm^{-1}. ^1H NMR (600 MHz, $CDCl_3$): δ 9.10 (d, J = 4.6 Hz, 1 H, CH; $^1J_{C\text{-}H}$ = 183.5 Hz), 8.23 (d, J = 1.9 Hz, 1 H, CH; $^1J_{C\text{-}H}$ = 169.4 Hz), 7.73 (d, J = 4.5 Hz, 1 H, CH; $^1J_{C\text{-}H}$ = 164.5 Hz), 7.56 (dd, J = 9.0, 2.0 Hz, 1 H, CH; $^1J_{C\text{-}H}$ = 177.7 Hz), 7.50 (s, 1 H, $CHCl_2$; $^1J_{C\text{-}H}$ = 183.7 Hz), 7.44 (d, J = 9.0 Hz, 1 H, CH; $^1J_{C\text{-}H}$ = 167.0 Hz), 2.96 (s, $^1J_{C\text{-}H}$ = 137.4 Hz, 6 H, 2 Me) ppm. ^{13}C NMR (150 MHz, $CDCl_3$): δ 154.0, 151.1 (CH), 150.0, 142.6,

140.4, 136.7, 129.3 (CH), 128.9 (CH), 124.6 (CH), 123.0, 121.6 (CNO_2), 120.4 (CH), 57.7 ($CHCl_2$), 41.5 (Me) ppm. ^{14}N NMR (43.38 MHz, $CDCl_3$): δ -16.3 (NO_2) ppm. ^{15}N,^{1}H HMBC NMR (43.38 MHz, $CDCl_3$): δ -327.0 (NMe_2), -180.5 (pyrazole–N_1), -90.3 (pyrazole–N_2), -52.9 (pyridine-N). MS: *m/z* (%) = 399 [M^+] (40), 382 $[M\text{-}OH]^+$ (40), 364 $[M\text{-}Cl]^+$ (7), 354 $[M\text{-}HNMe_2]^+$ (21), 318 $[M\text{-}Cl\text{-}NO_2]^+$ (10), 162 [7-chloroquinolin-4-ylium]$^+$ (82), 113 (100). HRMS (ESI): calcd. for $C_{15}H_{12}Cl_3N_5O_2Na$ $[M + Na]^+$: 421.9945; found: 421.9944.

7-Chloro-4-(5-(dichloromethyl)-4-nitro-3-(pyrrolidin-1-yl)-1*H*-pyrazol-1-yl)quinoline (5c) was synthesized from diene **4c** and 7-chloro-4-hydrazinylquinoline according to the procedure employed for compound **5a** (reflux in methanol 10 h) using purification with column chromatography (petroleum ether/ethyl acetate 5:1). Yield: 44%. M.p. 164–165 °C; yellow solid. IR: $\tilde{\nu}_{max}$ 2970, 1573, 1478, 1308, 1177, 819, 731 cm^{-1}. ^{1}H NMR (400 MHz, $CDCl_3$): δ 9.10 (d, *J* = 4.6 Hz, 1 H, CH), 8.23 (d, *J* = 1.8 Hz, 1 H, CH), 7.74 (d, *J* = 4.6 Hz, 1 H, CH), 7.56 (dd, *J* = 9.0, 2.0 Hz, 1 H, CH), 7.49 (d, *J* = 9.0 Hz, 1 H, CH), 7.44 (s, 1 H, $CHCl_2$), 3.52–3.36 (m, 4 H, 2 NCH_2), 2.05–1.91 (m, 4 H, 2 CH_2) ppm. ^{13}C NMR (100 MHz, $CDCl_3$): δ 151.1 (CH), 151.0, 150.1, 142.7, 139.5, 136.7, 129.3 (CH), 128.9 (CH), 124.8 (CH), 123.2, 120.6 (CNO_2), 120.4 (CH), 57.7 ($CHCl_2$), 50.1 (NCH_2), 25.5 (CH_2) ppm. MS: *m/z* (%) = 425 [M^+] (17), 408 $[M\text{-}OH]^+$ (100), 390 $[M\text{-}Cl]^+$ (10), 379 $[M\text{-}NO_2]^+$ (15), 295 $[M\text{-}CHCl_2\text{-}HNO_2]^+$ (8), 162 [7-chloroquinolin-4-ylium]$^+$ (60). HRMS (ESI): calcd. for $C_{17}H_{14}Cl_3N_5O_2Na$ $[M + Na]^+$: 448.0105; found: 448.0109.

7-Chloro-4-(5-(dichloromethyl)-4-nitro-3-(piperidin-1-yl)-1*H*-pyrazol-1-yl)quinoline (5d) was synthesized from diene **4d** and 7-chloro-4-hydrazinylquinoline according to the procedure employed for compound **5a** (reflux in methanol 20 h) using

purification with column chromatography (petroleum ether/ethyl acetate 3:1). Yield: 61%. M.p. 166–167 °C; yellow solid. IR: $\tilde{\nu}_{max}$ 2938, 1559, 1496, 1345, 1073, 821, 741 cm^{-1}. ^1H NMR (400 MHz, $CDCl_3$): δ 9.11 (d, *J* = 4.6 Hz, 1 H, CH), 8.24 (d, *J* = 2.0 Hz, 1 H, CH), 7.74 (d, *J* = 4.6 Hz, 1 H, CH), 7.57 (s, 1 H, $CHCl_2$), 7.56 (dd, *J* = 8.9, 2.0 Hz, 1 H, CH), 7.42 (d, *J* = 8.9 Hz, 1 H, CH), 3.34–3.13 (m, 4 H, 2 NCH_2), 1.85–1.74 (m, 4 H, 2 CH_2), 1.64–1.55 (m, 2 H, CH_2) ppm. ^{13}C NMR (100 MHz, $CDCl_3$): δ 154.0, 151.1 (CH), 150.1, 142.7, 140.6, 136.8, 129.4 (CH), 128.9 (CH), 124.5 (CH), 123.1, 122.1 (CNO_2), 120.4 (CH), 57.7 ($CHCl_2$), 50.9 (2 NCH_2), 25.4 (2 CH_2), 24.0 (CH_2) ppm. MS: *m*/*z* (%) = 439 [M^+] (18), 422 $[M-OH]^+$ (100), 405 $[M-Cl]^+$ (25), 393 $[M-NO_2]^+$ (5), 334 (25), 162 [7-chloroquinolin-4-ylium]$^+$ (50). HRMS (ESI): calcd. for $C_{18}H_{16}Cl_3N_5O_2Na$ $[M + Na]^+$: 462.0262; found: 462.0268.

Ethyl 1-(1-(7-chloroquinolin-4-yl)-5-(dichloromethyl)-4-nitro-1*H*-pyrazol-3-yl)piperidine-4-carboxylate (5e) was synthesized from diene **4e** and 7-chloro-4-hydrazinylquinoline according to the procedure employed for compound **5a** (reflux in ethanol 12 h) using purification with column chromatography (petroleum ether/ethyl acetate 5:1). Yield: 57%. M.p. 148–149 °C; yellow solid. IR: $\tilde{\nu}_{max}$ 2943, 1724 (C=O), 1551, 1450, 1336, 1171, 1045, 826, 752 cm^{-1}. ^1H NMR (400 MHz, $CDCl_3$): δ 9.11 (d, *J* = 4.6 Hz, 1 H, CH), 8.24 (d, *J* = 1.9 Hz, 1 H, CH), 7.74 (d, *J* = 4.6 Hz, 1 H, CH), 7.57 (dd, *J* = 8.9, 1.9 Hz, 1 H, CH), 7.55 (s, 1 H, $CHCl_2$), 7.40 (d, *J* = 9.0 Hz, 1 H, CH), 4.15 (q, *J* = 7.1 Hz, 2 H, OCH_2), 3.65 (ddd, *J* = 12.8, 3.5, 3.5 Hz, 2 H, NCH_2), 2.95 (ddd, *J* = 12.8, 10.6, 2.5 Hz, 2 H, NCH_2), 2.54–2.45 (m, 1 H, CH), 2.07–1.90 (m, 4 H, 2 CH_2), 1.26 (t, *J* = 7.1 Hz, 3 H, CH_3) ppm. ^{13}C NMR (100 MHz, $CDCl_3$): δ 174.4 (C=O), 153.6, 151.1 (CH), 150.0, 142.6, 140.8, 136.8, 129.5 (CH), 129.0 (CH), 124.5 (CH), 123.0, 122.1 (CNO_2), 120.4 (CH), 60.6 (OCH_2), 57.6 ($CHCl_2$), 49.3 (2 NCH_2), 40.6 (CH), 27.6 (2 CH_2), 14.2 (CH_3) ppm. MS: *m*/*z* (%) = 511 [M^+] (10), 494 $[M-OH]^+$ (50), 476 $[M-Cl]^+$

(8), 466 [M-OEt]$^+$ (20), 368 (100), 162 [7-chloroquinolin-4-ylium]$^+$ (80). HRMS (ESI): calcd. for $C_{21}H_{20}Cl_3N_5O_4Na$ [M + Na]$^+$: 534.0473; found: 535.0463.

4-(4-Chlorophenyl)-1-(1-(7-chloroquinolin-4-yl)-5-(dichloromethyl)-4-nitro-1*H*-pyrazol-3-yl) piperidin-4-ol (5f) was synthesized from diene **4f** and 7-chloro-4-hydrazinylquinoline according to the procedure employed for compound **5a** (reflux in methanol 10 h) using purification with column chromatography (petroleum ether/ethyl acetate 2:1). Yield: 40%. M.p. 185–186 °C; yellow solid. IR: $\tilde{\nu}_{max}$ 3281 (OH), 1557, 1493, 1340, 1094, 822, 737 cm^{-1}. ^{1}H NMR (400 MHz, $CDCl_3$): δ 9.11 (d, *J* = 4.6 Hz, 1 H, CH), 8.24 (d, *J* = 1.9 Hz, 1 H, CH), 7.76 (d, *J* = 4.6 Hz, 1 H, CH), 7.60 (s, 1 H, $CHCl_2$), 7.57 (dd, *J* = 9.0, 1.9 Hz, 1 H, CH), 7.47 (d, *J* = 8.6 Hz, 2 H, CH), 7.44 (d, *J* = 9.0 Hz, 1 H, CH), 7.35 (d, *J* = 8.6 Hz, 2 H, CH), 3.66–3.56 (m, 2 H, NCH_2), 3.40 (ddd, *J* = 12.4, 12.4, 2.1 Hz, 2 H, NCH_2), 2.32 (ddd, *J* = 13.0, 13.0, 4.2 Hz, 2 H, CH_2), 1.84 (dd, *J* = 14.1, 1.8 Hz, 2 H, CH_2), 1.70 (br s, 1 H, OH) ppm. ^{13}C NMR (100 MHz, $CDCl_3$): δ 153.7, 151.1 (CH), 150.0, 146.3, 142.7, 140.9, 136.8, 133.2, 129.5 (CH), 129.0 (CH), 128.6 (2 CH), 126.0 (2 CH), 124.5 (CH), 123.1, 122.1 (CNO_2), 120.4 (CH), 71.0 (C-OH), 57.6 ($CHCl_2$), 45.9 (2 NCH_2), 37.9 (2 CH_2) ppm. MS: *m*/*z* (%) = 565 [M$^+$] (7), 548 [M-OH]$^+$ (15), 532 [M-Cl]$^+$ (7), 458 (12), 162 [7-chloroquinolin-4-ylium]$^+$ (35), 139 [4-ClPh-C$^+$=O] (100). HRMS (ESI): calcd. for $C_{24}H_{19}Cl_4N_5O_3Na$ [M + Na]$^+$: 588.0134; found: 588.0143.

7-Chloro-4-(5-(dichloromethyl)-3-(3,4-dihydroisoquinolin-2(1*H*)-yl)-4-nitro-1*H*-pyrazol-1-yl)quinoline (5g) was synthesized from diene **4g** and 7-chloro-4-hydrazinylquinoline according to the procedure employed for compound **5a** (reflux in methanol 7 h) using purification with column chromatography (petroleum ether/ethyl acetate 5:1). Yield: 68%. M.p. 130–131 °C; yellow solid. IR: $\tilde{\nu}_{max}$ 3050, 1555, 1448,

1339, 1177, 817, 736 cm^{-1}. ^{1}H NMR (400 MHz, $CDCl_3$): δ 9.13 (d, J = 4.6 Hz, 1 H, CH), 8.26 (d, J = 2.0 Hz, 1 H, CH), 7.77 (d, J = 4.6 Hz, 1 H, CH), 7.60 (s, 1 H, $CHCl_2$), 7.57 (dd, J = 9.0, 2.0 Hz, 1 H, CH), 7.44 (d, J = 9.0 Hz, 1 H, CH), 7.21–7.14 (m, 3 H, CH), 7.08–7.06 (m, 1 H, CH), 4.50 (s, 2 H, NCH_2), 3.63–3.58 (m, 2 H, NCH_2), 3.12 (s, 2 H, CH_2) ppm. ^{13}C NMR (100 MHz, $CDCl_3$): δ 153.2, 151.1 (CH), 150.1, 142.6, 140.8, 136.8, 134.1, 133.1, 129.5 (CH), 129.0 (CH), 128.9 (CH), 126.6 (CH), 126.4 (CH), 126.1 (CH), 124.5 (CH), 123.1, 121.8 (CNO_2), 120.4 (CH), 57.6 ($CHCl_2$), 50.9 (NCH_2), 48.3 (NCH_2), 28.6 (CH_2) ppm. MS: m/z (%) = 487 [M^+] (5), 470 $[M\text{-}OH]^+$ (100), 452 $[M\text{-}Cl]^+$ (3), 439 $[M\text{-}HNO_2\text{-}H]^+$ (50), 405 $[M\text{-}CHCl_2\text{+}H]^+$ (12), 162 [7-chloroquinolin-4-ylium]$^+$ (23). HRMS (ESI): calcd. for $C_{22}H_{16}Cl_3N_5O_2Na$ $[M + Na]^+$: 510.0262; found: 510.0270.

7-Chloro-4-(5-(dichloromethyl)-3-(1-methyl-3,4-dihydroisoquinolin-2(1*H*)-yl)-4-nitro-1*H*-pyrazol-1-yl)quinoline (5h) was synthesized from diene **4h** and 7-chloro-4-hydrazinylquinoline according to the procedure employed for compound **5a** (reflux in methanol 14 h) using purification with column chromatography (petroleum ether/ethyl acetate 5:1). Yield: 44%. M.p. 143–144 °C; yellow solid. IR: $\tilde{\nu}_{max}$ 2950, 1557, 1487, 1347, 1057, 822, 729 cm^{-1}. ^{1}H NMR (400 MHz, $CDCl_3$): δ 9.11 (d, J = 4.6 Hz, 1 H, CH), 8.25 (d, J = 1.9 Hz, 1 H, CH), 7.74 (d, J = 4.6 Hz, 1 H, CH), 7.54 (dd, J = 8.9, 1.9 Hz, 1 H, CH), 7.53 (s, 1 H, $CHCl_2$), 7.42 (d, J = 8.9 Hz, 1 H, CH), 7.20–7.11 (m, 3 H, CH), 7.10–7.06 (m, 1 H, CH), 4.86 (q, J = 6.6 Hz, 1 H, NCH), 3.80 (dd, J = 13.3, 5.4 Hz, 1 H, NCH_2), 3.61 (ddd, J = 12.7, 12.4, 4.1 Hz, 1 H, NCH_2), 3.41–3.26 (m, 1 H, CH_2), 2.73 (d, J = 16.6 Hz, 1 H, CH_2), 1.62 (d, J = 6.6 Hz, 3 H, CH_3) ppm. ^{13}C NMR (100 MHz, $CDCl_3$): δ 152.3, 151.1 (CH), 150.1, 142.7, 140.5, 138.8, 136.8, 133.5, 129.4 (CH), 129.4 (CH), 129.0 (CH), 126.9 (CH), 126.9 (CH), 126.0 (CH), 124.6 (CH), 123.1, 121.8 (CNO_2), 120.3 (CH), 57.7 ($CHCl_2$), 55.4 (NCH), 41.5 (NCH_2), 27.4 (CH_2), 21.4 (CH_3) ppm. MS: m/z (%) = 501 [M^+] (5), 486 $[M\text{-}Me]^+$ (100), 484 $[M\text{-}OH]^+$ (98), 466 $[M\text{-}Cl]^+$

(4), 383 [M-$CHCl_2$-Cl]$^+$ (8), 368 [M-$CHCl_2$-HNO_2]$^+$ (4), 162 [7-chloroquinolin-4-ylium]$^+$ (17). HRMS (ESI): calcd. for $C_{23}H_{18}Cl_3N_5O_2Na$ [M + Na]$^+$: 524.0418; found: 524.0423.

4-(1-(7-Chloroquinolin-4-yl)-5-(dichloromethyl)-4-nitro-1*H*-pyrazol-3-yl)morpholine (5i) was synthesized from diene **4i** and 7-chloro-4-hydrazinylquinoline according to the procedure employed for compound **5a** (reflux in methanol 4 h) using purification with column chromatography (petroleum ether/ethyl acetate 3:1). Yield: 72%. M.p. 181–182 °C; light brown solid. IR: $\tilde{\nu}_{max}$ 3034, 2828, 1556, 1492, 1346, 1110, 817, 736 cm^{-1}. ^{1}H NMR (400 MHz, $CDCl_3$): δ 9.12 (d, *J* = 4.6 Hz, 1 H, CH), 8.25 (d, *J* = 2.0 Hz, 1 H, CH), 7.74 (d, *J* = 4.6 Hz, 1 H, CH), 7.57 (s, 1 H, $CHCl_2$), 7.56 (dd, *J* = 8.9, 2.0 Hz, 1 H, CH), 7.39 (d, *J* = 8.9 Hz, 1 H, CH), 3.89–3.85 (m, 4 H, 2 OCH_2), 3.33–3.29 (m, 4 H, 2 NCH_2) ppm. ^{13}C NMR (100 MHz, $CDCl_3$): δ 153.3, 151.1 (CH), 150.1, 142.5, 141.0, 136.9, 129.5 (CH), 129.0 (CH), 124.4 (CH), 123.0, 122.2 (CNO_2), 120.4 (CH), 66.4 (OCH_2), 57.5 ($CHCl_2$), 49.9 (NCH_2) ppm. MS: *m*/*z* (%) = 441 [M$^+$] (30), 424 [M-OH]$^+$ (32), 406 [M-Cl]$^+$ (20), 378 [M-OH-NO_2]$^+$ (75), 162 [7-chloroquinolin-4-ylium]$^+$ (100). HRMS (ESI): calcd. for $C_{17}H_{14}Cl_3N_5O_3Na$ [M + Na]$^+$: 464.0054; found: 464.0062.

(4-(1-(7-Chloroquinolin-4-yl)-5-(dichloromethyl)-4-nitro-1*H*-pyrazol-3-yl)piperazin-1-yl)(tetrahydrofuran-2-yl)methanone (5j) was synthesized from diene **4j** and 7-chloro-4-hydrazinylquinoline according to the procedure employed for compound **5a** (reflux in methanol 3 h) using purification with column chromatography (petroleum ether/ethyl acetate 1:1). Yield: 64%. M.p. 203–204 °C; yellow solid. IR: $\tilde{\nu}_{max}$ 2964, 1656, 1553, 1440, 1346, 1235, 1023, 832, 752 cm^{-1}. ^{1}H NMR (400 MHz, $CDCl_3$): δ 9.11 (d, *J* = 4.6 Hz, 1 H, CH), 8.25 (d, *J* = 1.9 Hz, 1 H, CH), 7.73 (d, *J* = 4.6 Hz, 1 H, CH), 7.58 (s, 1 H, $CHCl_2$), 7.56 (dd, *J* = 9.0, 1.9 Hz, 1 H, CH), 7.37 (d, *J* = 9.0 Hz, 1 H, CH), 4.60 (dd, *J* = 7.1, 5.6 Hz, 1 H, OCH), 3.96–3.65 (m, 6 H, 2 NCH_2 + OCH_2), 3.39–

3.21 (m, 4 H, 2 NCH_2), 2.38–2.26 (m, 1 H, CH_2), 2.08–1.83 (m, 3 H, CH_2) ppm. ^{13}C NMR (100 MHz, $CDCl_3$): δ 170.0 (C=O), 153.1, 151.1 (CH), 150.0, 142.3, 141.0, 136.9, 129.5 (CH), 129.0 (CH), 124.3 (CH), 122.9, 122.2 (CNO_2), 120.4 (CH), 75.9 (OCH), 69.0 (OCH_2), 57.5 ($CHCl_2$), 49.9 (NCH_2), 49.6 (NCH_2), 45.0 (NCH_2), 41.5 (NCH_2), 28.2 (CH_2), 25.7 (CH_2) ppm. MS: *m*/*z* (%) = 538 [M^+] (35), 521 $[M\text{-}OH]^+$ (10), 503 $[M\text{-}Cl]^+$ (10), 467 $[M\text{-tetrahydrofuryl}]^+$ (15), 409 $[M\text{-}CHCl_2\text{-}NO_2]^+$ (18), 162 $[\text{7-chloroquinolin-4-ylium}]^+$ (100). HRMS (ESI): calcd. for $C_{22}H_{21}Cl_3N_6O_4Na$ $[M + Na]^+$: 561.0582; found: 561.0593.

7-Chloro-4-(5-(dichloromethyl)-3-(4-(4-fluorophenyl)piperazin-1-yl)-4-nitro-1*H*-pyrazol-1-yl)quinoline (5k) was synthesized from diene **4k** and 7-chloro-4-hydrazinylquinoline according to the procedure employed for compound **5a** (reflux in methanol 14 h) using purification with column chromatography (petroleum ether/ethyl acetate 5:1). Yield: 48%. M.p. 138–139 °C; orange solid. IR: $\tilde{\nu}_{max}$ 2829, 1555, 1506, 1341, 1228, 939, 820, 527 cm^{-1}. ^1H NMR (400 MHz, $CDCl_3$): δ 9.13 (d, *J* = 4.6 Hz, 1 H, CH), 8.26 (d, *J* = 1.9 Hz, 1 H, CH), 7.76 (d, *J* = 4.6 Hz, 1 H, CH), 7.58 (s, 1 H, $CHCl_2$), 7.57 (dd, *J* = 9.0, 2.0 Hz, 1 H, CH), 7.42 (d, *J* = 9.0 Hz, 1 H, CH), 7.03–6.88 (m, 4 H, Ar), 3.52–3.44 (m, 4 H, 2 NCH_2), 3.32–3.24 (m, 4 H, 2 NCH_2) ppm. ^{13}C NMR (100 MHz, $CDCl_3$): δ 157.5 (d, $^1J_{C\text{-}F}$ = 239.6 Hz), 153.2, 151.1 (CH), 150.1, 147.7 (d, $^4J_{C\text{-}F}$ = 2.0 Hz), 142.5, 141.0, 136.8, 129.5 (CH), 129.0 (CH), 124.4 (CH), 123.0, 122.2 (CNO_2), 120.4 (CH), 118.3 (d, $^3J_{C\text{-}F}$ = 7.7 Hz, 2 CH), 115.6 (d, $^2J_{C\text{-}F}$ = 22.1 Hz, 2 CH), 57.6 ($CHCl_2$), 49.9 (2 NCH_2), 49.6 (2 NCH_2) ppm. MS: *m*/*z* (%) = 534 [M^+] (15), 518 $[M\text{-}O]^+$ (2), 499 $[M\text{-}Cl]^+$ (2), 453 $[M\text{-}Cl\text{-}NO_2]^+$ (3), 162 $[\text{7-chloroquinolin-4-ylium}]^+$ (15), 122 (100). HRMS (ESI): calcd. for $C_{23}H_{18}Cl_3FN_6O_2Na$ $[M + Na]^+$: 557.0433; found: 557.0440.

1-(7-Chloroquinol-4-yl)-3-(dichloromethyl)-*N*-(2-fluorobenzyl)-4-nitro-1*H*-pyrazol-5-amine (5l) was synthesized from diene **4l** and 7-chloro-4-hydrazinylquinoline according to the procedure employed for compound **5a** (reflux in methanol 8 h) using purification with column chromatography (petroleum ether/ethyl acetate 5:1). Yield: 45%. M.p. 188–189 °C; light yellow solid. IR: $\tilde{\nu}_{max}$ 3322, 2980, 1612, 1535, 1441, 1343, 1230, 818, 754 cm^{-1}. ^{1}H NMR (400 MHz, $CDCl_3$): δ 9.00 (d, *J* = 4.6 Hz, $^{1}J_{C\text{-}H}$ = 183.6 Hz, 1 H, CH), 8.22 (d, *J* = 2.1 Hz, $^{1}J_{C\text{-}H}$ = 169.7 Hz, 1 H, CH), 7.73 (t, *J* = 6.5 Hz, 1 H, NH), 7.53 (dd, *J* = 9.0, 2.1 Hz, 1 H, CH), 7.48 (d, *J* = 9.0 Hz, 1 H, CH), 7.44 (d, *J* = 4.6 Hz, 1 H, CH), 7.33 (s, 1 H, $CHCl_2$), 7.18 (dddd, *J* = 7.8, 7.8, 5.9, 1.8 Hz, 1 H, CH), 6.96 (ddd, *J* = 7.6, 7.6, 0.8 Hz, 1 H, CH), 6.84–6.79 (m, 2 H, CH), 3.98 (d, *J* = 6.5 Hz, 2 H, NCH_2) ppm. ^{13}C NMR (100 MHz, $CDCl_3$): δ 160.0 (d, $^{1}J_{C\text{-}F}$ = 247.6 Hz), 151.2 (CH), 149.9, 148.1, 147.1, 142.0, 137.2, 130.3 (d, $^{3}J_{C\text{-}F}$ = 8.3 Hz, CH), 129.8 (CH), 129.1 (CH), 128.2 (d, $^{4}J_{C\text{-}F}$ = 3.4 Hz, CH), 124.5 (d, $^{3}J_{C\text{-}F}$ = 3.7 Hz, CH), 123.9 (CH), 122.7 (d, $^{2}J_{C\text{-}F}$ = 14.1 Hz), 122.6, 119.3 (CH), 115.7 (d, $^{2}J_{C\text{-}F}$ = 20.9 Hz, CH), 115.3 (CNO_2), 62.3 ($CHCl_2$), 42.3 (d, $^{3}J_{C\text{-}F}$ = 4.3 Hz, NCH_2) ppm. MS: *m*/*z* (%) = 479 [M^+] (2), 461 $[M\text{-}H\text{-}OH]^+$ (4), 444 $[M\text{-}Cl]^+$ (1), 378 $[M\text{-}CHCl_2\text{-}H_2O]^+$ (2), 162 [7-chloroquinolin-4-ylium]$^+$ (5), 109 [3-fluoro-4-methylenecyclohexa-2,5-dien-1-ylium]$^+$ (100). HRMS (ESI): calcd. for $C_{20}H_{13}Cl_3FN_5O_2Na$ $[M + Na]^+$: 502.0011; found: 502.0025.

1-(7-Chloroquinol-4-yl)-3-(dichloromethyl)-*N*-(4-fluorophenyl)-4-nitro-1*H*-pyrazol-5-amine (5m) was synthesized from diene **4m** and 7-chloro-4-hydrazinylquinoline according to the procedure employed for compound **5a** (reflux in methanol 7 h) using purification with column chromatography (petroleum ether/ethyl acetate 2:1). Yield: 35%. M.p. 153–154 °C; yellow solid. IR: $\tilde{\nu}_{max}$ 3326, 1586, 1503, 1362, 1209, 1072, 822, 743 cm^{-1}. ^{1}H NMR (400 MHz, $CDCl_3$): δ 8.80 (br s, 1 H, NH),

8.70 (d, J = 4.6 Hz, 1 H, CH), 8.07 (d, J = 2.0 Hz, 1 H, CH), 7.68 (d, J = 9.0 Hz, 1 H, CH), 7.61 (dd, J = 9.0, 2.0 Hz, 1 H, CH), 7.37 (s, 1 H, $CHCl_2$), 7.14 (d, J = 4.6 Hz, 1 H, CH), 6.64 (dd, J = 8.7, 4.6 Hz, 2 H, CH), 6.48 (dd, J = 8.7, 8.1 Hz, 2 H, CH) ppm. ^{13}C NMR (100 MHz, $CDCl_3$): δ 160.8 (d, $^1J_{C\text{-}F}$ = 249.9 Hz), 150.7 (CH), 149.6, 147.1, 145.8, 141.4, 136.8, 130.9 (d, $^4J_{C\text{-}F}$ = 3.7 Hz), 129.5 (CH), 129.0 (CH), 126.2 (d, $^3J_{C\text{-}F}$ = 8.6 Hz, 2 CH), 123.9 (CH), 121.4, 118.8 (CH), 116.1 (CNO_2), 115.7 (d, $^2J_{C\text{-}F}$ = 23.1 Hz, 2 CH), 62.2 ($CHCl_2$) ppm. MS: *m*/*z* (%) = 465 [M^+] (100), 448 $[M\text{-}OH]^+$ (10), 430 $[M\text{-}Cl]^+$ (8), 394 $[M\text{-}Cl\text{-}HCl]^+$ (17), 193 (80), 162 [7-chloroquinolin-4-ylium] (57). HRMS (ESI): calcd. for $C_{19}H_{11}Cl_3FN_5O_2Na$ $[M + Na]^+$: 487,9855; found: 487.9852.

1-(7-Chloroquinol-4-yl)-3-(dichloromethyl)-*N*-(4-ethoxyphenyl)-4-nitro-1*H*-pyrazol-5-amine (5n) was synthesized from diene **4n** and 7-chloro-4-hydrazinylquinoline according to the procedure employed for compound **5a** (stirring in ethanol for 8 h) using purification with column chromatography (petroleum ether/ethyl acetate 5:1). Yield: 9%. M.p. 146–147 °C; yellow solid. IR: $\tilde{\nu}_{max}$ 2980, 1594, 1447, 1345, 1243, 1042, 818, 744 cm^{-1}. ^1H NMR (400 MHz, $CDCl_3$): δ 8.81 (br s, 1 H, NH), 8.67 (d, J = 4.6 Hz, 1 H, CH), 8.04 (d, J = 2.0 Hz, 1 H, CH), 7.64 (d, J = 9.0 Hz, 1 H, CH), 7.59 (dd, J = 9.0, 2.0 Hz, 1 H, CH), 7.38 (s, 1 H, $CHCl_2$), 7.10 (d, J = 4.6 Hz, 1 H, CH), 6.54 (d, J = 8.7 Hz, 2 H, CH), 6.23 (d, J = 8.7 Hz, 2 H, CH), 3.74 (q, J = 7.1 Hz, 2 H, OCH_2), 1.29 (t, J = 7.1 Hz, 3 H, Me) ppm. ^{13}C NMR (100 MHz, $CDCl_3$): δ 157.9 (C-O), 150.6 (CH), 149.5, 147.0, 146.6, 141.6, 136.6, 129.3 (CH), 128.7 (CH), 127.1, 126.4 (2 CH), 124.1 (CH), 121.9, 119.2 (CH), 115.5 (CNO_2), 114.3 (2 CH), 63.7 (OCH_2), 62.3 ($CHCl_2$), 14.5 (Me) ppm. MS: *m*/*z* (%) = 491 [M^+] (100), 476 [M^+-Me] (8), 446 [M^+-OEt] (8), 346 (20), 162 [7-chloroquinolin-4-ylium]$^+$ (45). HRMS (ESI): calcd. for $C_{21}H_{16}Cl_3N_5O_3Na$ $[M + Na]^+$: 514,0211; found: 514.0220.

***N*-(1-(7-Chloroquinol-4-yl)-3-(dichloromethyl)-4-nitro-1*H*-pyrazol-5-yl)-5-methylisoxazol-3-amine (5o)** was synthesized from diene **4o** and 7-chloro-4-hydrazinylquinoline according to the procedure employed for compound **5a** (stirring in methanol at 45–50 °C for 6 h) using purification with column chromatography (petroleum ether/ethyl acetate 5:1). Yield: 5%. M.p. 216–217 °C; yellow solid. IR: $\tilde{\nu}_{max}$ 3371, 1620, 1592, 1470, 1347, 1072, 870, 752 cm^{-1}. ^1H NMR (400 MHz, $CDCl_3$): δ 9.15 (d, J = 4.6 Hz, 1 H, CH), 8.77 (br s, 1 H, NH), 8.29 (d, J = 2.0 Hz, 1 H, CH), 7.77 (d, J = 4.6 Hz, 1 H, CH), 7.61 (s, 1 H, $CHCl_2$), 7.59 (dd, J = 9.0, 2.0 Hz, 1 H, CH), 7.47 (d, J = 9.0 Hz, 1 H, CH), 6.40 (q, J = 0.7 Hz, 1 H, CH), 2.32 (d, J = 0.7 Hz, 3 H, Me) ppm. A ^{13}C NMR spectrum could not be be measured due to the to low yield. MS: m/z (%) = 452 $[M^+]$ (100), 435 $[M\text{-}OH]^+$ (5), 417 $[M\text{-}Cl]^+$ (20), 371 $[M\text{-}Cl\text{-}NO_2]^+$ (25), 369 $[M\text{-}CHCl_2]^+$ (25), 323 $[M\text{-}CHCl_2\text{-}NO_2]^+$ (23), 162 [7-chloroquinolin-4-ylium]$^+$ (95). HRMS (ESI): calcd. for $C_{17}H_{11}Cl_3N_6O_3Na$ $[M + Na]^+$: 474,9850; found: 474.9848.

3-Methyl-2-(2,3,3-trichloro-1-nitroallylidene)oxazolidine (6) was synthesized from nitrodiene **1** (271 mg, 1.00 mmol) and 2-(methylamino)ethanol (233 mg, 3.1 mmol) in methanol at −20 °C according to the literature [5] (yield 159 mg, 58%). Synthesis of **6** from benzotriazole **2c**: At rt, a suspension of 4.37 g (10.0 mmol) bis(benzotriazolyl) compound **2c** in 50 mL of MeOH was treated with a solution of 0.826 g (11.0 mmol) 2-(methylamino)ethanol in 5 mL MeOH. The resulting mixture was kept at rt for 3 h. After cooling to 10 °C, a solution of 5 mL conc. hydrochloric acid in 250 mL of cold water was added under stirring. After 1 h, the precipitate was isolated and washed with HCl (5%, 30 mL), cold water (2 × 50 mL), and cold methanol (5 mL). Drying under reduced pressure afforded the oxazolidine **6** (2.08 g, 76%). M.p. 140–141 °C; colorless solid. IR: $\tilde{\nu}_{max}$ 2983, 1599, 1576, 1506, 1459, 1314, 1279, 1163, 1081, 917, 826, 708 cm^{-1}. ^1H NMR (200 MHz, $CDCl_3$): δ 4.69 (t, J = 8.7 Hz, 2 H, OCH_2, $^1J_{C\text{-}H}$ = 156.4 Hz), 4.29–

4.04 (m, 2 H, NCH_2), 3.19 (s, $^1J_{C\text{-}H}$ = 141.8 Hz, 3 H, Me) ppm. ^{13}C NMR (50 MHz, $CDCl_3$): δ 164.4 (OCN), 125.0, 124.2 (CCl_2=CCl), 104.9 (CNO_2), 67.3 (OCH_2), 52.4 (NCH_2), 36.5 (Me) ppm. MS: *m*/*z* (%) = 272 [M^+] (2), 237 $[M\text{-}Cl]^+$ (11), 191 $[M\text{-}Cl\text{-}NO_2]^+$ (17), 143 $[M\text{-}C_2Cl_3]^+$ (40), 127 $[M\text{-}C_2Cl_3\text{-}O]^+$ (100). HRMS (ESI): calcd. for $C_7H_7Cl_3N_2O_3Na$ $[M + Na]^+$: 294.9414; found: 294.9419

2-((1-(7-Chloroquinol-4-yl)-5-(dichloromethyl)-4-nitro-1*H*-pyrazol-3-yl)(methyl)-amino)ethan-1-ol (7). At rt, 7-chloro-4-hydrazinylquinoline (445 mg, 2.30 mmol) was added to a solution of oxazolidine **6** (274 mg, 1.00 mmol) in 10 mL toluene portionwise within 5 min. The resulting mixture was stirred for 30 h at 90–95 °C. After removing the solvent *in vacuo*, water (50 mL), chloroform (20 mL), and 10 drops of conc. hydrochloric acid were added under stirring. After extraction with chloroform (3 × 20 mL), treatment of the combined organic layers twice with water (20 mL each portion), and drying with anhydr. calcium chloride the obtained product was purified by flash column chromatography (first with petroleum ether/ethyl acetate 5:1 to remove the impurities, then 1:1) and finally dried *in vacuo* to yield pyrazole **7** (99 mg, 23%) as yellow solid. M.p. 146–147 °C. IR: $\tilde{\nu}_{max}$ 3303, 2999, 1552, 1482, 1343, 1091, 823, 732 cm^{-1}. ^{1}H NMR (400 MHz, $CDCl_3$): δ 9.09 (d, *J* = 4.6 Hz, 1 H, CH, $^1J_{C\text{-}H}$ = 183.6 Hz), 8.23 (d, *J* = 2.0 Hz, 1 H, CH), 7.73 (d, *J* = 4.6 Hz, 1 H, CH), 7.56 (dd, *J* = 9.0, 2.0 Hz, 1 H, CH), 7.49 (s, 1 H, $CHCl_2$), 7.42 (d, *J* = 9.0 Hz, 1 H, CH), 3.88 (t, *J* = 5.4 Hz, 2 H, OCH_2), 3.48 (t, *J* = 5.4 Hz, 2 H, NCH_2), 3.01 (s, 3 H, Me) ppm. ^{13}C NMR (100 MHz, $CDCl_3$): δ 153.7, 151.1 (CH), 149.9, 142.5, 140.5, 136.8, 129.5 (CH), 128.9 (CH), 124.4 (CH), 123.0, 121.7 (CNO_2), 120.3 (CH), 59.6 (OCH_2), 57.6 ($CHCl_2$), 55.4 (OCH_2), 39.3 (Me) ppm. MS: *m*/*z* (%) = 429 [M^+] (17), 414 $[M\text{-}Me]^+$ (2), 398 $[M\text{-}CH_2OH]^+$ (100), 384 $[M\text{-}CH_2CH_2OH]^+$ (4), 162 $[\text{7-chloroquinolin-4-ylium}]^+$ (28). HRMS (ESI): calcd. for $C_{16}H_{14}Cl_3N_5O_3Na$ $[M + Na]^+$: 452,0054; found: 452.0060.

7-Chloro-4-(5-(dichloromethyl)-4-nitro-3-(propylthio)-1*H*-pyrazol-1-yl)quinoline (9a). At 0 °C, a solution of 7-chloro-4-hydrazinylquinoline (213 mg, 1.10 mmol) and triethylamine (111 mg, 1.10 mmol) in 5 mL dry dichloromethane (DCM) was added to a solution of thiodiene **8a** (311 mg, 1.00 mmol) in 10 mL dry DCM within 5 min. The resulting mixture was stirred for 2 h at 0 °C, then for 20 h at rt. After cooling to 0 °C, water (50 mL) and 5 drops of conc. hydrochloric acid were added under stirring. After extraction with DCM (3 × 30 mL), treatment of the combined organic layers twice with water (30 mL each portion), and drying with anhydr. calcium chloride the obtained product was purified by flash column chromatography (petroleum ether/ethyl acetate 5:1) and finally dried *in vacuo* to yield pyrazole **9a** (164 mg, 38%) as a yellow solid. M.p. 150–151 °C. IR: $\tilde{\nu}_{max}$ 2966, 1596, 1489, 1333, 1126, 826, 747 cm^{-1}. ^1H NMR (400 MHz, $CDCl_3$): δ 9.14 (d, *J* = 4.6 Hz, 1 H, CH; $^1J_{C\text{-}H}$ = 183.8 Hz), 8.27 (d, *J* = 2.0 Hz, 1 H, CH; $^1J_{C\text{-}H}$ = 170.0 Hz), 7.76 (d, *J* = 4.6 Hz, 1 H, CH), 7.67 (s, 1 H, $CHCl_2$; $^1J_{C\text{-}H}$ = 184.3 Hz), 7.58 (dd, *J* = 9.0, 2.0 Hz, 1 H, CH), 7.37 (d, *J* = 9.0 Hz, 1 H, CH), 3.05 (t, *J* = 7.3 Hz, $^1J_{C\text{-}H}$ = 141.7 Hz, 2 H, SCH_2), 1.79–1.70 (m, $^1J_{C\text{-}H}$ = 128.7 Hz, 2 H, CH_2), 1.00 (d, *J* = 7.3 Hz, $^1J_{C\text{-}H}$ = 125.9 Hz, 3 H, Me) ppm. ^{13}C NMR (100 MHz, $CDCl_3$): δ 151.1 (CH), 150.0, 149.5, 142.5, 140.6, 136.9, 129.6 (CH), 129.0 (CH), 128.5 (CNO_2), 124.3 (CH), 123.0, 120.4 (CH), 57.0 ($CHCl_2$), 32.4 (SCH_2), 22.0 (CH_2), 13.4 (Me) ppm. MS: *m*/*z* (%) = 430 [M^+] (27), 415 $[M\text{-}CH_3]^+$ (2), 413 $[M\text{-}OH]^+$ (2), 388 $[M\text{-}Pr\text{+}H]^+$ (4), 337 $[M\text{-}Me\text{-}Cl\text{-}NO_2]^+$ (48), 162 [7-chloroquinolin-4-ylium]$^+$ (100). HRMS (ESI): calcd. for $C_{16}H_{13}Cl_3N_4O_2SNa$ $[M + Na]^+$: 452.9717; found: 452.9723.

4-(3-(Benzylthio)-5-(dichloromethyl)-4-nitro-1*H*-pyrazol-1-yl)-7-chloroquinoline (9b) was synthesized from thiodiene **8b** and 7-chloro-4-hydrazinylquinoline according to the procedure employed for compound **9a** (stirring in dry DCM at rt for 40 h) using

purification with column chromatographie (petroleum ether/ethyl acetate 5:1). Yield: 69%. M.p. 165–166 °C; light yellow solid. IR: $\tilde{\nu}_{max}$ 3038, 1598, 1490, 1334, 1074, 822, 748 cm^{-1}. ^{1}H NMR (400 MHz, $CDCl_3$): δ 9.16 (d, *J* = 4.6 Hz, 1 H, CH), 8.28 (d, *J* = 2.0 Hz, 1 H, CH), 7.76 (d, *J* = 4.6 Hz, 1 H, CH), 7.66 (s, 1 H, $CHCl_2$), 7.52 (dd, *J* = 9.0, 2.0 Hz, 1 H, CH), 7.34–7.31 (m, 2 H, CH), 7.29–7.27 (m, 3 H, CH), 7.24 (d, *J* = 9.0 Hz, 1 H, CH), 4.28 (s, 2 H, SCH_2) ppm. ^{13}C NMR (100 MHz, $CDCl_3$): δ 151.1 (CH), 150.0, 148.7, 142.5, 140.7, 136.9, 136.1, 129.6 (CH), 129.2 (CH), 128.9 (CH), 128.6 (CH), 127.6 (CH), 124.4 (CH), 122.9, 121.5 (CNO_2), 120.3 (CH), 56.9 ($CHCl_2$), 34.8 (SCH_2) ppm. MS: *m*/*z* (%) = 478 [M$^+$] (0.3), 461 [M-OH]$^+$ (0.2), 443 [M-Cl]$^+$ (0.2), 372 (5), 162 [7-chloroquinolin-4-ylium]$^+$ (5), 91 (100). HRMS (ESI): calcd. for $C_{20}H_{13}Cl_3N_4O_2SNa$ [M + Na]$^+$: 500.9717; found: 500.9723.

1-(7-Chloroquinolin-4-yl)-5-(dichloromethyl)-3-(methoxycarbonylmethylsulfanyl)-4-nitro-1*H*-pyrazole (9c) was synthesized from thiodiene **8c** and 7-chloro-4-hydrazinylquinoline according to the procedure employed for compound **9a**. Yield: 28%. M.p. 195–197 °C; light brown solid. IR: $\tilde{\nu}_{max}$ 3021, 1733 (C=O), 1609, 1495, 1338, 1307, 858, 752 cm^{-1}. ^{1}H NMR (400 MHz, DMSO-d_6): δ 9.22 (d, *J* = 4.5 Hz, 1 H, CH), 8.32 (d, *J* = 1.8 Hz, 1 H, CH), 7.94 (d, *J* = 4.5 Hz, 1 H, CH), 7.79 (dd, *J* = 9.0, 1.8 Hz, 1 H, CH), 7.72 (s, 1 H, $CHCl_2$), 7.48 (d, *J* = 9.0 Hz, 1 H, CH), 4.02 (s, 2 H, SCH_2), 3.49 (s, 3 H, CH_3) ppm. ^{13}C NMR (100 MHz, DMSO-d_6): δ 168.6 (C=O), 152.3 (CH), 149.3, 147.5, 141.3, 141.0, 135.6, 129.2 (CH), 129.1(C-NO_2), 128.2 (CH), 124.9 (CH), 122.4, 120.8 (CH), 58.0 ($CHCl_2$), 52.3 (OCH_3), 32.2 (SCH_2) ppm. MS: *m*/*z* (%) = 460 [M$^+$] (10), 444 [M-O]$^+$ (1), 425 [M-Cl]$^+$ (2), 295 (10), 162 [7-chloroquinolin-4-ylium]$^+$ (5), 97 (100). HRMS (ESI): calcd. for $C_{16}H_{11}Cl_3N_4O_4SNa$ [M + Na]$^+$: 482.9459; found: 482.9465.

1-(7-Chloroquinolin-4-yl)-5-(dichloromethyl)-3-(ethoxycarbonylmethylsulfanyl)-4-nitro-1*H*-pyrazole (9d) was synthesized from thiodiene **8d** and 7-chloro-4-hydrazinylquinoline according to the procedure employed for compound **9a**. Yield: 63%. M.p. 175–176 °C; light brown solid. IR: $\tilde{\nu}_{max}$ 3022, 2996, 1726 (C=O), 1612, 1489, 1335, 1014, 846, 751 cm^{-1}. ^1H NMR (400 MHz, DMSO-d_6): δ 9.22 (d, *J* = 4.5 Hz, 1 H, CH), 8.32 (d, *J* = 2.0 Hz, 1 H, CH), 7.94 (d, *J* = 4.6 Hz, 1 H, CH), 7.79 (dd, *J* = 9.0, 2.0 Hz, 1 H, CH), 7.73 (s, 1 H, $CHCl_2$), 7.50 (d, *J* = 9.0 Hz, 1 H, CH), 4.00 (s, 2 H, SCH_2), 3.95 (q, *J* = 7.1 Hz, 2 H, OCH_2), 0.85 (t, *J* = 7.1 Hz, 3 H, CH_3) ppm. ^{13}C NMR (100 MHz, DMSO-d_6): δ 168.1 (C=O), 152.3 (CH), 149.2, 147.5, 141.4, 140.9, 135.6, 129.1 (CH), 129.0 (C-NO_2), 128.1 (CH), 124.9 (CH), 122.4, 120.7 (CH), 61.0 (OCH_2), 58.0 ($CHCl_2$), 52.3 (OCH_3), 32.5 (SCH_2), 13.6 (Me) ppm. MS: *m*/*z* (%) = 474 [M^+] (6), 458 $[M-O]^+$ (0.4), 439 $[M-Cl]^+$ (12), 162 [7-chloroquinolin-4-ylium]$^+$ (44), 97 (100). HRMS (ESI): calcd. for $C_{17}H_{13}Cl_3N_4O_4SNa$ $[M + Na]^+$: 496.9621; found: 496.9623.

7-Chloro-4-(3-((4-chlorophenyl)thio)-5-(dichloromethyl)-4-nitro-1*H*-pyrazol-1-yl)quinoline (9e) was synthesized from thiodiene **8e** and 7-chloro-4-hydrazinylquinoline according to the procedure employed for compound **9a**. Yield: 63%. M.p. 159–160 °C; light yellow solid. IR: $\tilde{\nu}_{max}$ 2953, 1546, 1501, 1339, 1094, 820, 755 cm^{-1}. ^1H NMR (400 MHz,): δ 9.08 (d, *J* = 4.6 Hz, 1 H, CH), 8.22 (d, *J* = 2.0 Hz, 1 H, CH), 7.67 (d, *J* = 4.6 Hz, 1 H, CH), 7.63 (s, 1 H, $CHCl_2$), 7.54 (dd, *J* = 9.0, 2.0 Hz, 1 H, CH), 7.52 (d, *J* = 8.5 Hz, 2 H, CH), 7.28 (d, *J* = 8.5 Hz, 2 H, CH), 7.25 (d, *J* = 9.0 Hz, 1 H, CH) ppm. ^{13}C NMR (100 MHz, $CDCl_3$): δ 151.0 (CH), 149.9, 148.6, 142.1, 140.7, 136.9, 136.1 (2 CH), 130.0, 129.6 (2 CH), 129.5 (CH), 129.0 (CH), 128.1 (C-NO_2), 125.6, 124.0 (CH), 122.9, 120.2 (CH), 56.8 ($CHCl_2$) ppm. MS: *m*/*z* (%) = 498 [M^+] (35), 463 $[M-Cl]^+$ (2), 214 (25), 162 [7-chloroquinolin-4-ylium]$^+$ (40), 159 (100). HRMS (ESI): calcd. for $C_{19}H_{10}Cl_4N_4O_2SNa$ $[M + Na]^+$: 520.9171; found: 520.9171.

1-(7-Chloroquinolin-4-yl)-5-(dichloromethyl)-3-(ethoxycarbonylmethylsulfinyl)-4-nitro-1*H*-pyrazole (**10d**). To a solution of 400 mg (0.84 mmol) of pyrazole **9d** in 30 mL of chloroform was added at rt *m*-chloroperbenzoic acid (0.93 mmol, 207 mg). After stirring at rt for 1 d, further 50 mL of chloroform, 5 mL saturated $NaHCO_3$ solution, and 50 mL water were added to the reaction mixture under stirring. The organic phase was dried with anhydr. sodium sulfate and purified by column chromatography (petroleum ether/ethyl acetate 5:1). After removal of the solvent, 176 mg (0.36 mmol, 43%) of pyrazole **10d** remained as a light brown solid. M.p. 160–161 °C. IR: $\tilde{\nu}_{max}$ 2924, 1728 (C=O), 1613, 1514, 1332, 1259, 1074, 845, 749 cm^{-1}. ^1H NMR (400 MHz, $CDCl_3$): δ 9.15 (d, J = 4.5 Hz, 1 H, CH), 8.27 (d, J = 1.9 Hz, 1 H, CH), 7.75 (d, J = 4.5 Hz, 1 H, CH), 7.60 (dd, J = 8.8, 1.9 Hz, 1 H, CH), 7.57 (s, 1 H, $CHCl_2$), 7.38 (d, J = 8.8 Hz, 1 H, CH), 4.37 (d, J = 13.8 Hz, 1 H, SCH_2), 4.22 (dq, J = 7.2, 3.5 Hz, 2 H, OCH_2), 4.14 (d, J = 13.8 Hz, 1 H, SCH_2), 1.26 (t, J = 7.2 Hz, 1 H, CH_3) ppm. ^{13}C NMR (100 MHz, $CDCl_3$): δ 164.4 (C=O), 151.4, 150.9 (CH), 150.0, 141.9, 141.7, 137.3, 129.9 (CH), 129.1 (CH), 128.8 ($C-NO_2$), 124.2 (CH), 122.8, 120.3 (CH), 62.6 (OCH_2), 56.3 (SCH_2), 56.2 ($CHCl_2$), 14.1 (CH_3) ppm. MS: m/z (%) = 490 [M^+] (12), 445 $[M-OEt]^+$ (3), 403 $[M-CH_2CO_2Et]^+$ (7), 373 (45), 236 (30), 162 $[7\text{-chloroquinolin-4-ylium}]^+$ (100). HRMS (ESI): calcd. for $C_{17}H_{13}Cl_3N_4O_5SNa$ $[M + H]^+$: 512.9564; found: 496.9559.

1-(7-Chloroquinolin-4-yl)-1*H*-pyrazole (**11**). In a 25 mL round-bottomed flask with a reflux condenser were placed 500 mg (2.58 mmol) of 7-chloro-4-hydrazinylquinoline, 7.5 mL of water, and 7.5 mL of ethanol. At rt, 0.2 mL conc. hydrochloric acid and 424 mg (2.58 mmol) 1,1,3,3-tetramethoxypropane were added dropwise under stirring. The mixture was stirred at 90 °C for 8 h and then at rt for 1 d. After transferring the mixture into a separatory funnel and quenching with 50 mL ice water, the mixture was extracted

three times with 50 mL of chloroform. The combined organic layers were washed with water and dried with anhydr. sodium sulfate. After removal of the chloroform and drying in vacuum, 200 mg (0.87 mmol, 34%) of 1-(7-chloroquinol-4-yl)-1*H*-pyrazole (**11**) remained as a colorless solid. M.p. 135–136 °C (Lit. [6] mp 110 °C). IR: $\tilde{\nu}_{max}$ 3102, 1585, 1524, 1440, 1395, 1099, 1044, 930, 754 cm^{-1}. ^{1}H NMR (400 MHz, DMSO-d_6): δ 9.03 (d, *J* = 4.8 Hz, 1 H, CH), 8.49–8.45 (m, 2 H, CH), 8.18 (d, *J* = 2.3 Hz, 1 H, CH), 8.00 (d, *J* = 1.5 Hz, 1 H, CH), 7.74–7.67 (m, 2 H, CH), 6.71 (dd, *J* = 2.3, 1.5 Hz, 1 H, CH) ppm. ^{13}C NMR (100 MHz, DMSO-d_6): δ 152.3 (CH), 149.7, 143.3, 142.6 (CH), 134.8, 132.3 (CH), 127.92 (CH), 127.91 (CH), 126.8 (CH), 120.2, 115.3 (CH), 108.3 (CH) ppm. MS: *m/z* (%) = 229 [M^+] (100), 194 $[M-Cl]^+$ (60), 162 [7-chloroquinol-4-ylium]$^+$ (12).

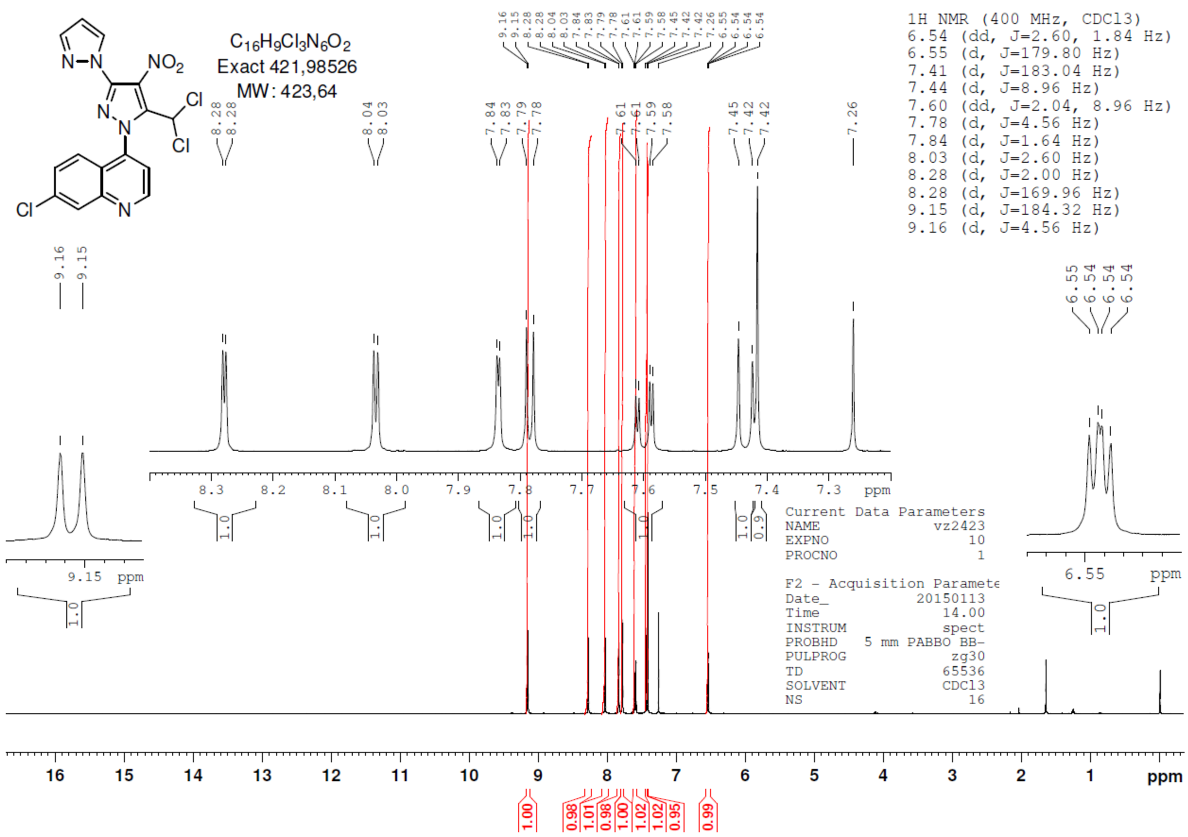

Figure S1. 400 MHz ^{1}H NMR spectrum in $CDCl_3$ for **3a**.

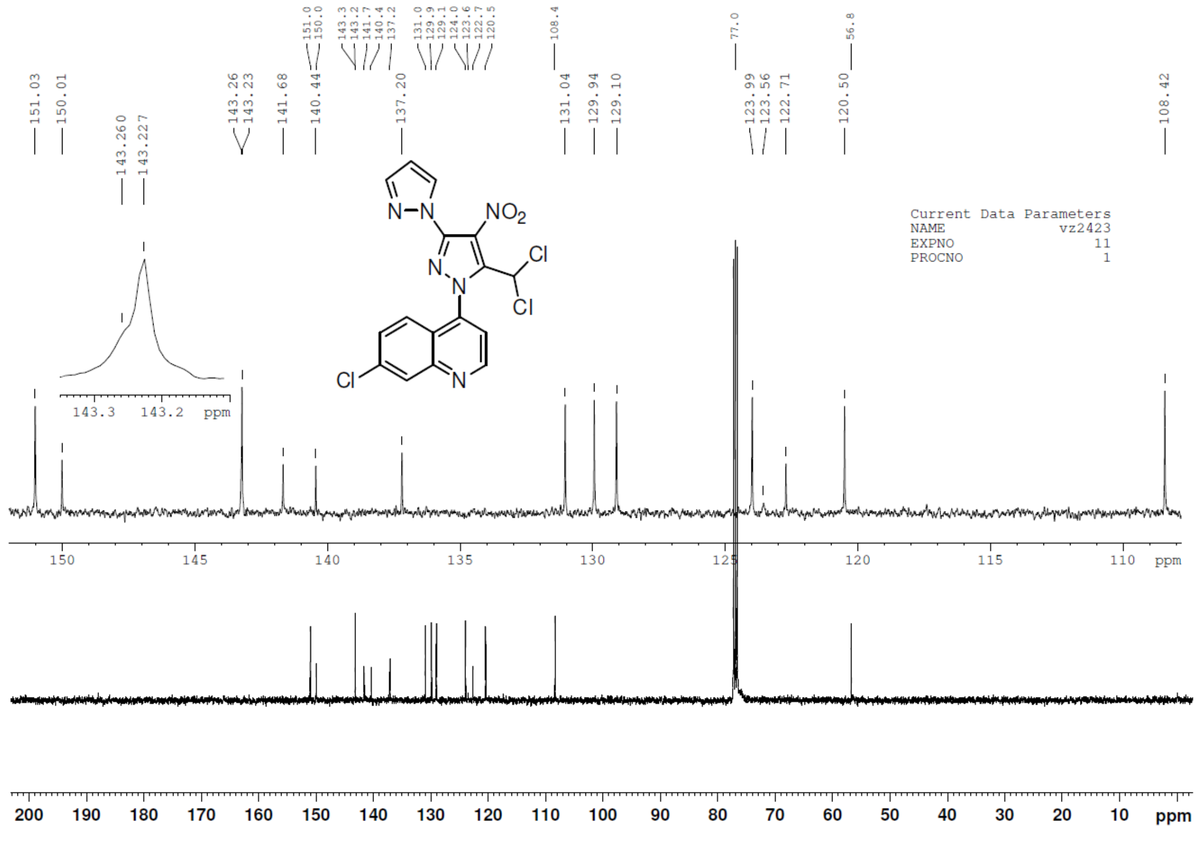

Figure S2. 100 MHz ^{13}C NMR spectrum in $CDCl_3$ for **3a**.

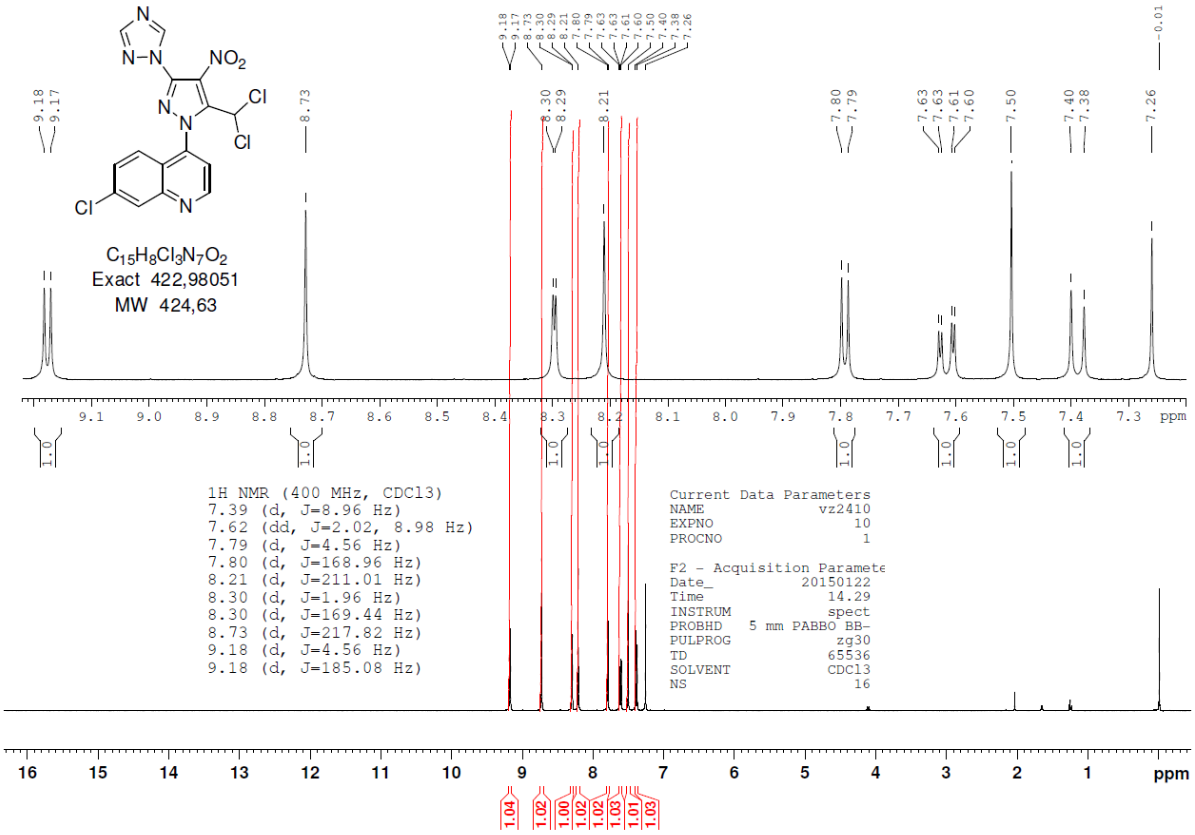

Figure S3. 400 MHz ^{1}H NMR spectrum in $CDCl_3$ for **3b**.

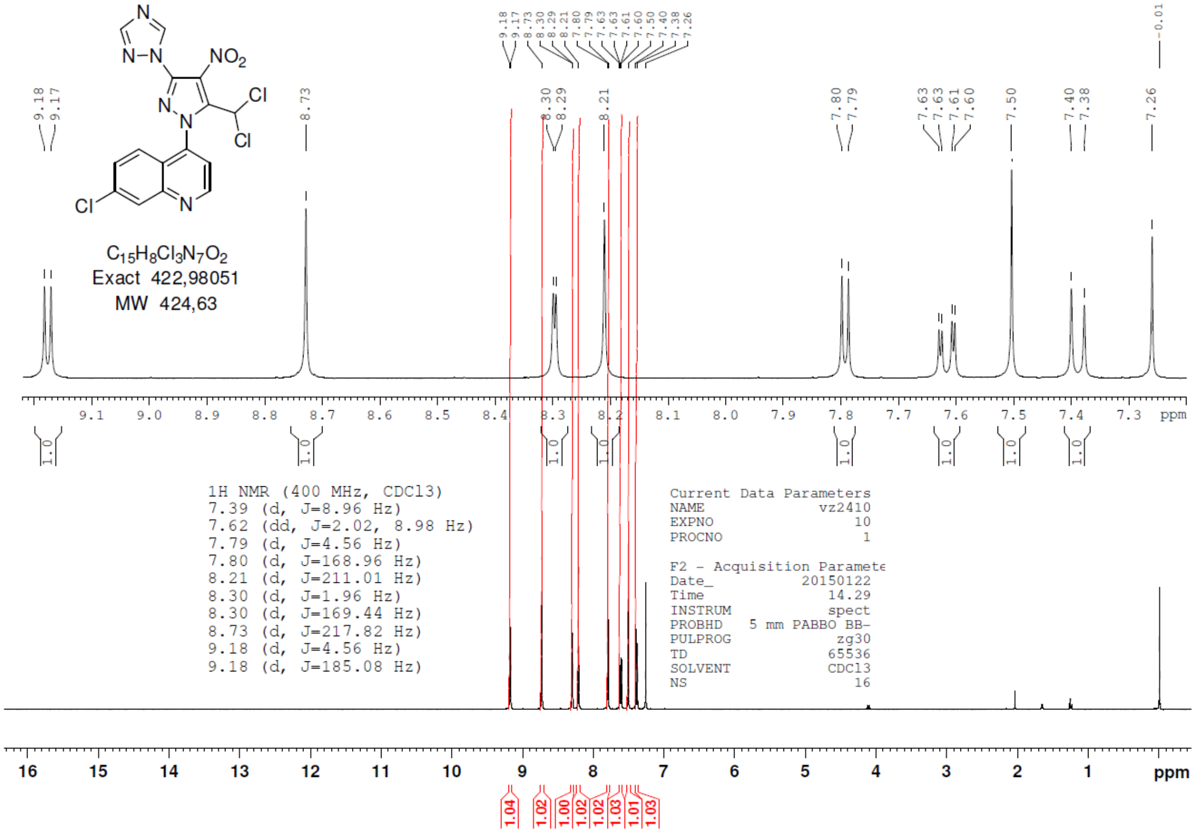

Figure S4. 100 MHz ^{13}C NMR spectrum in $CDCl_3$ for **3b**.

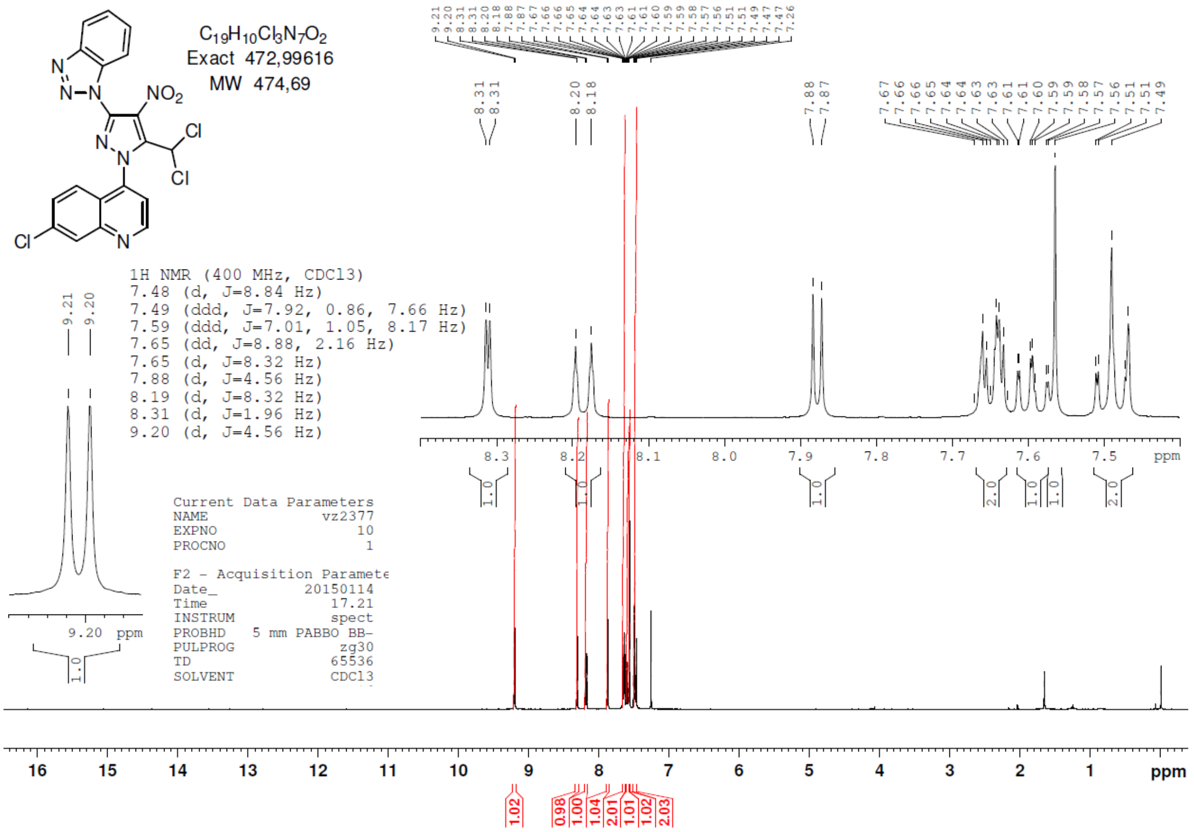

Figure S5. 400 MHz ^{1}H NMR spectrum in $CDCl_3$ for **3c**.

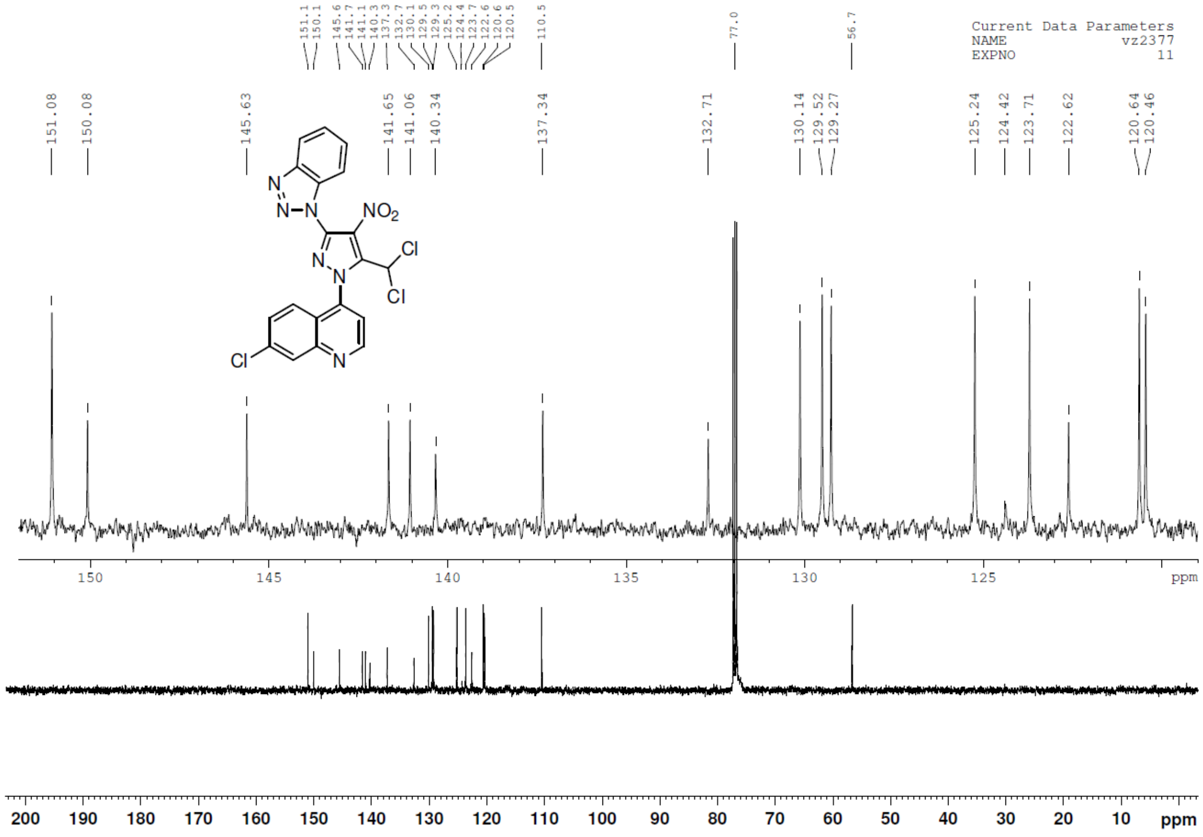

Figure S6. 100 MHz ^{13}C NMR spectrum in $CDCl_3$ for **3c**.

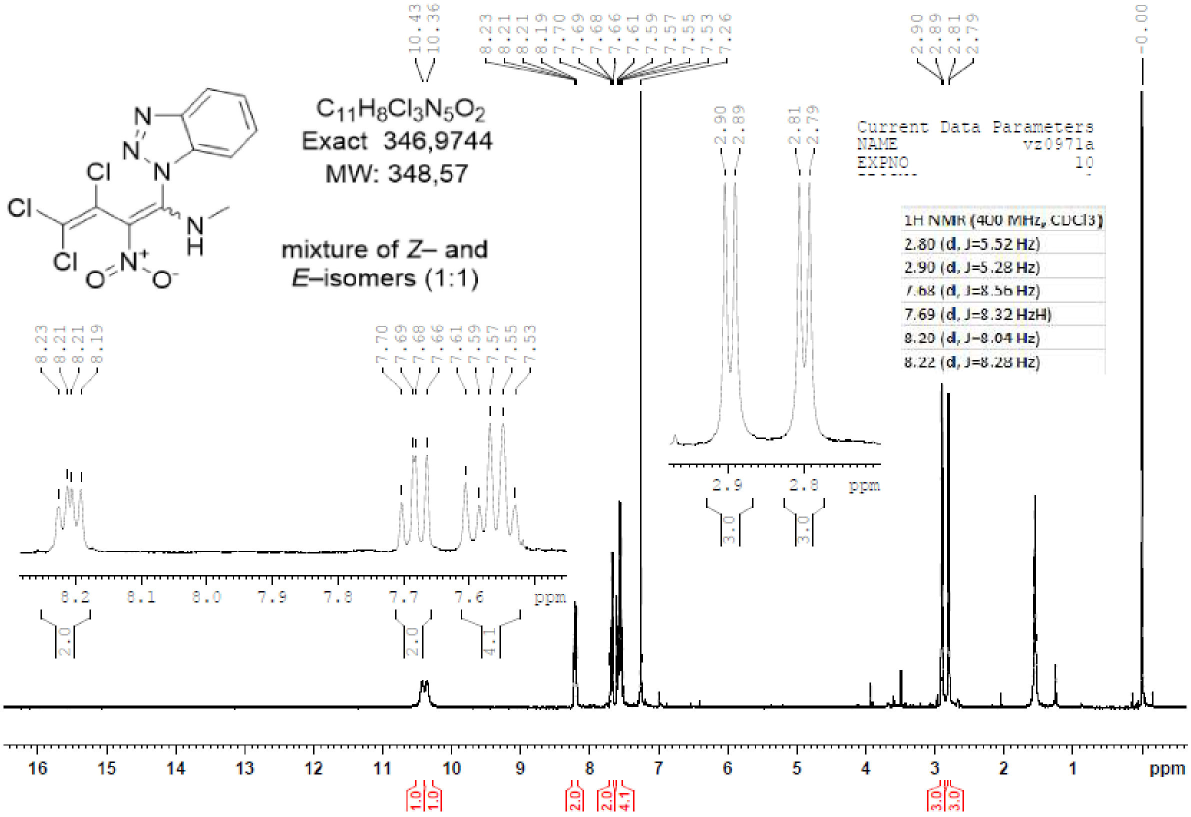

Figure S7. 400 MHz ^{1}H NMR spectrum in $CDCl_3$ for **4a**.

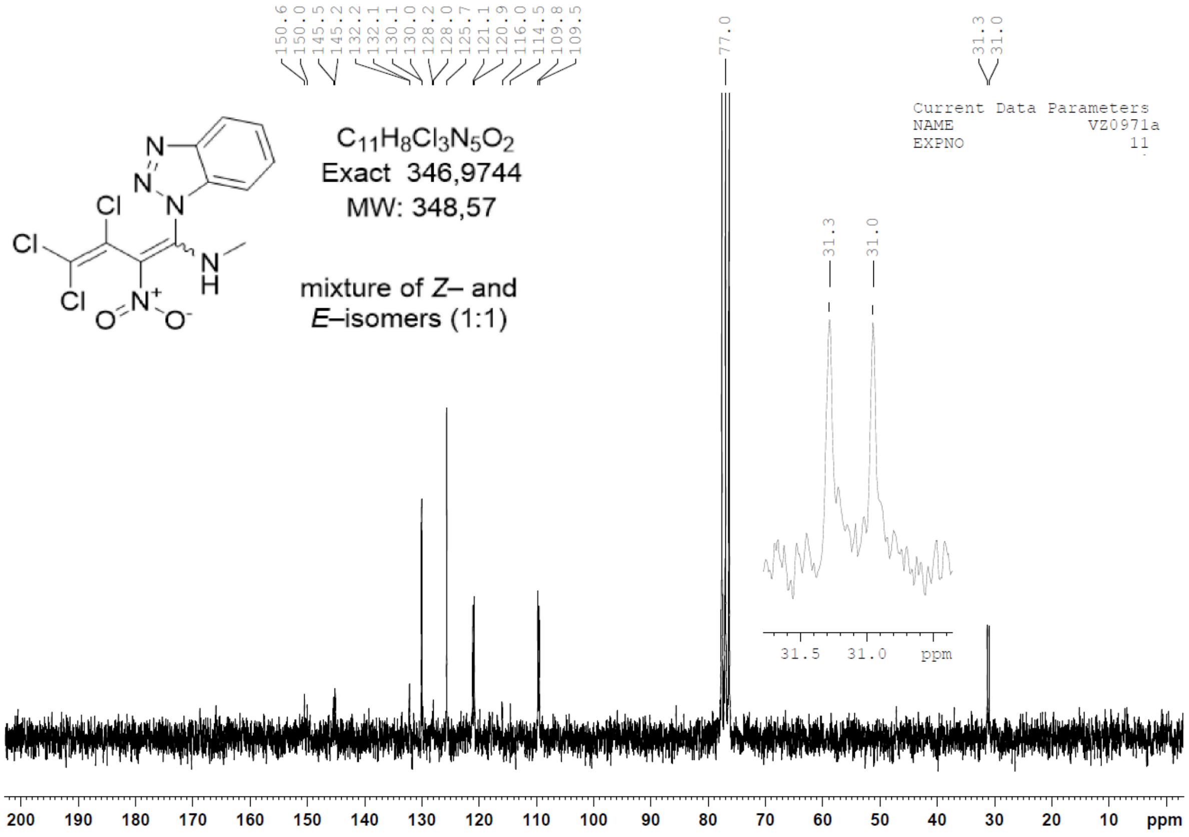

Figure S8. 100 MHz ^{13}C NMR spectrum in $CDCl_3$ for **4a**.

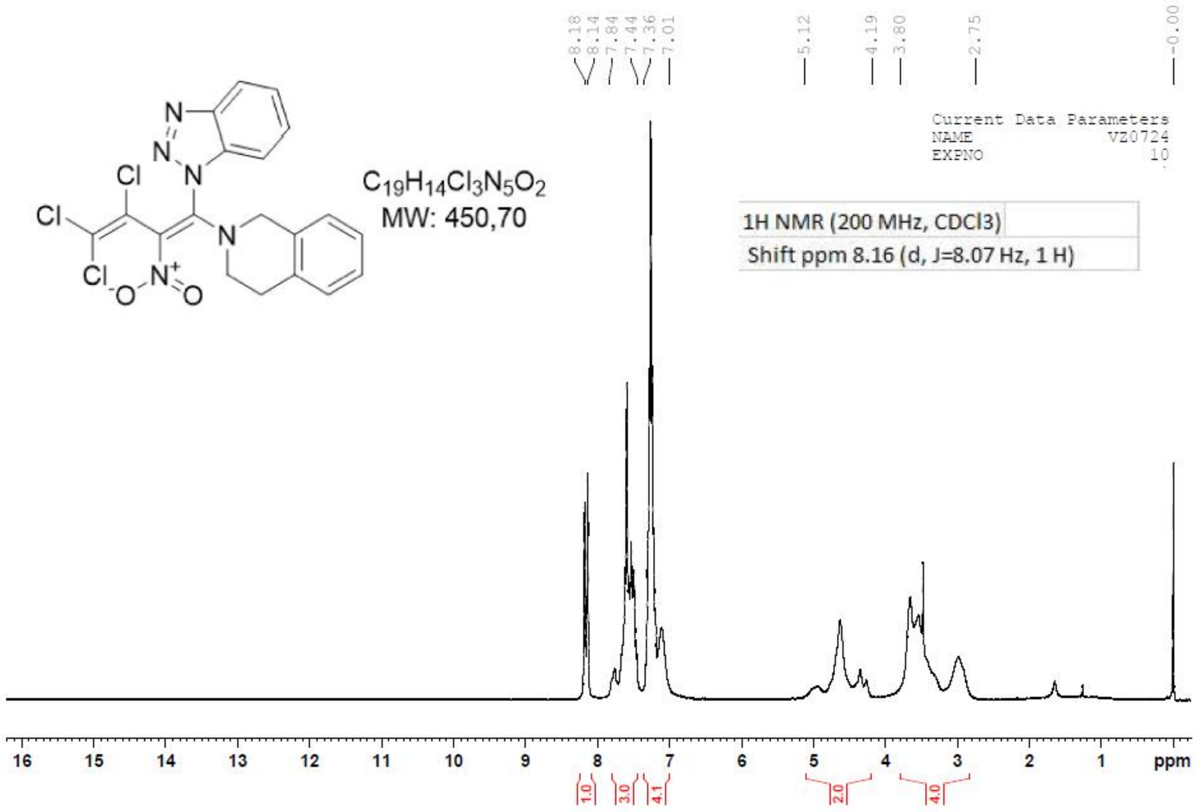

Figure S9. 200 MHz ^{1}H NMR spectrum in $CDCl_3$ for **4g**.

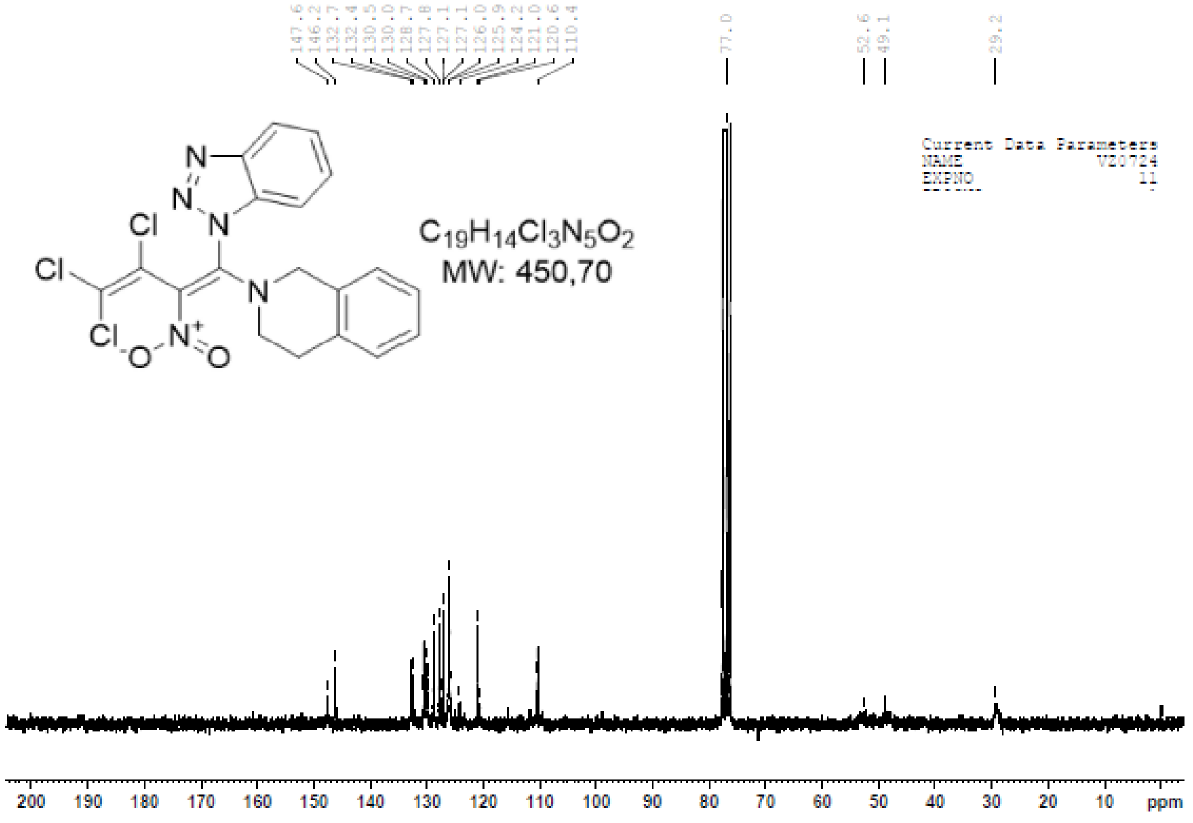

Figure S10. 5o MHz ^{13}C NMR spectrum in $CDCl_3$ for **4g**.

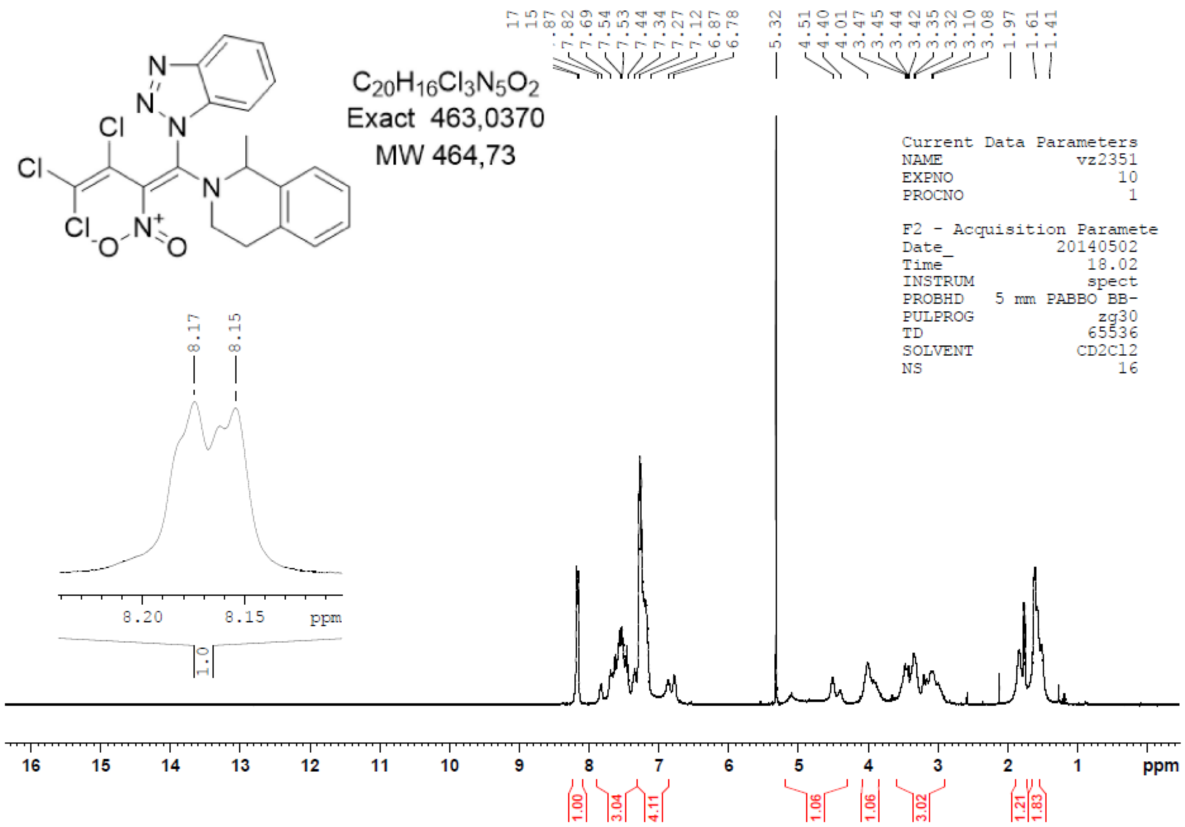

Figure S11. 400 MHz ^{1}H NMR spectrum in CD_2Cl_2 for **4h**.

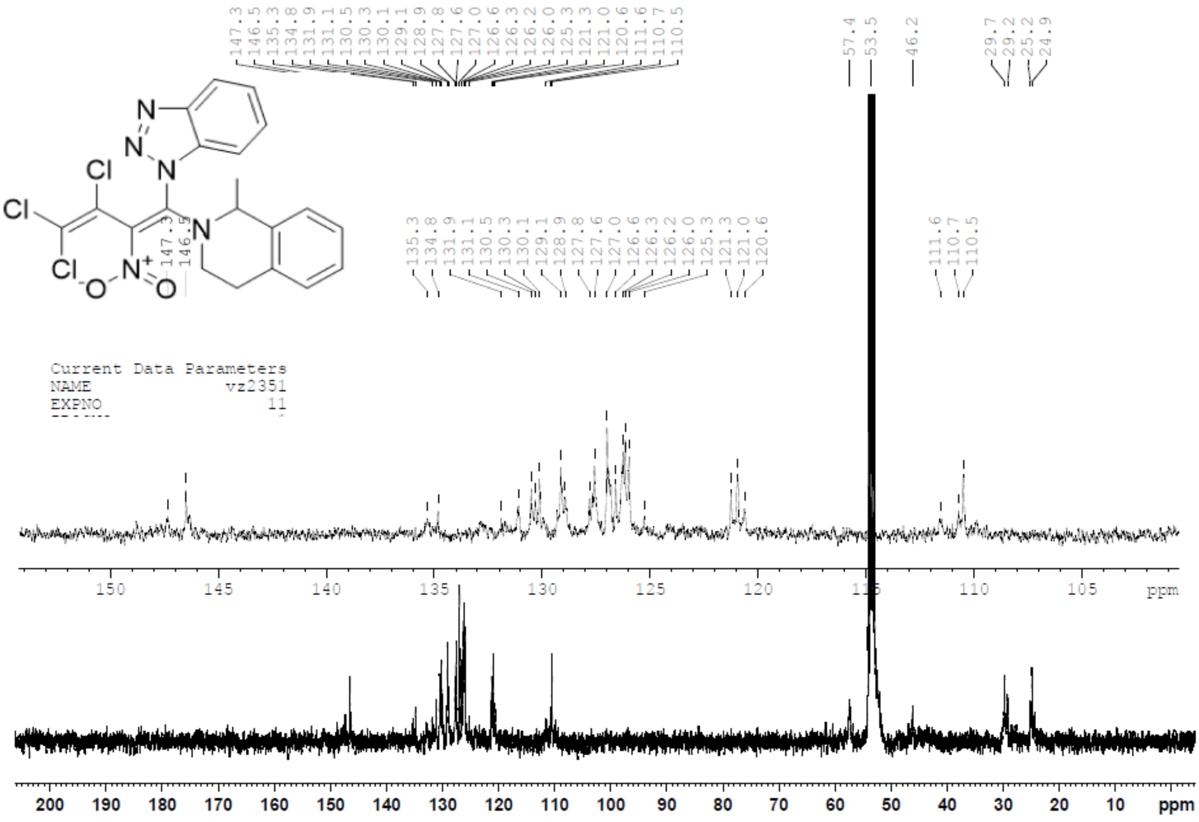

Figure S12. 100 MHz ^{13}C NMR spectrum in CD_2Cl_2 for **4h**.

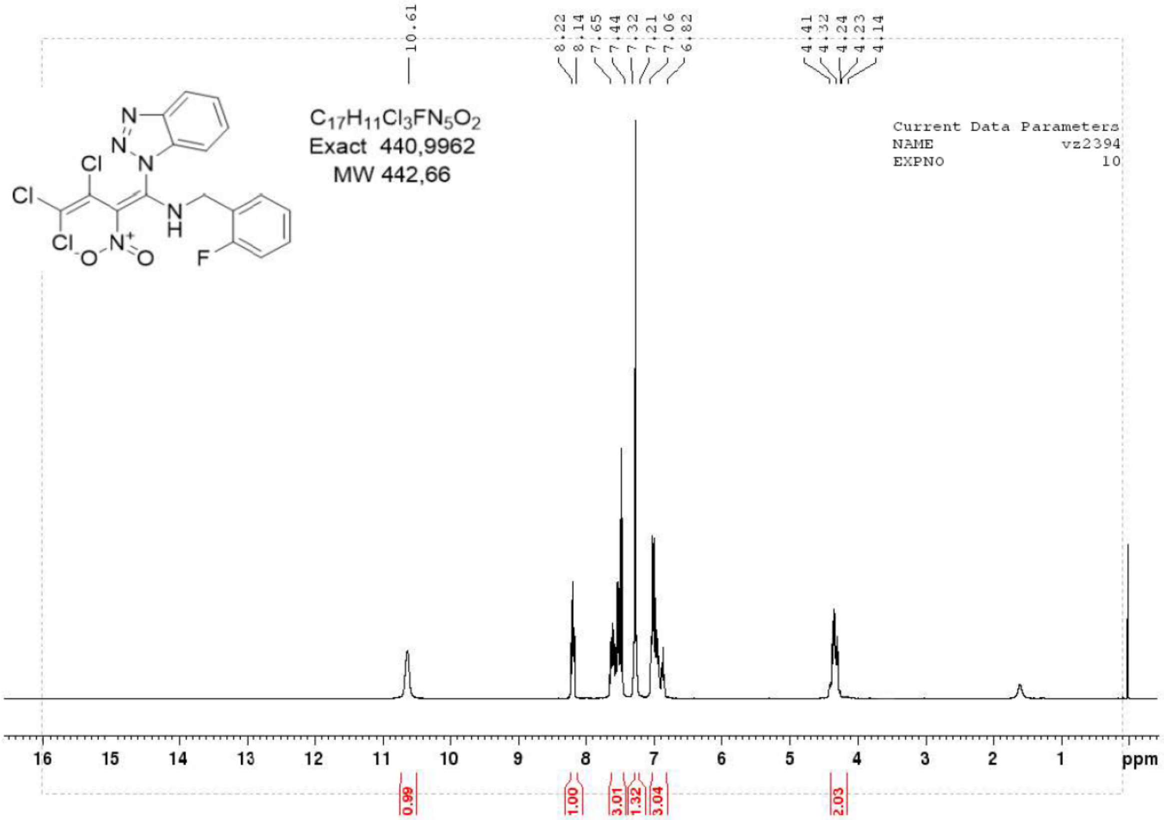

Figure S13. 400 MHz ^{1}H- NMR spectrum in $CDCl_3$ for **4l**.

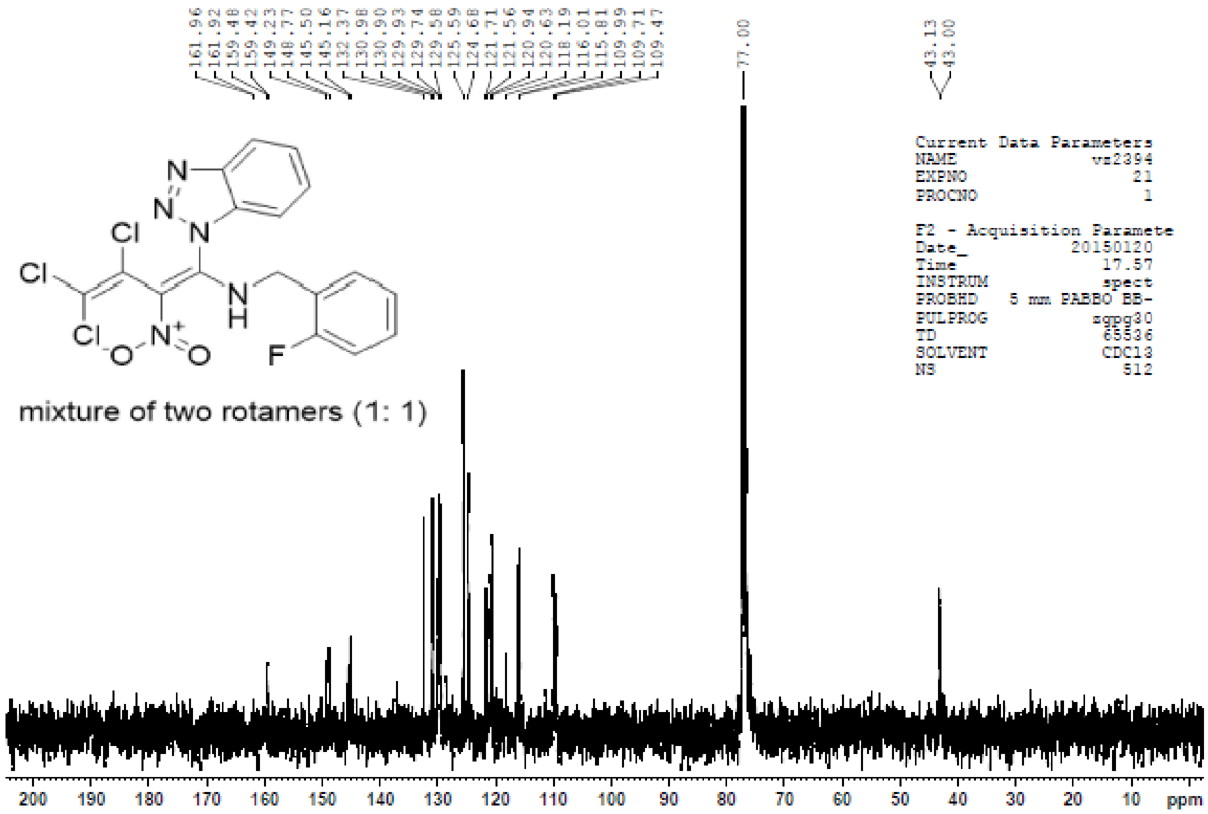

Figure S14. 100 MHz ^{13}C NMR spectrum in $CDCl_3$ for **4l**.

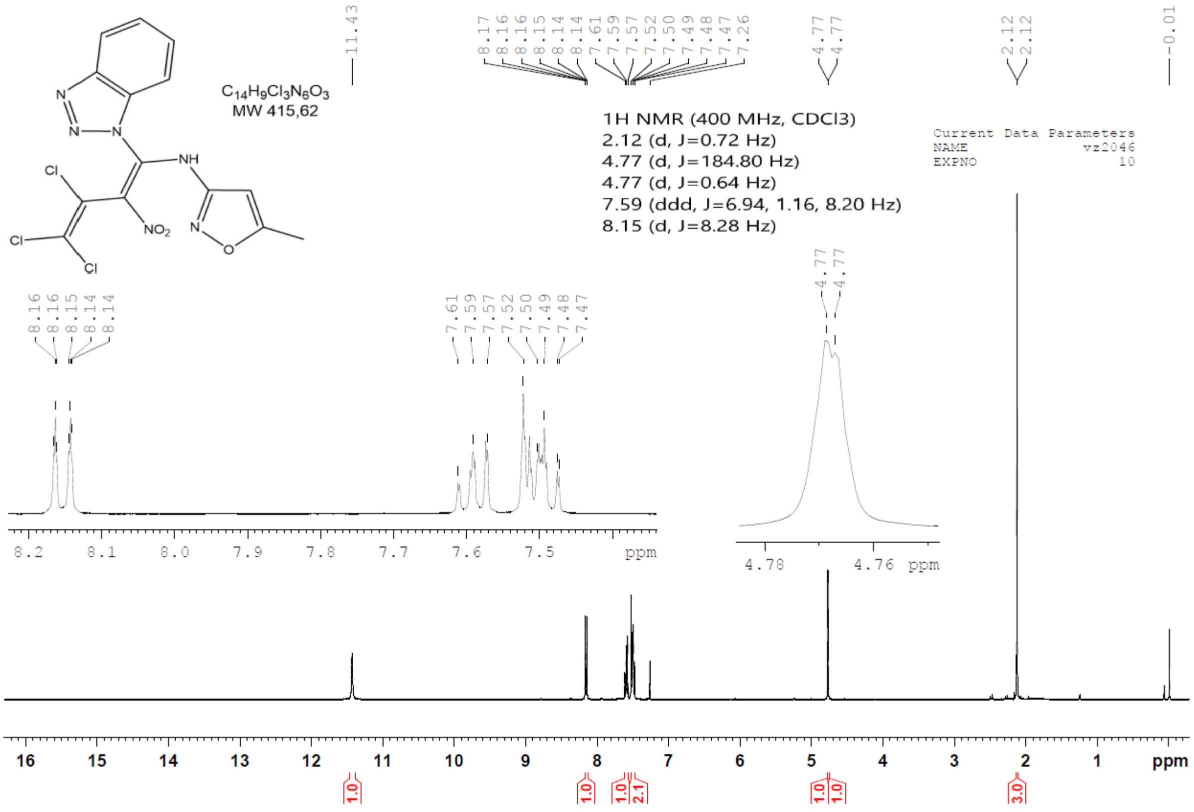

Figure S15. 400 MHz ^{1}H- NMR spectrum in $CDCl_3$ for **4o**.

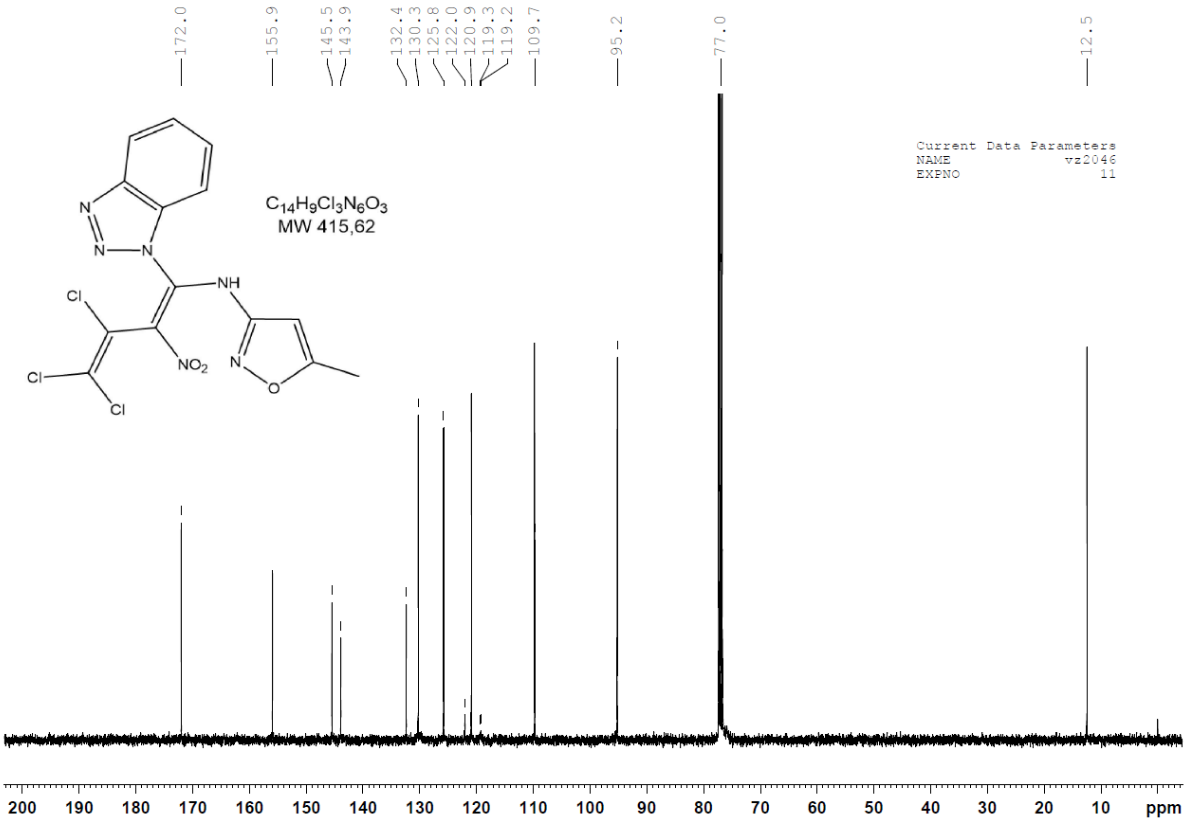

Figure S16. 100 MHz ^{13}C NMR spectrum in $CDCl_3$ for **4o**.

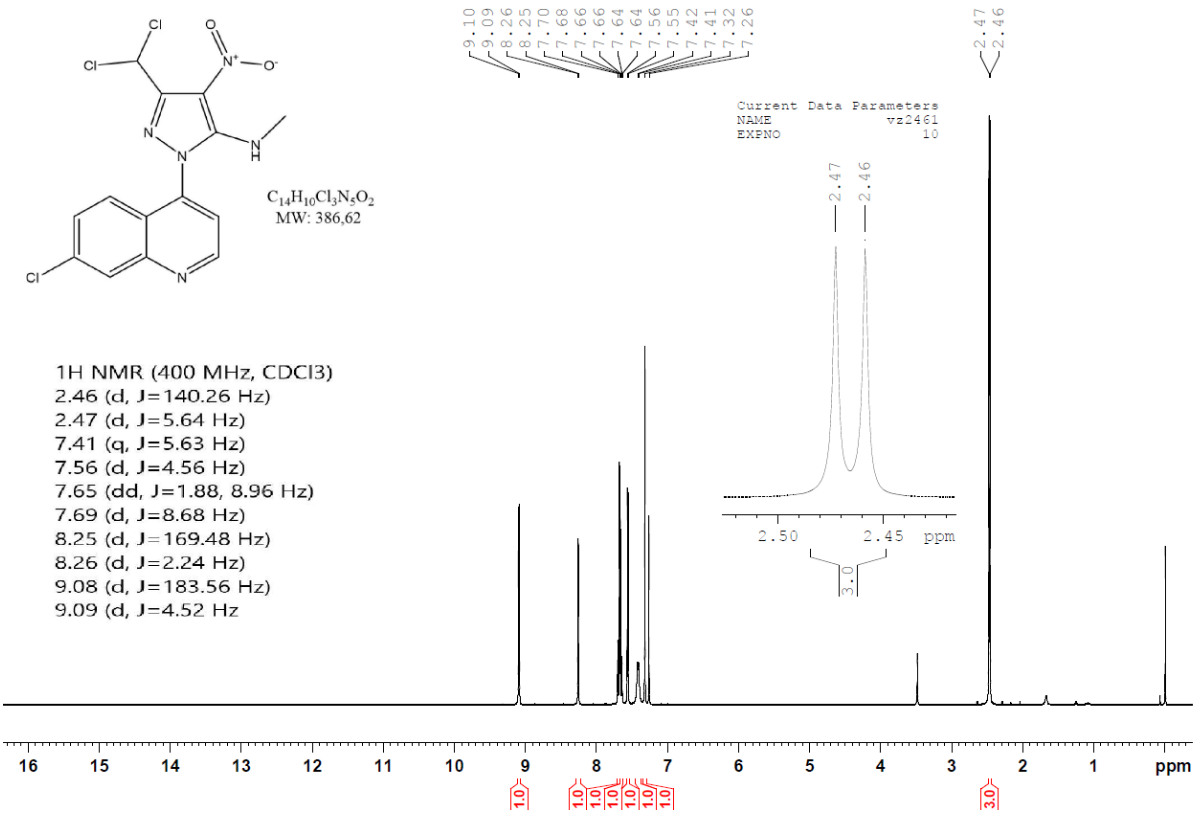

Figure S17. 400 MHz ^{1}H- NMR spectrum in $CDCl_3$ for **5a**.

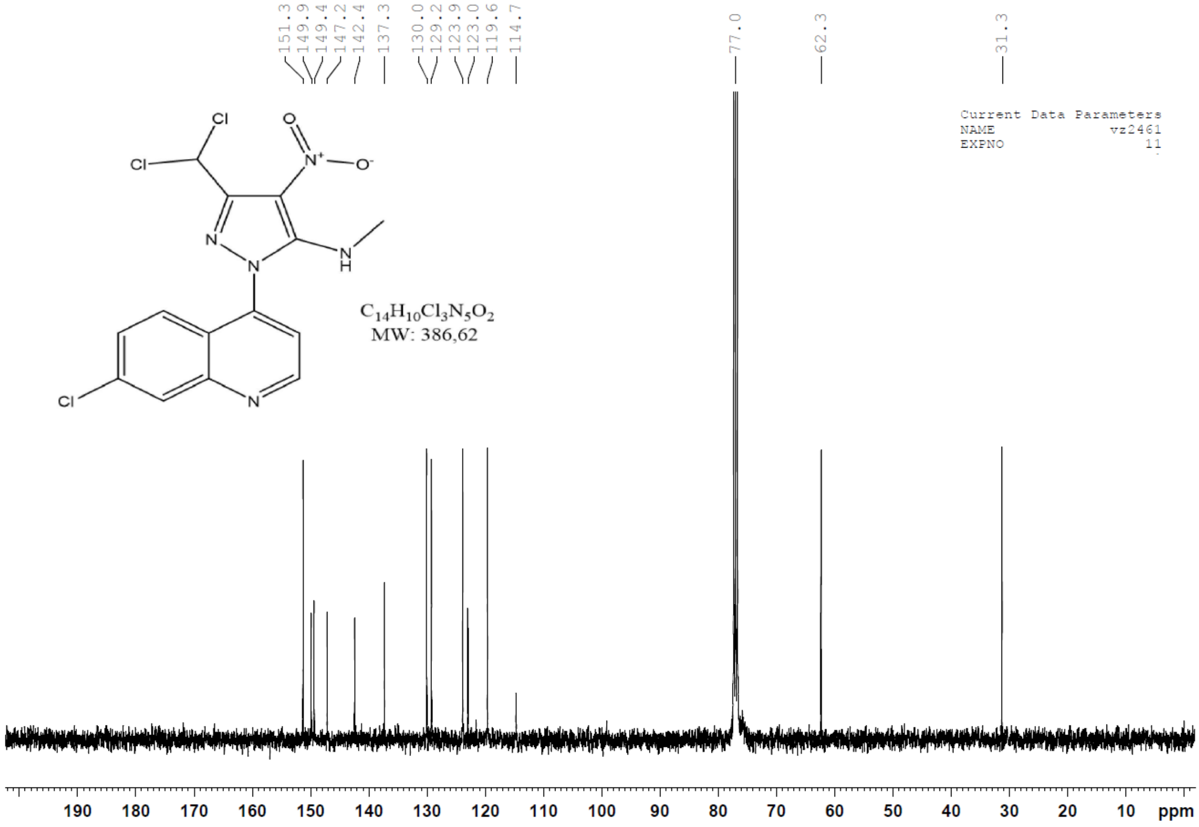

Figure S18. 100 MHz ^{13}C NMR spectrum in $CDCl_3$ for **5a**.

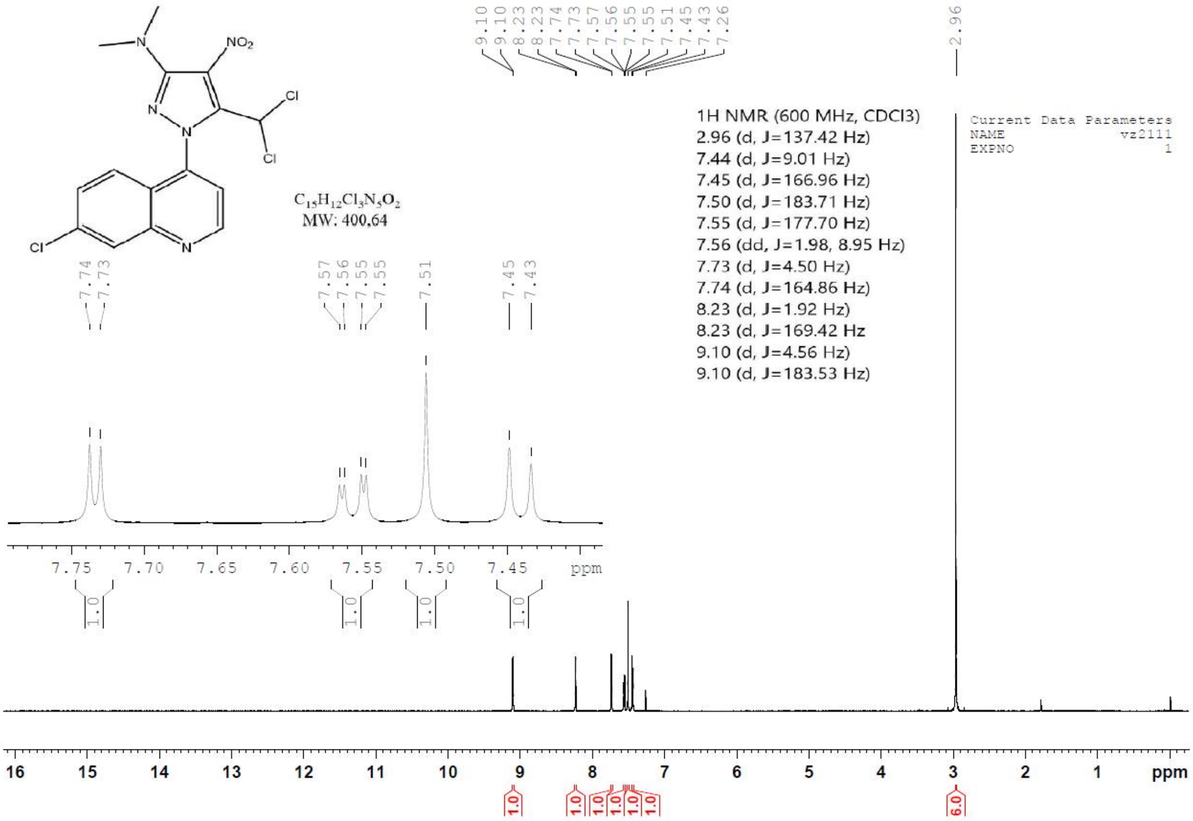

Figure S19. 600 MHz ^{1}H NMR spectrum in CDCl3 for **5b**.

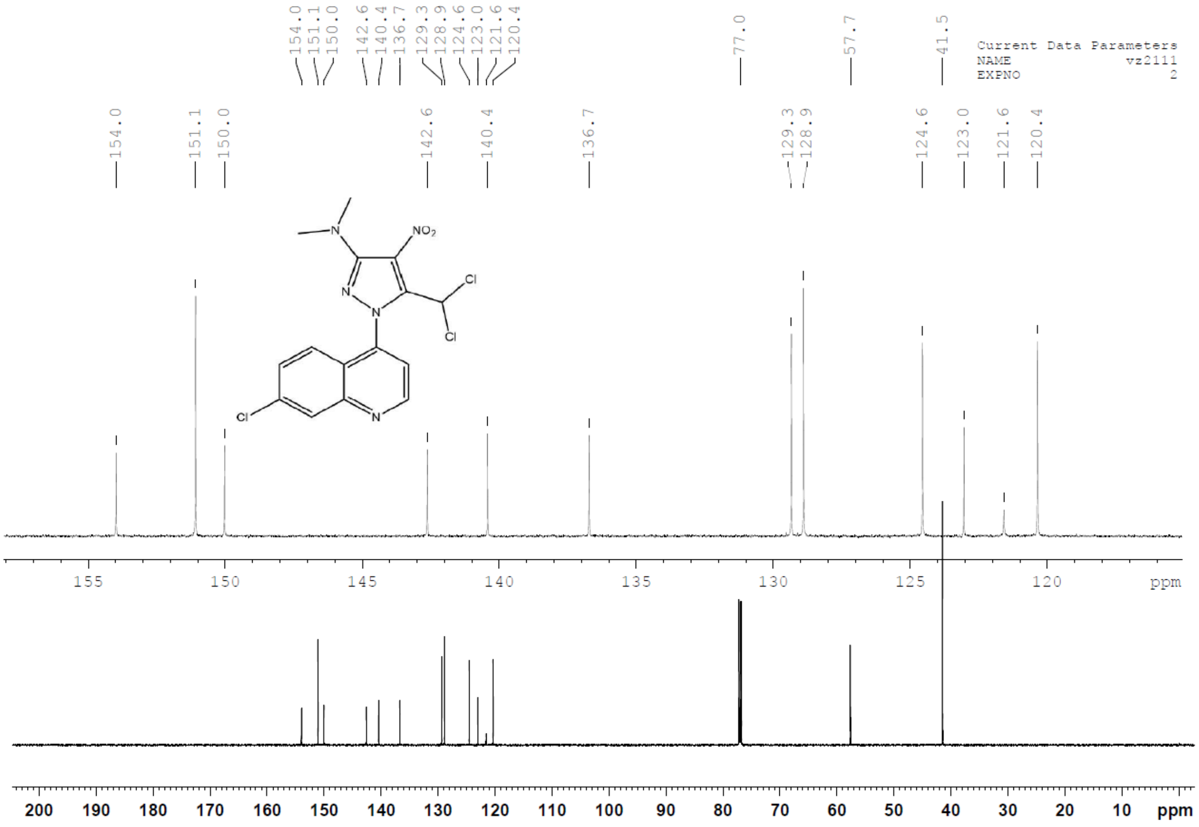

Figure S20. 150 MHz ^{13}C NMR spectrum in CDCl3 for **5b**.

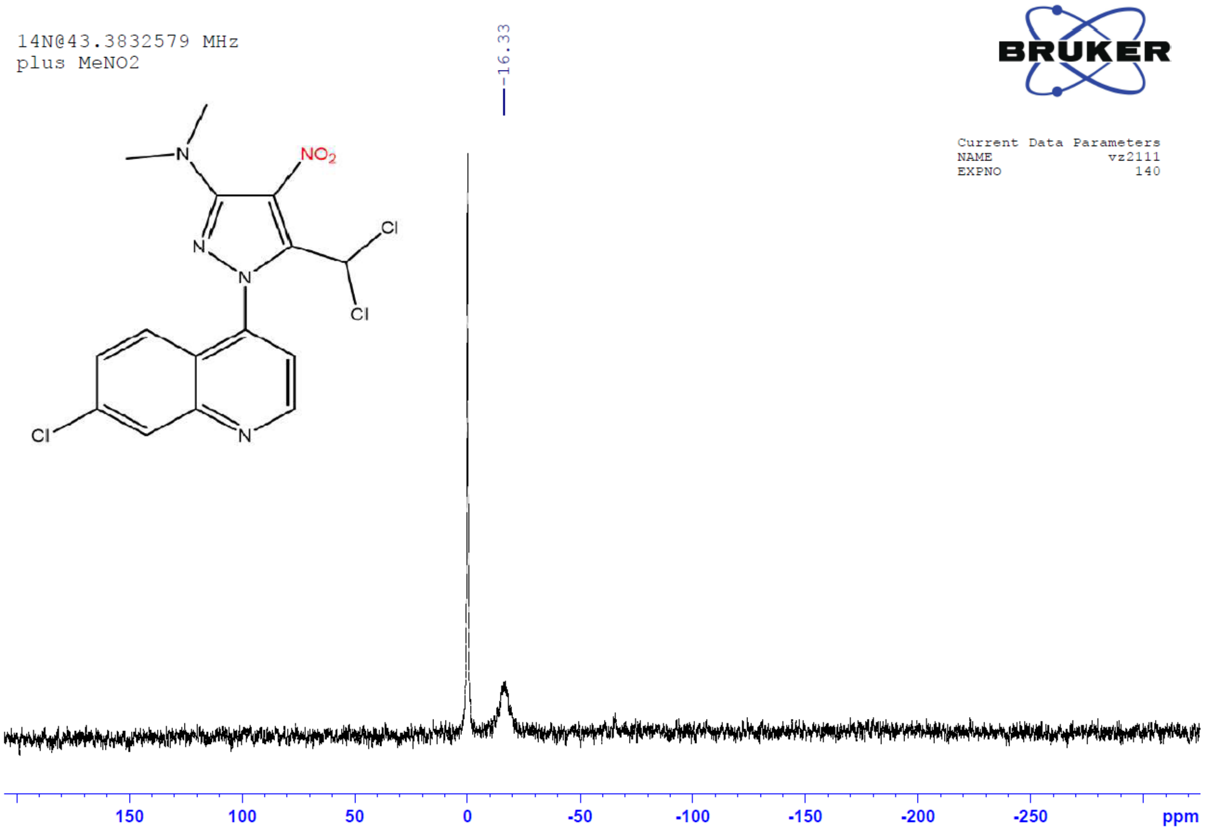

Figure S21. 43.4 MHz ^{14}N NMR spectrum in $CDCl_3$ with CH_3NO_2 (0.0 ppm) for **5b**.

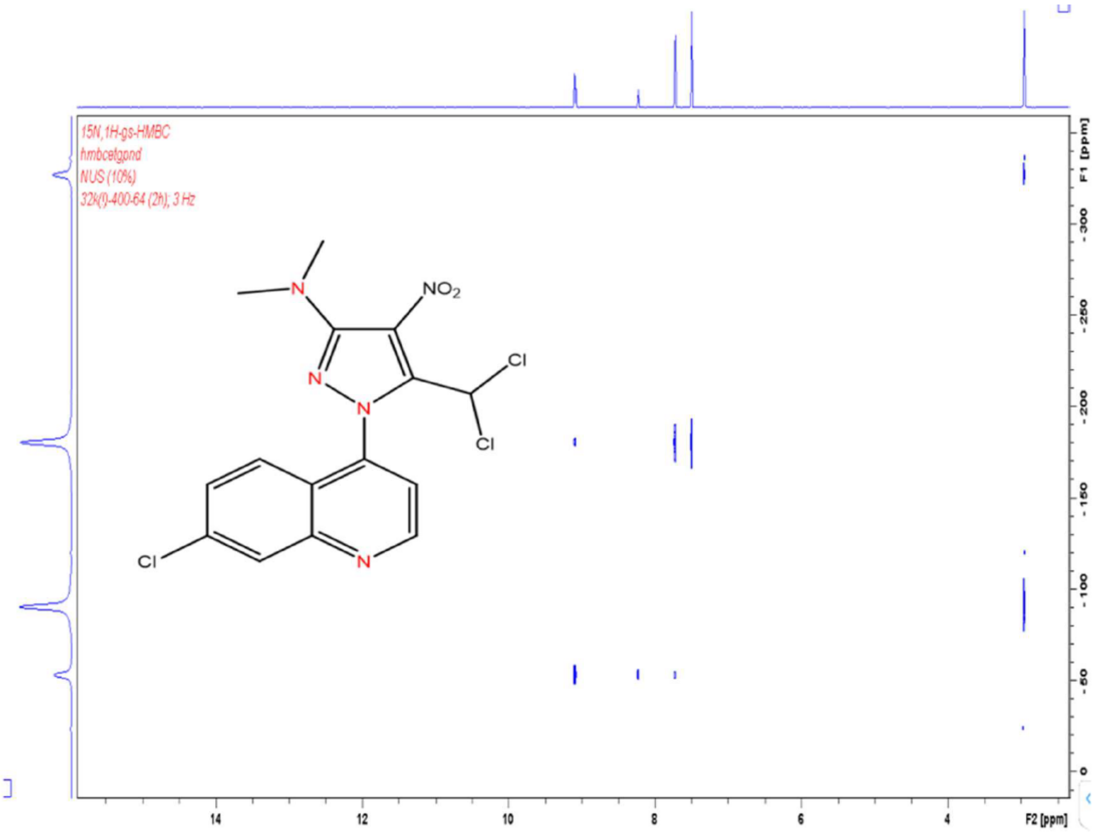

Figure S22. ^{15}N, ^{1}H-HMBC NMR spectrum in $CDCl_3$ for **5b**.

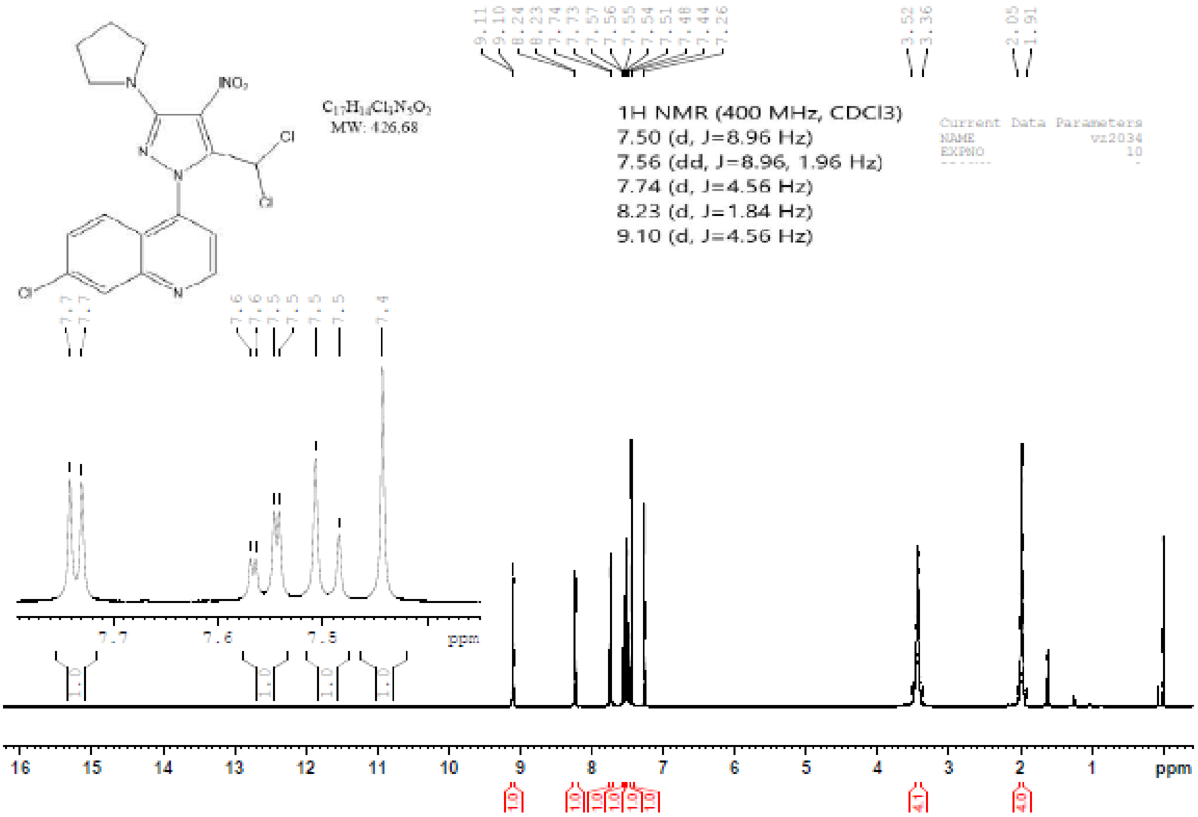

Figure S23. 400 MHz ^{1}H NMR spectrum in $CDCl_3$ for **5c**.

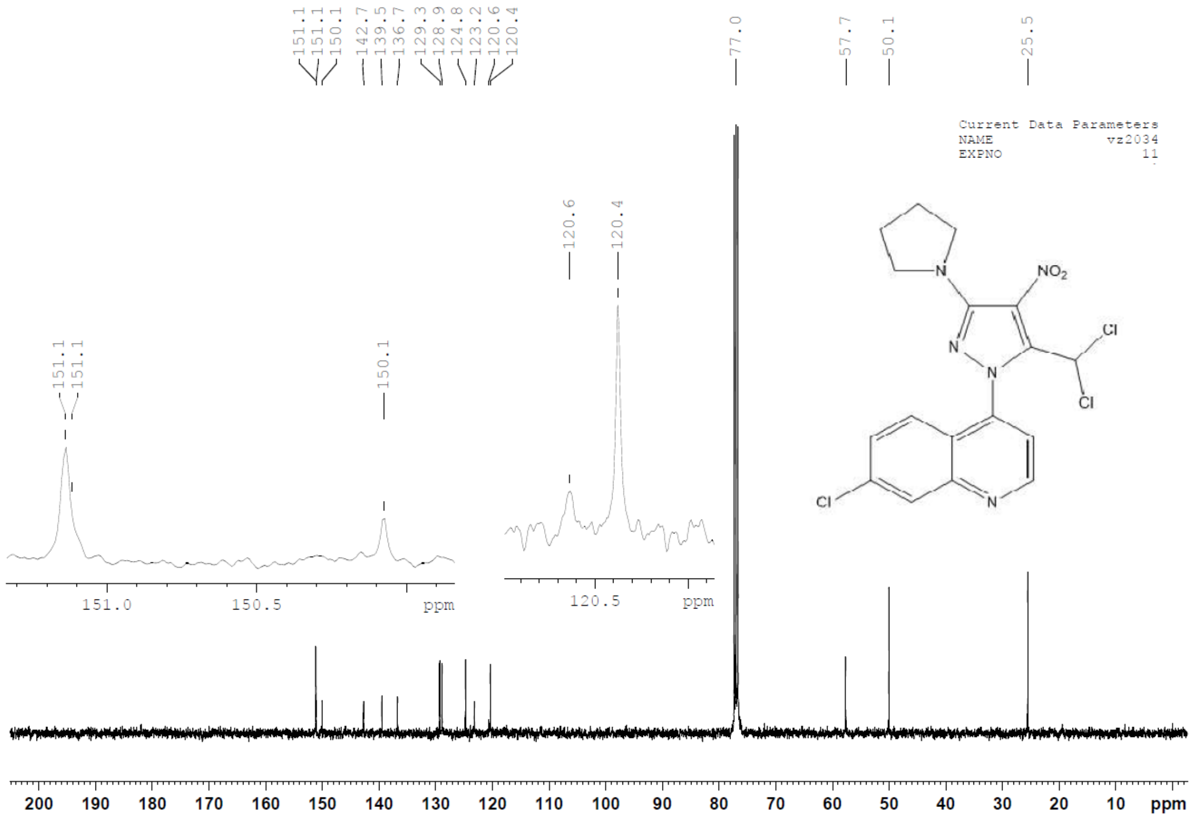

Figure S24. 100 MHz ^{13}C NMR spectrum in $CDCl_3$ for **5c**.

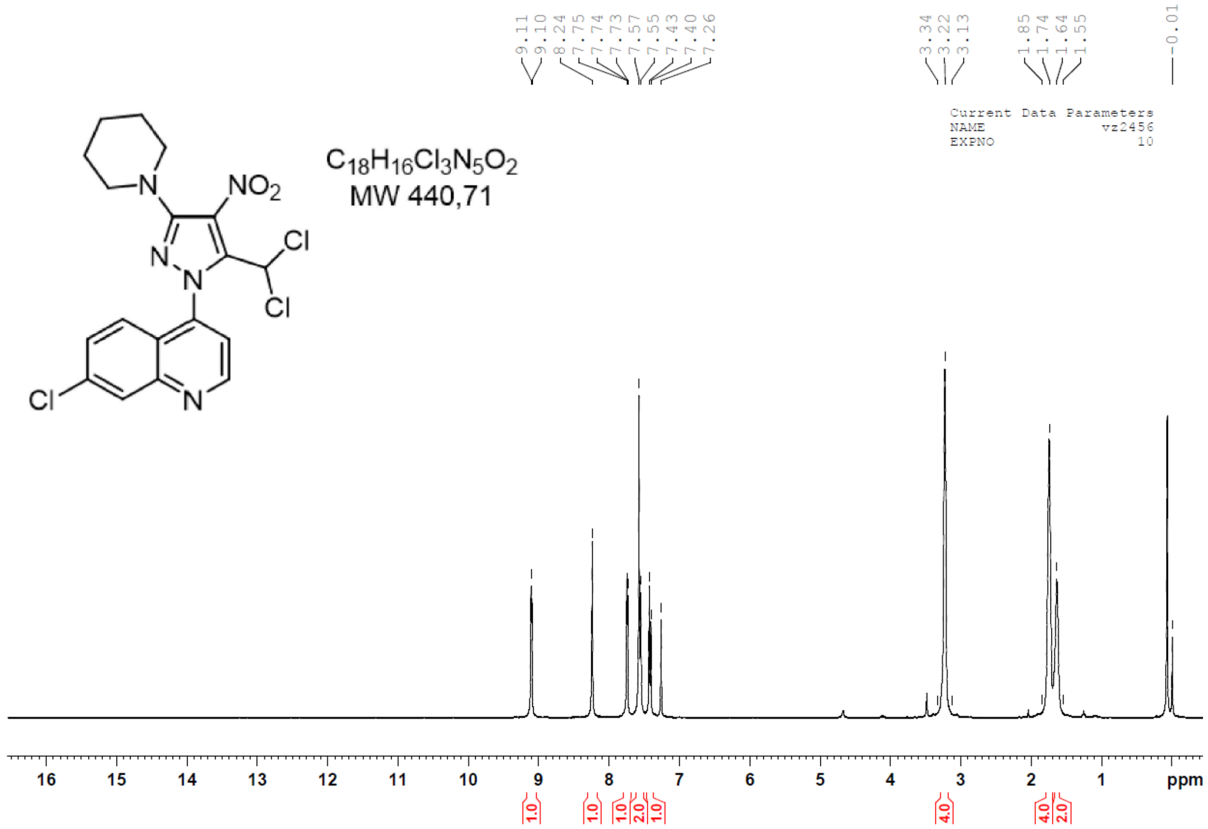

Figure S25. 400 MHz ^{1}H NMR spectrum in $CDCl_3$ for **5d**.

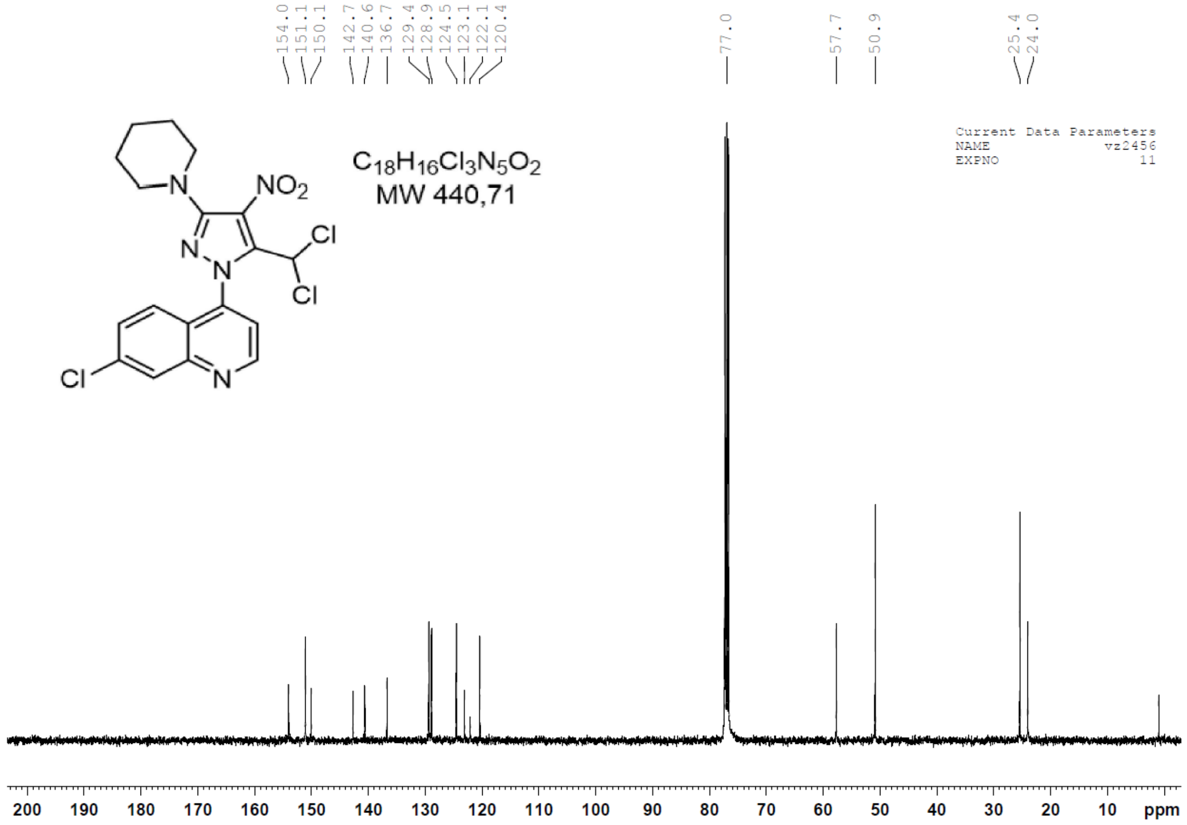

Figure S26. 100 MHz ^{13}C NMR spectrum in $CDCl_3$ for **5d**.

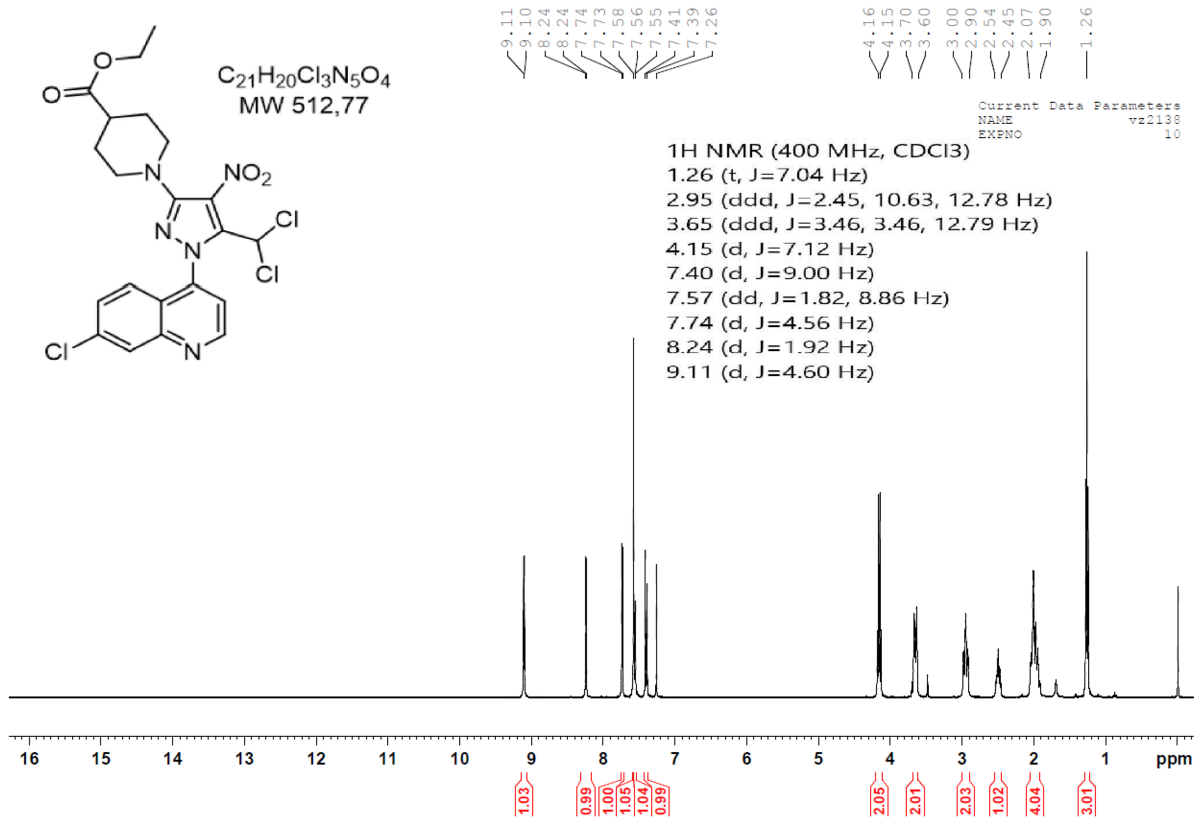

Figure S27. 400 MHz ^{1}H NMR spectrum in $CDCl_3$ for **5e**.

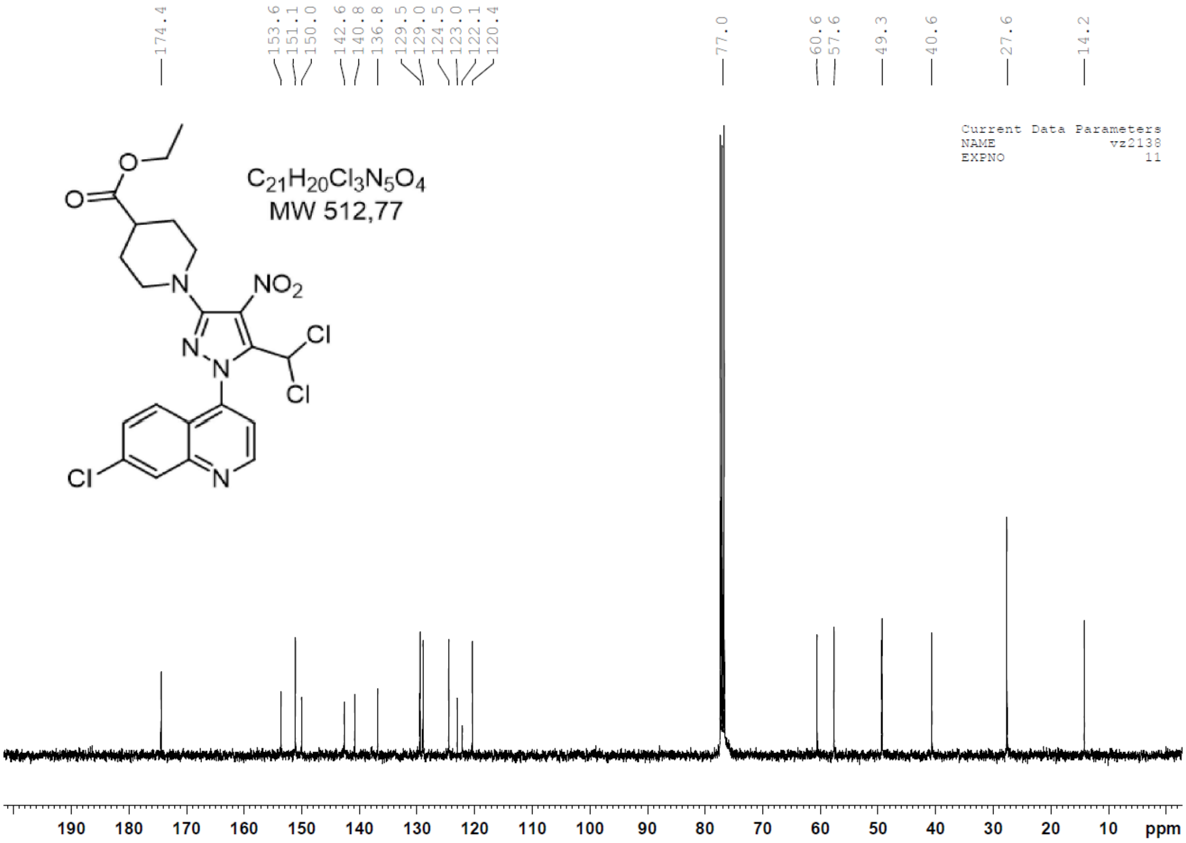

Figure S28. 100 MHz ^{13}C NMR spectrum in $CDCl_3$ for **5e**.

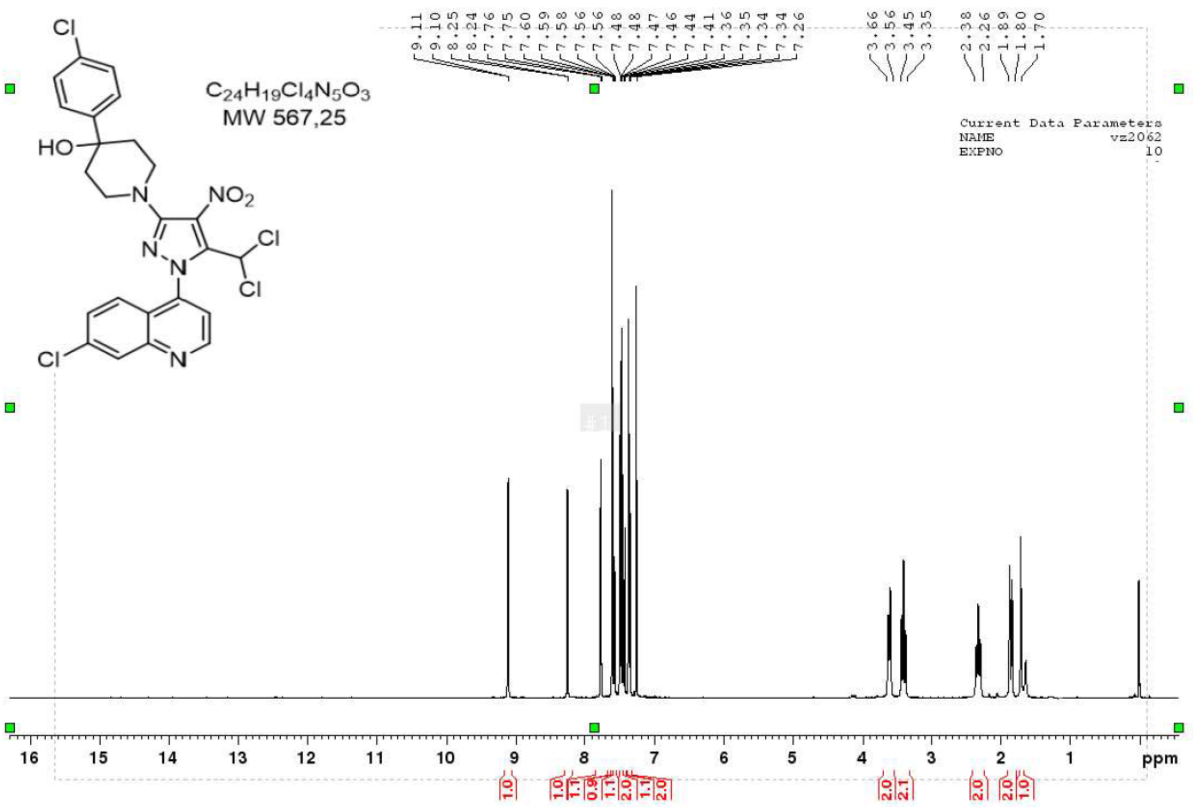

Figure S29. 400 MHz ^{1}H NMR spectrum in CDCl3 for **5f**.

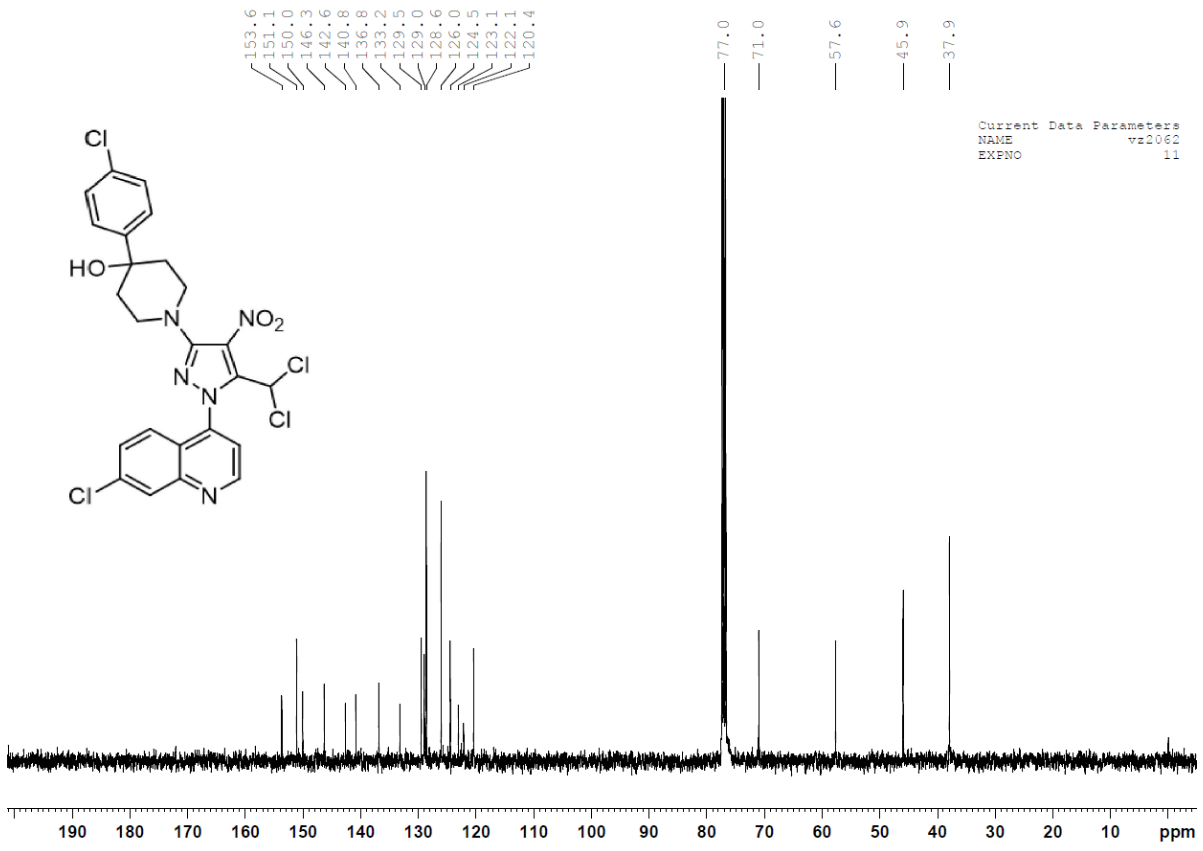

Figure S30. 100 MHz ^{13}C NMR spectrum in CDCl3 for **5f**.

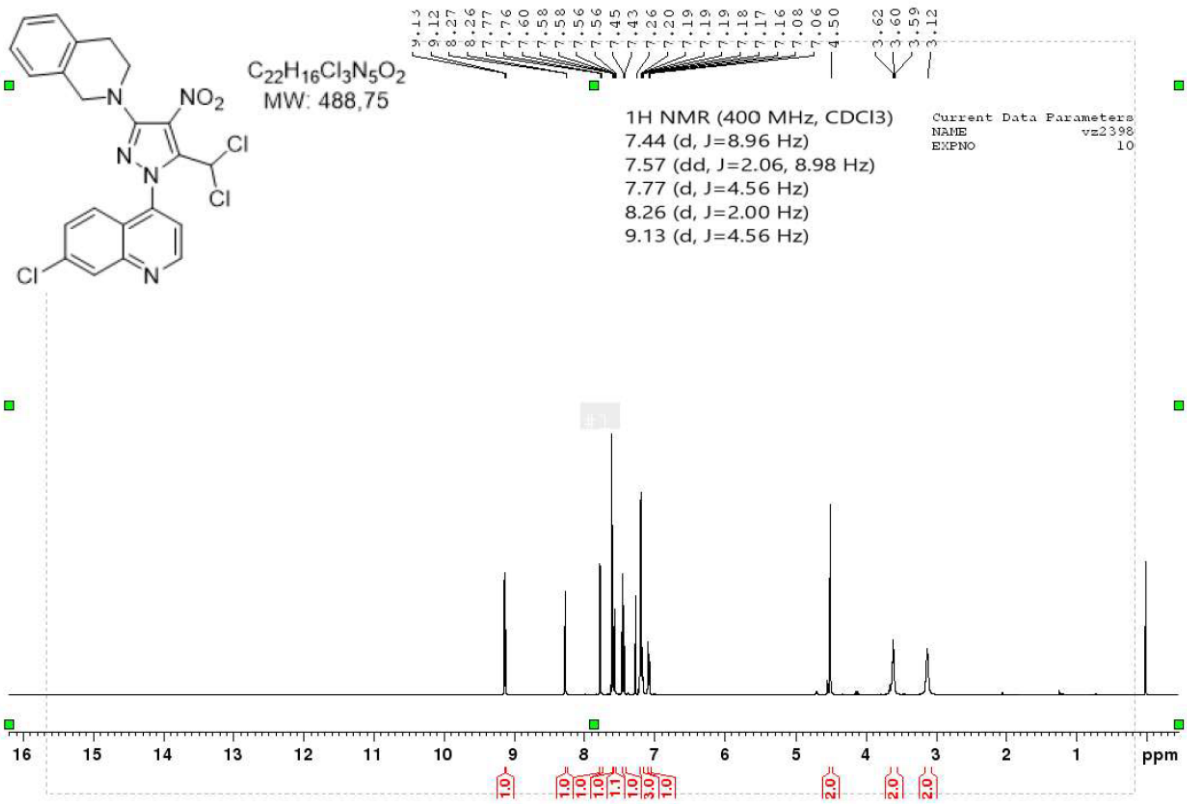

Figure S31. 400 MHz ^{1}H NMR spectrum in $CDCl_3$ for **5g**.

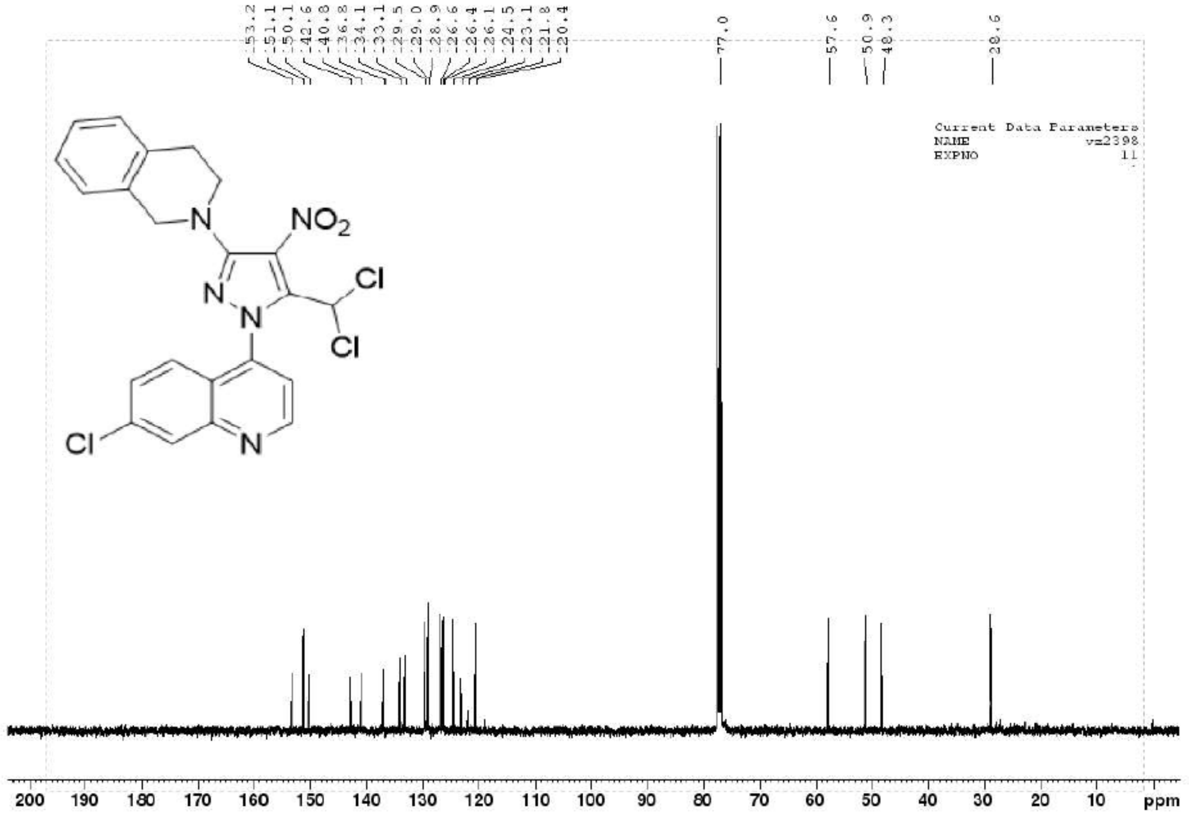

Figure S32. 100 MHz ^{13}C NMR spectrum in $CDCl_3$ for **5g**.

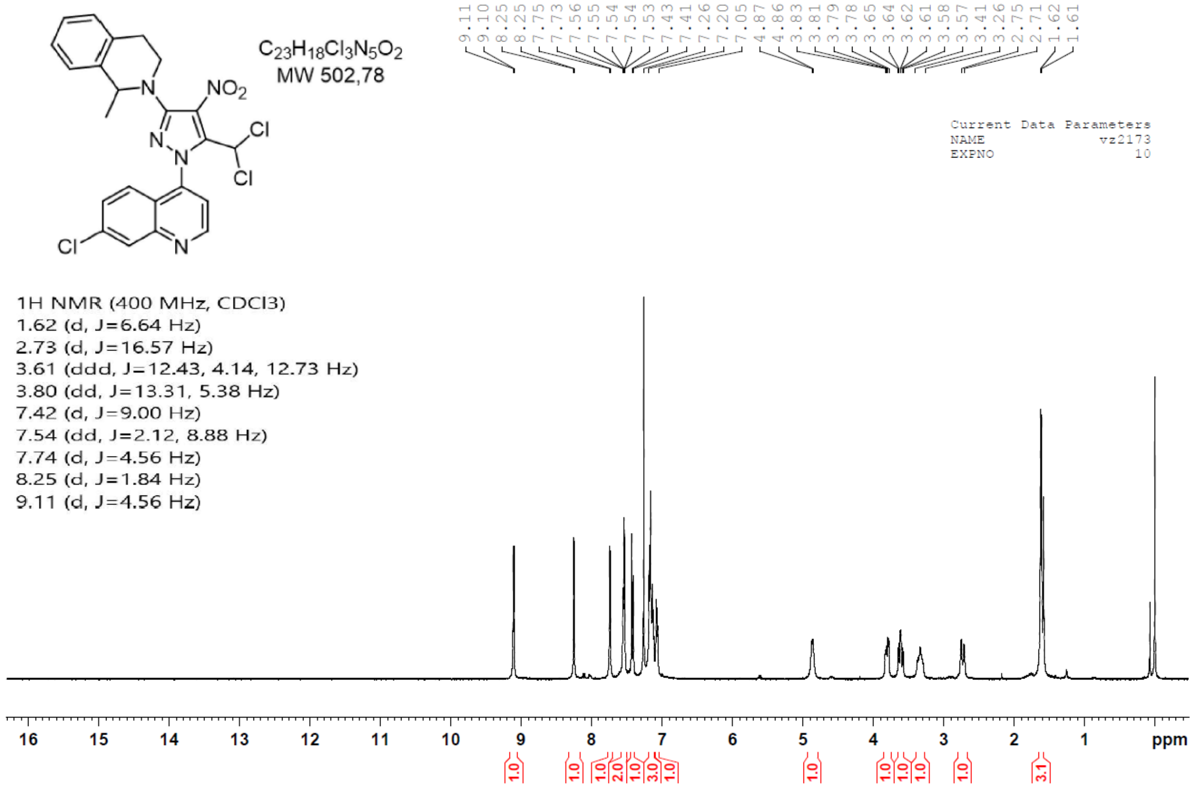

Figure S33. 400 MHz ^{1}H NMR spectrum in $CDCl_3$ for **5h**.

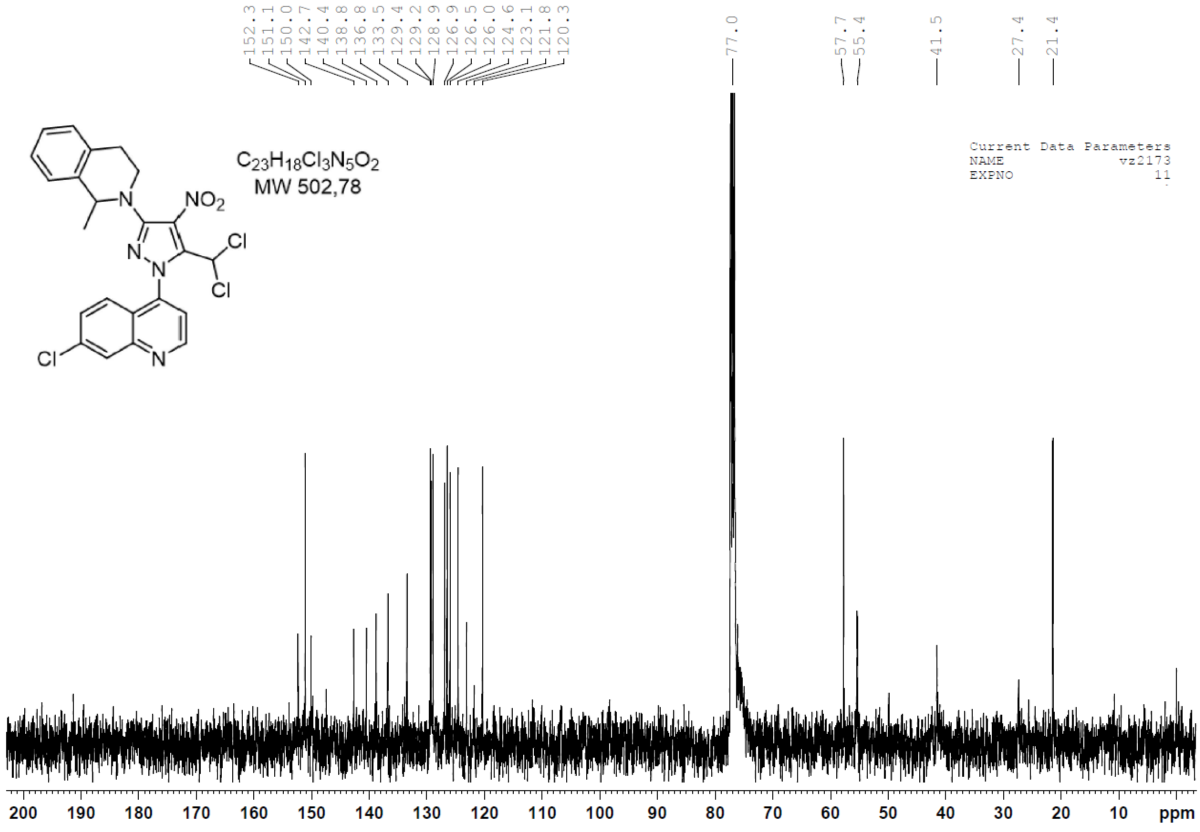

Figure S34. 100 MHz ^{13}C NMR spectrum in $CDCl_3$ for **5h**.

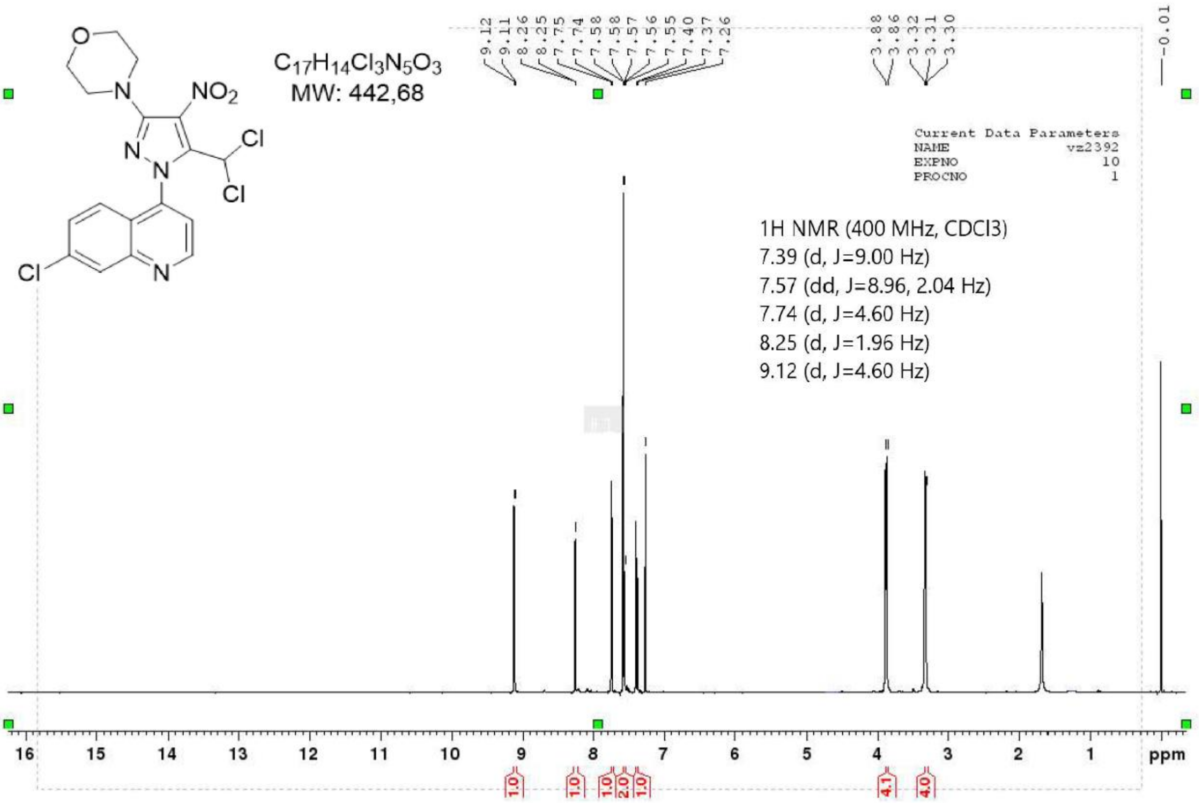

Figure S35. 400 MHz ^{1}H NMR spectrum in CDCl3 for **5i**.

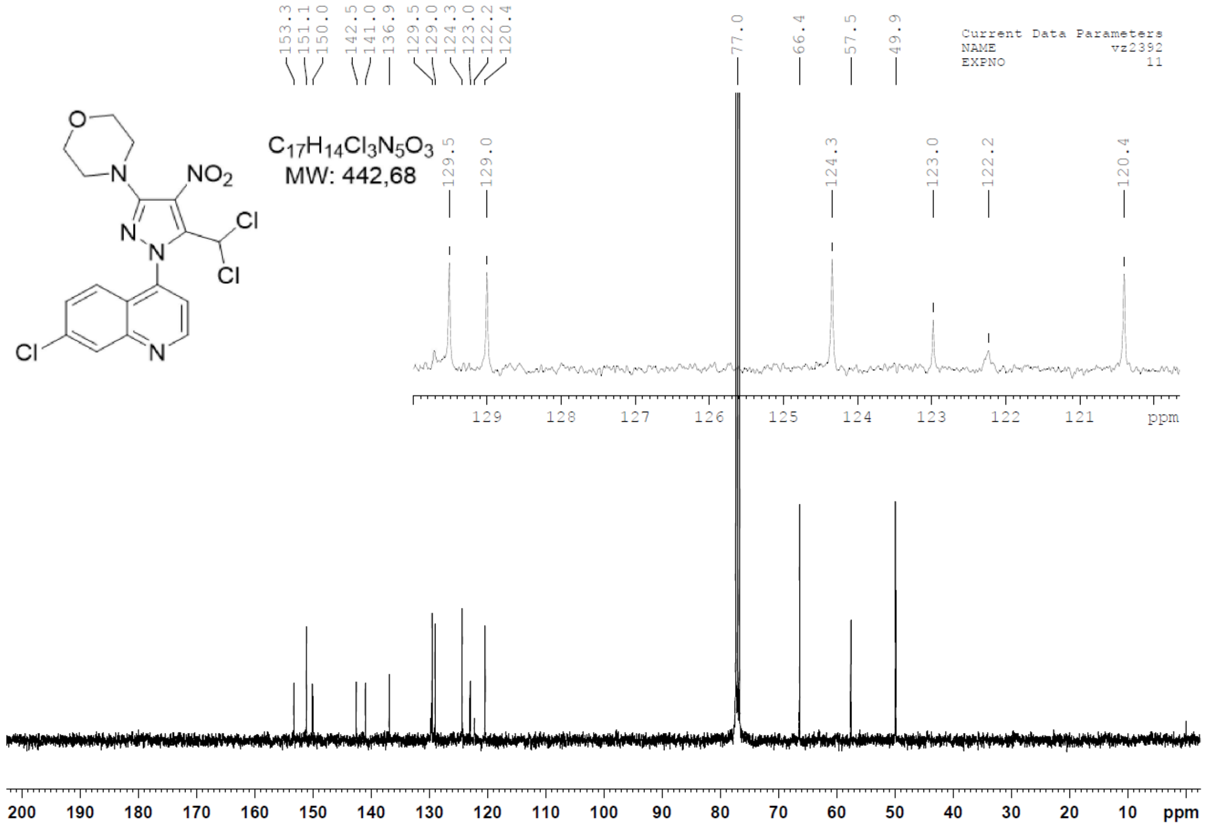

Figure S36. 100 MHz ^{13}C NMR spectrum in CDCl3 for **5i**.

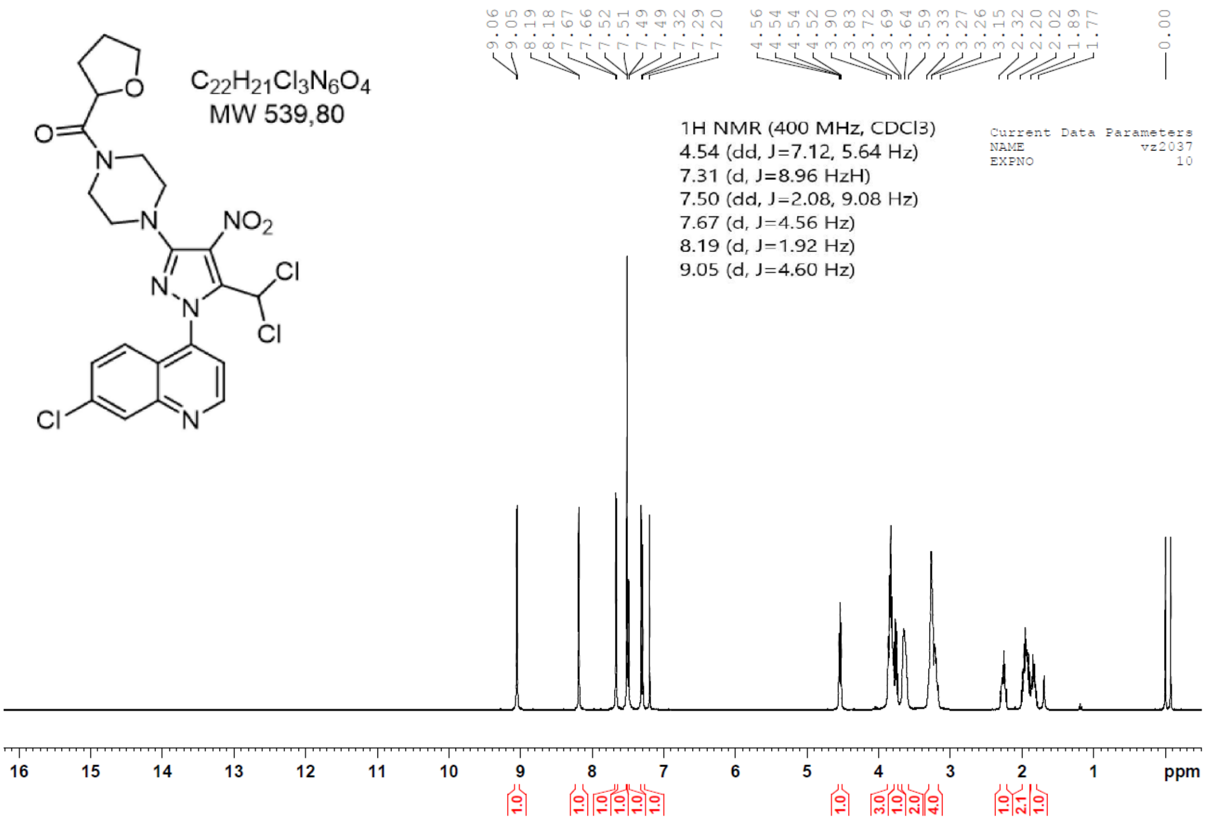

Figure S37. 400 MHz ^{1}H NMR spectrum in $CDCl_3$ for **5j**.

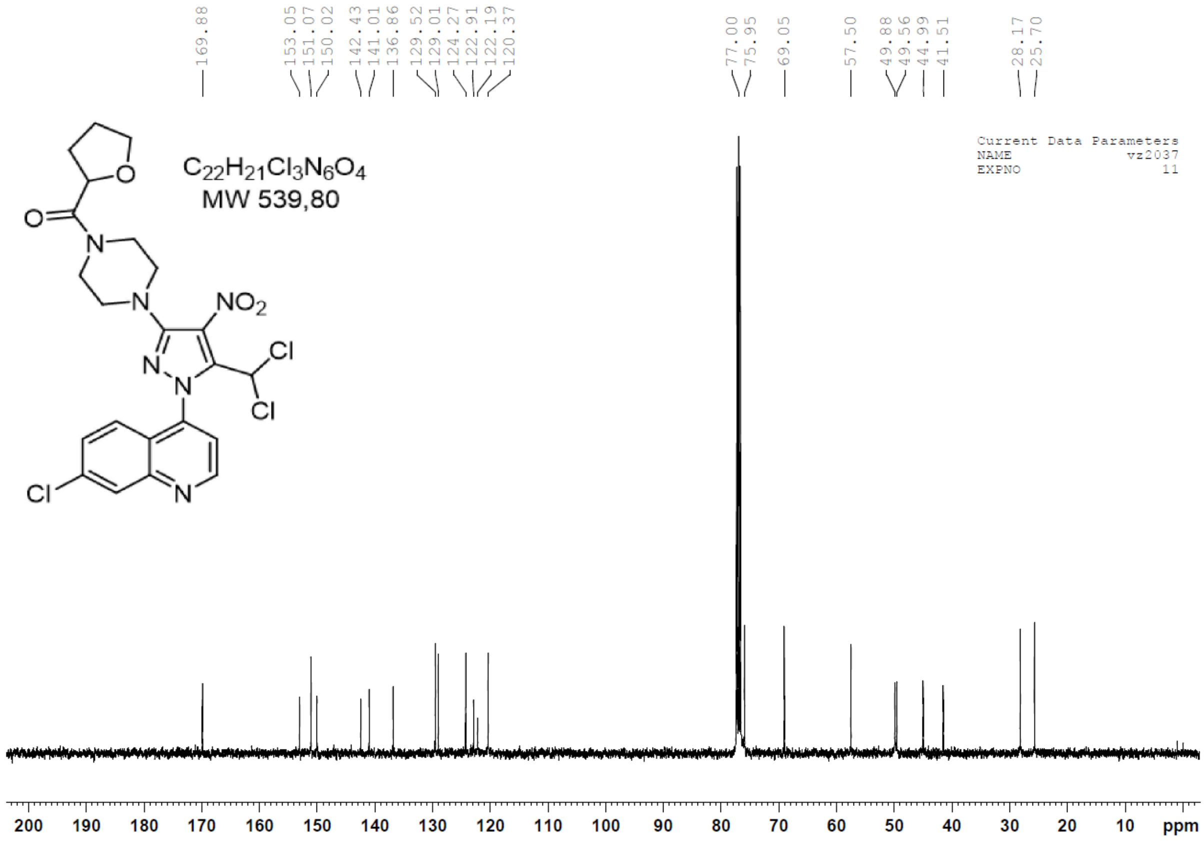

Figure S38. 100 MHz ^{13}C NMR spectrum in $CDCl_3$ for **5j**.

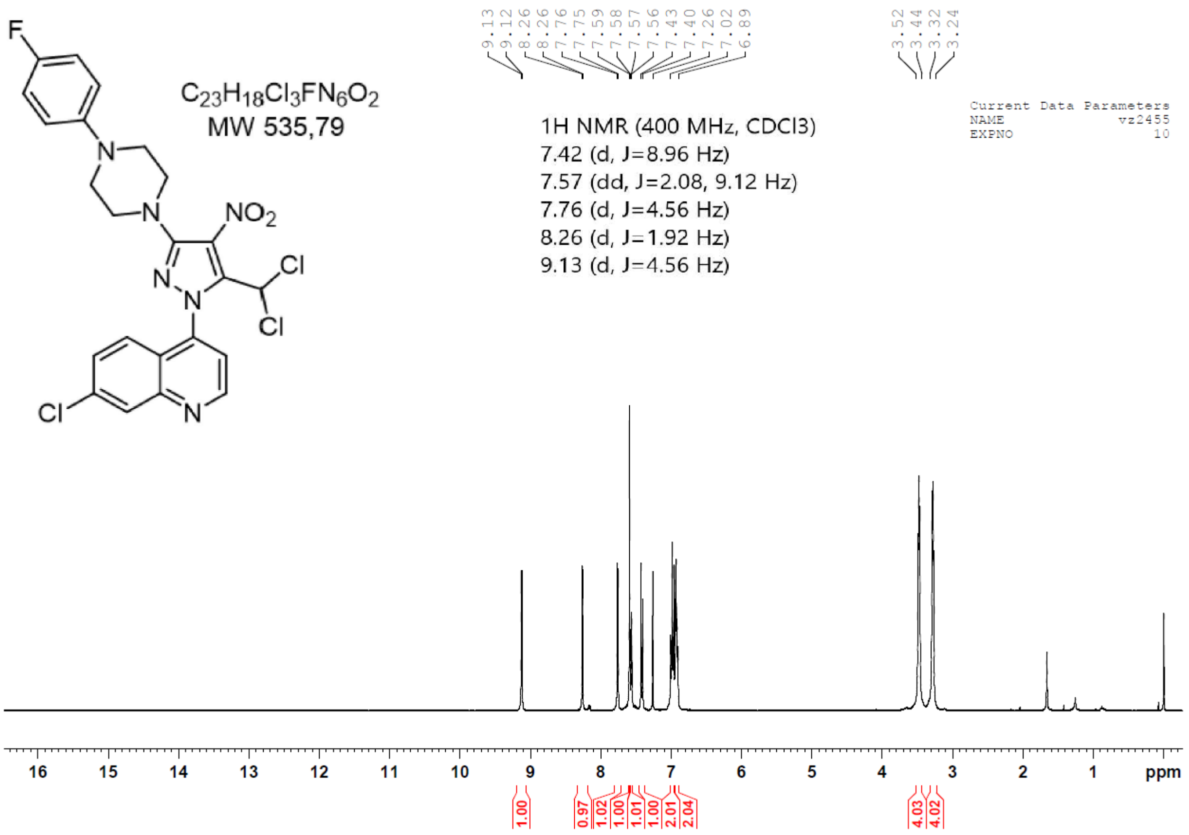

Figure S39. 400 MHz ^{1}H NMR spectrum in $CDCl_3$ for **5k**.

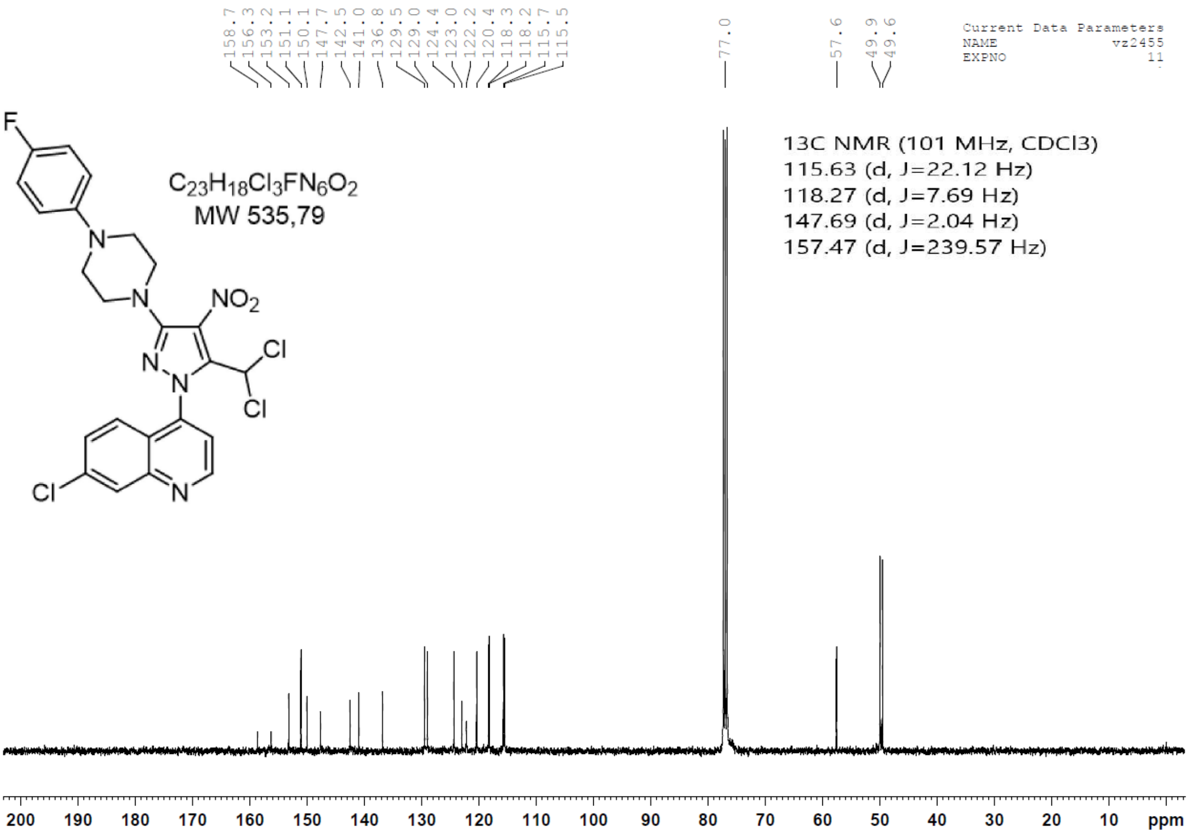

Figure S40. 100 MHz ^{13}C NMR spectrum in $CDCl_3$ for **5k**.

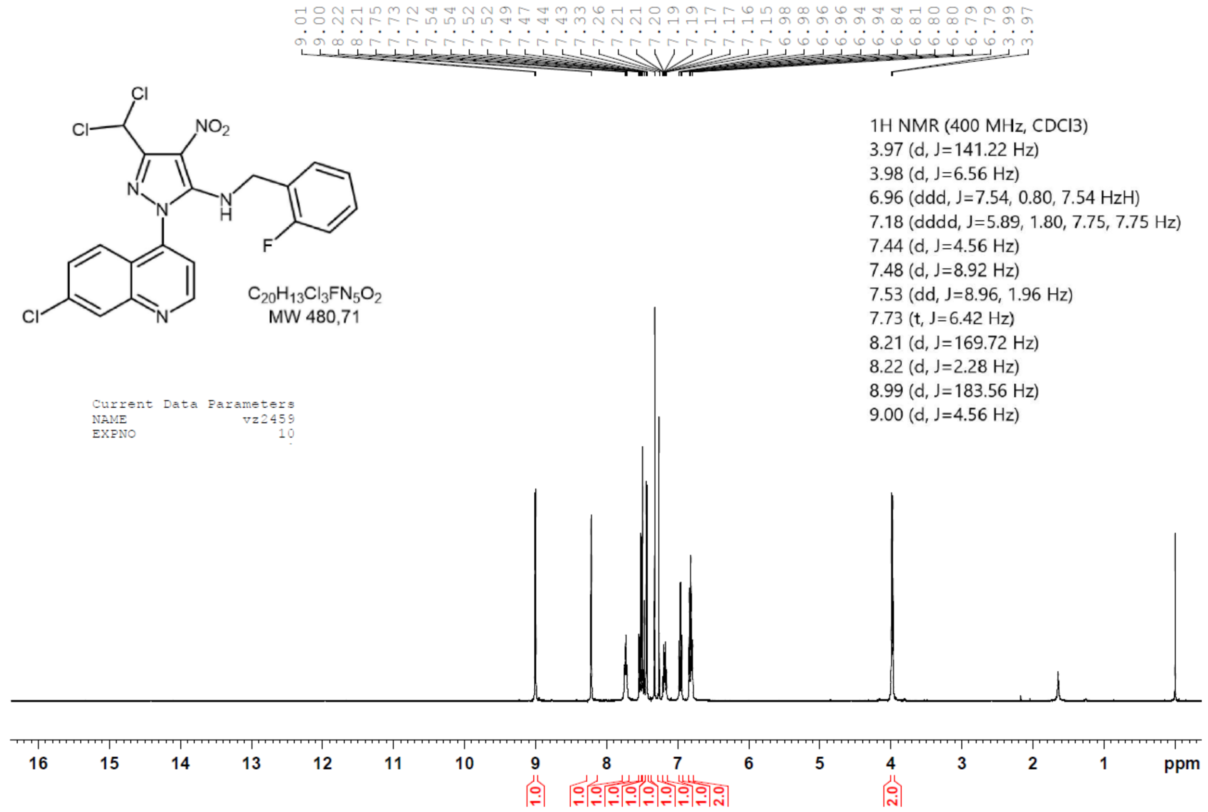

Figure S41. 400 MHz ^{1}H NMR spectrum in $CDCl_3$ for **5l**.

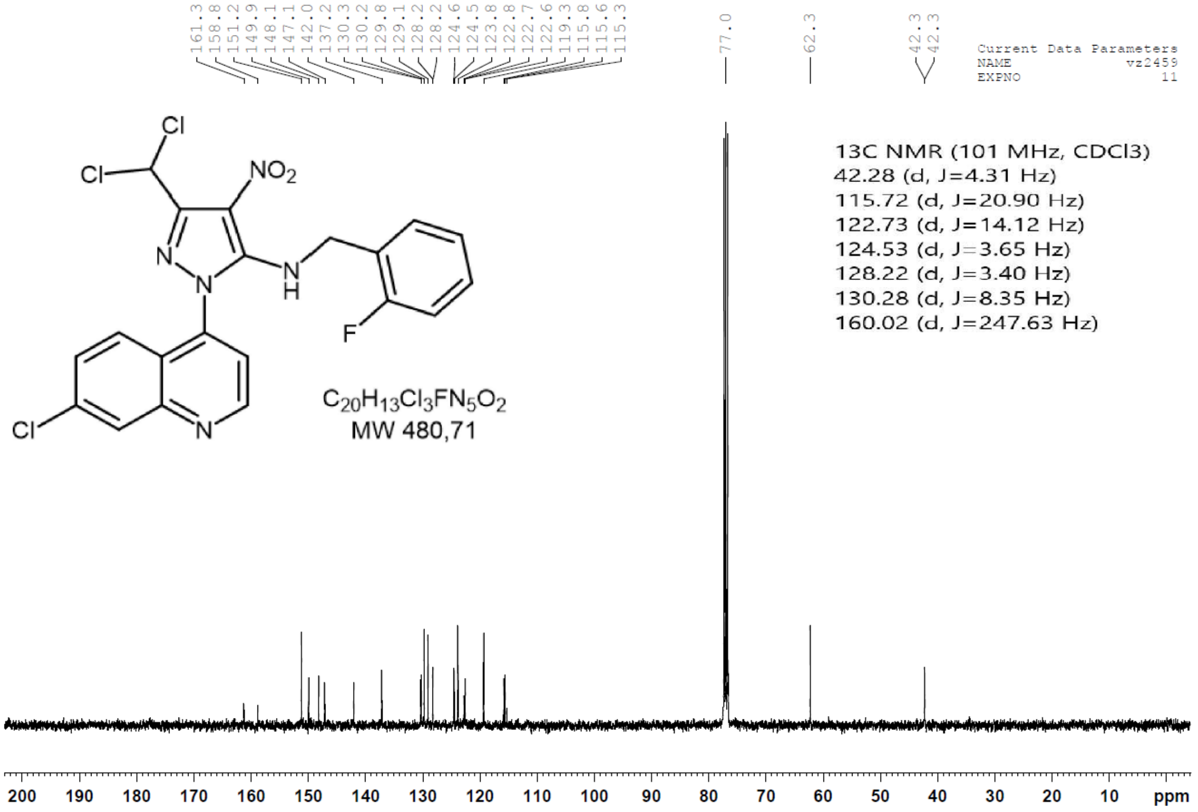

Figure S42. 100 MHz ^{13}C NMR spectrum in $CDCl_3$ for **5l**.

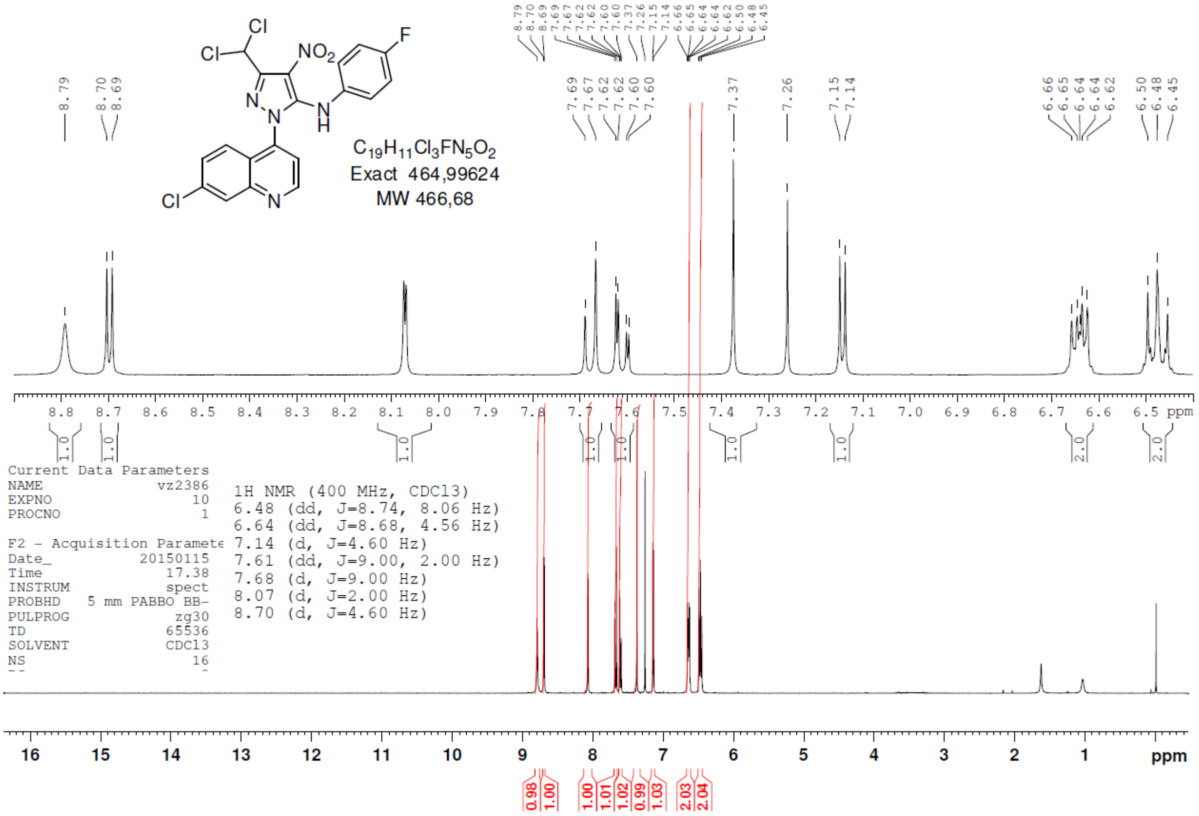

Figure S43. 400 MHz ^{1}H NMR spectrum in $CDCl_3$ for **5m**.

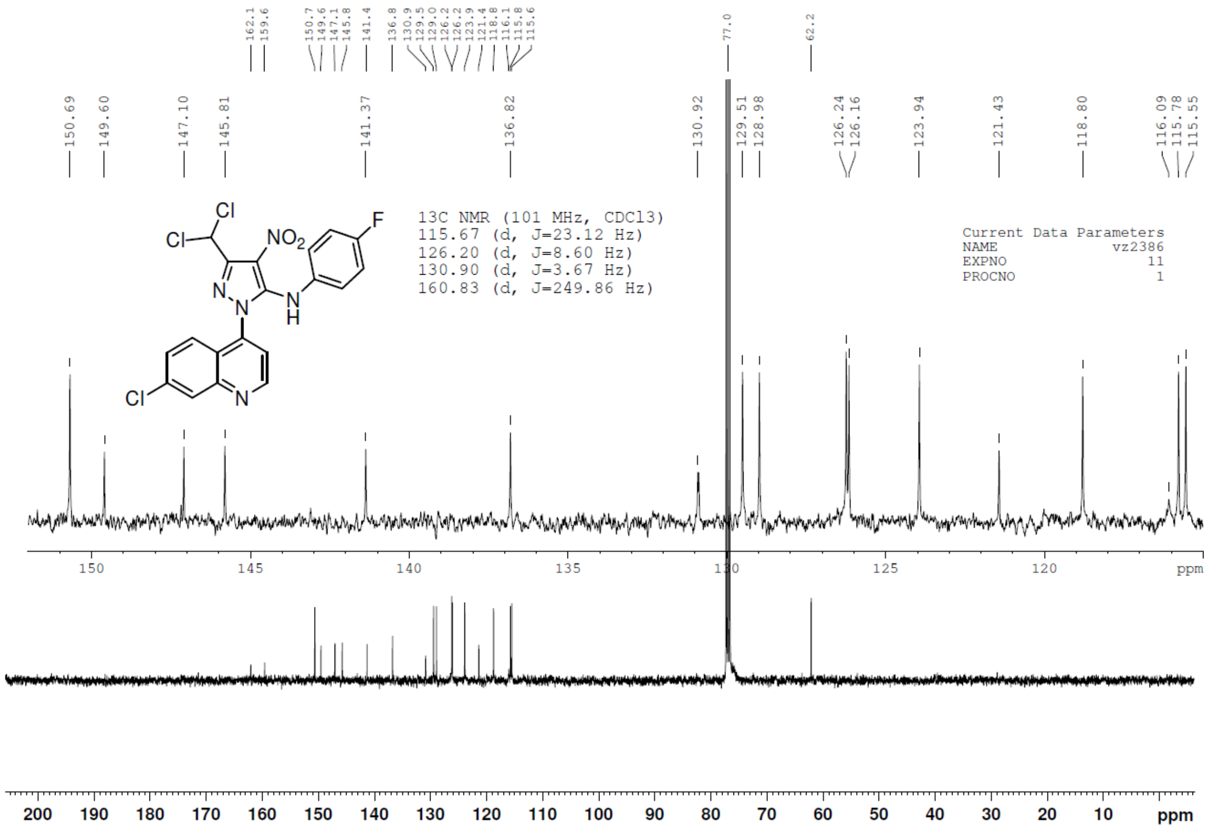

Figure S44. 100 MHz ^{13}C NMR spectrum in $CDCl_3$ for **5m**.

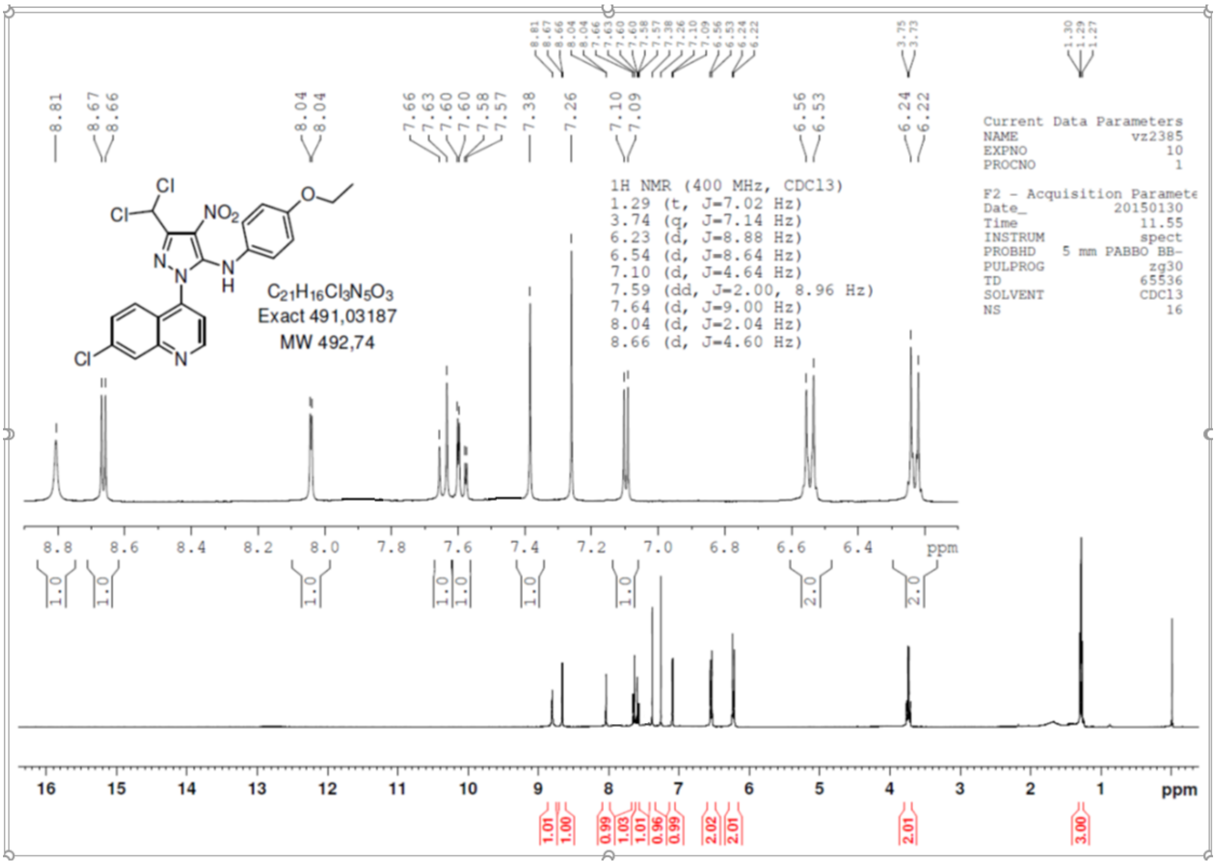

Figure S45. 400 MHz ^{1}H NMR spectrum in $CDCl_3$ for **5n**.

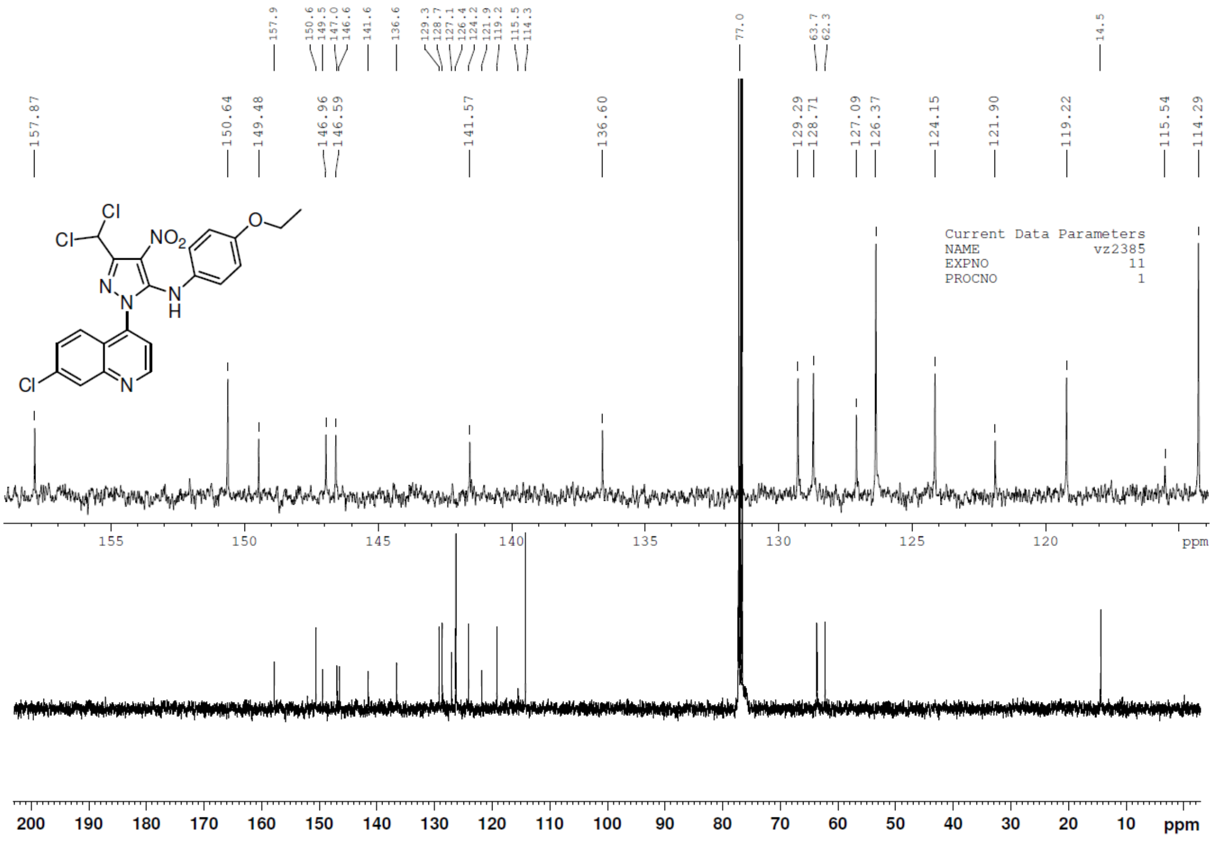

Figure S46. 100 MHz ^{13}C NMR spectrum in $CDCl_3$ for **5n**.

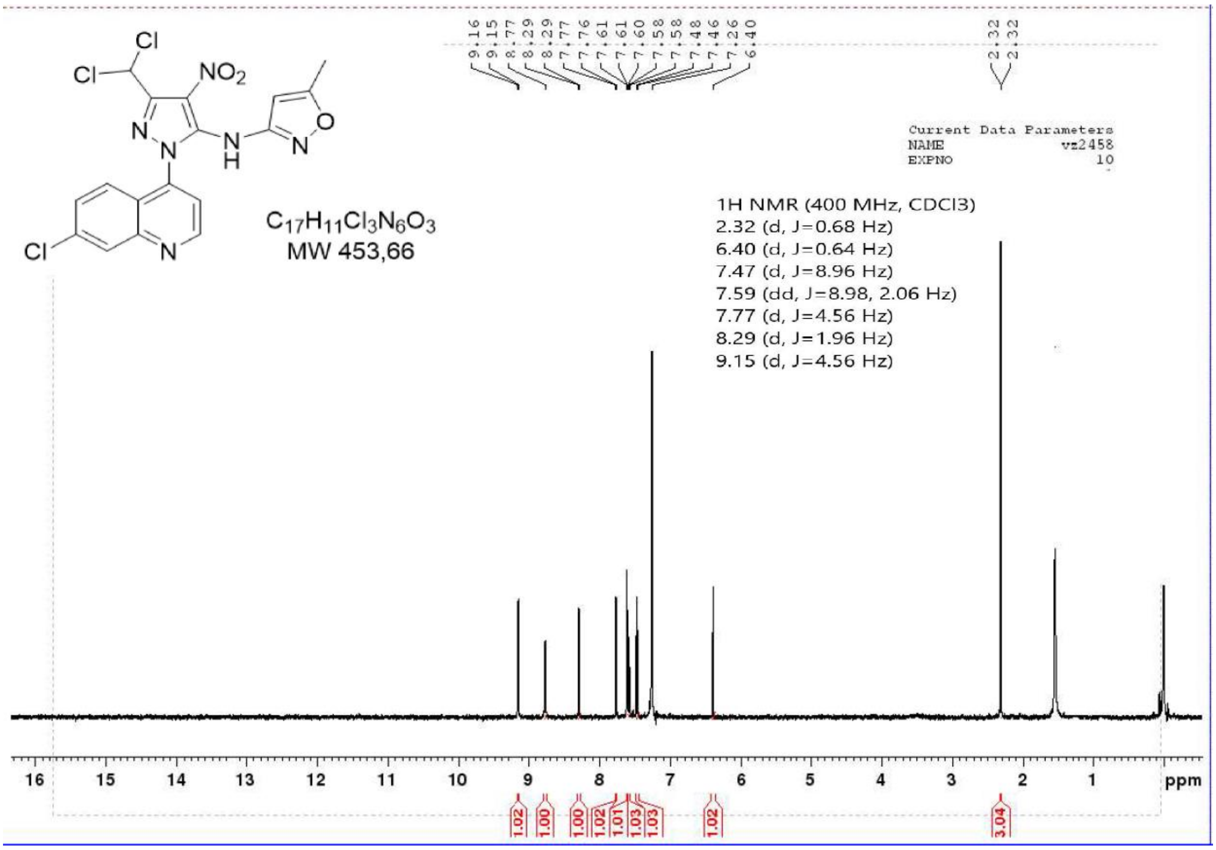

Figure S47. 400 MHz ^{1}H NMR spectrum in $CDCl_3$ for **5o**.

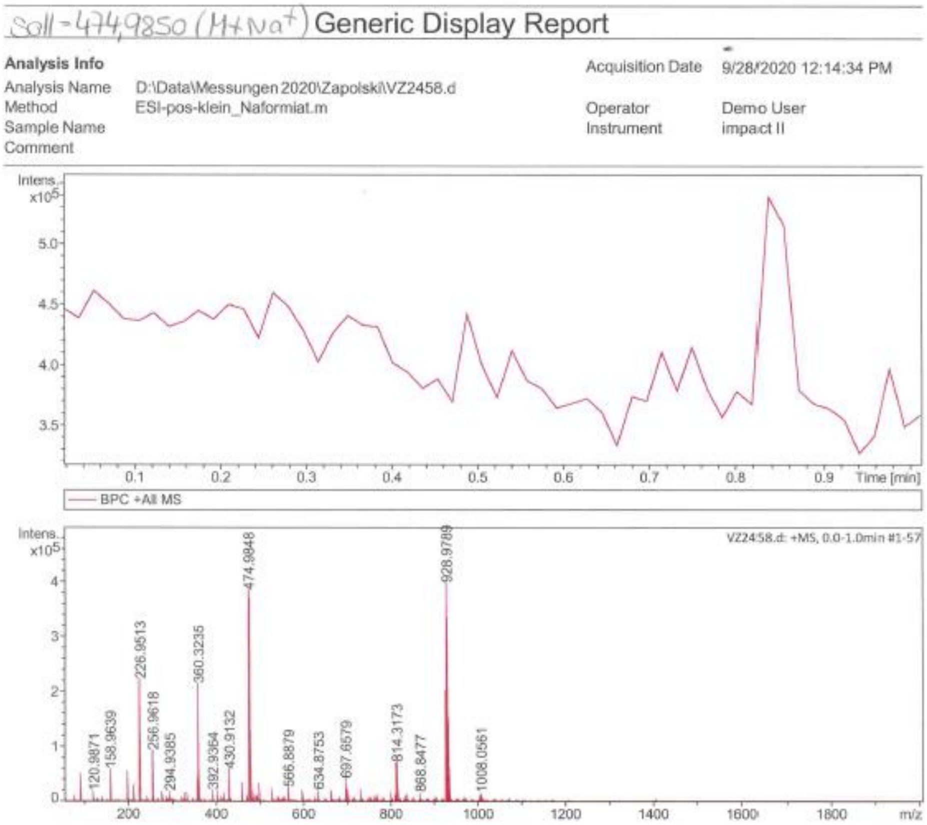

Figure S48. HRMS spectrum for *N*-(1-(7-chloroquinolin-4-yl)-3-(dichloromethyl)-4-nitro-1*H*-pyrazol-5-yl)-5-methylisoxazol-3-amine (**5o**).

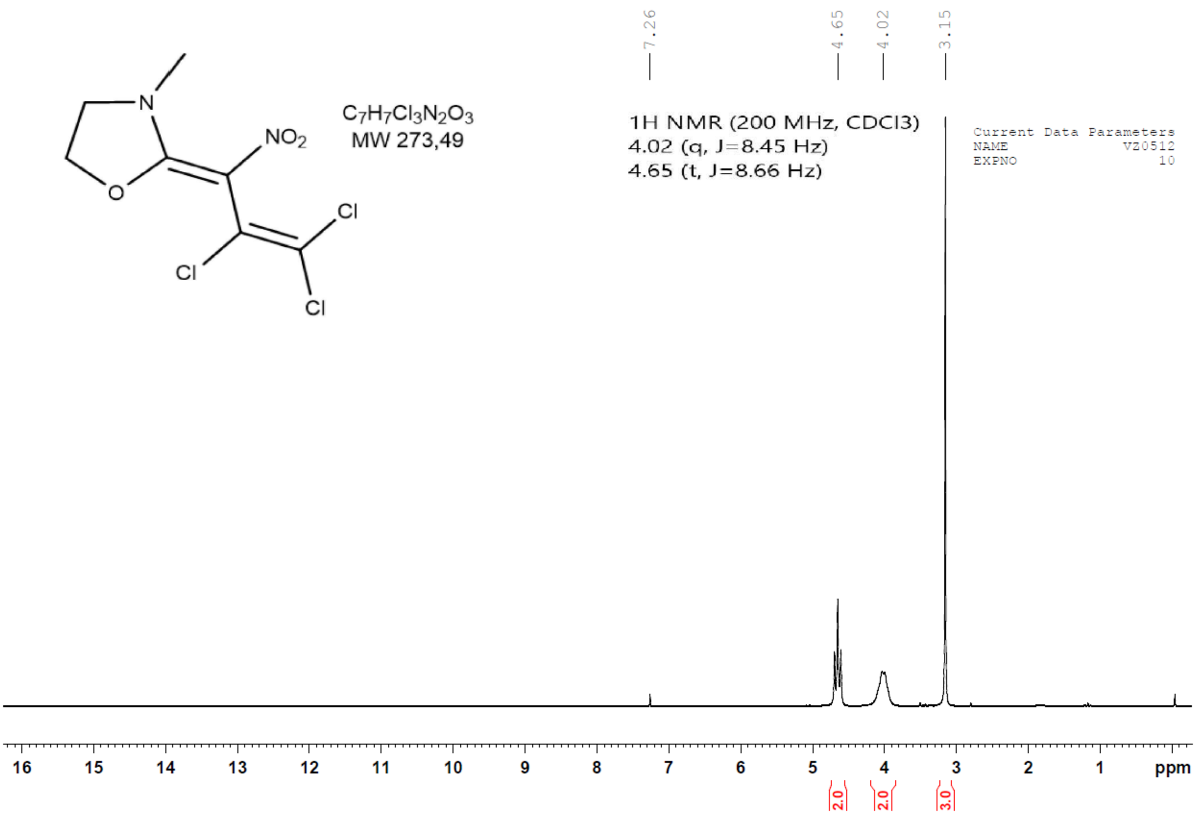

Figure S49. 200 MHz ^{1}H NMR spectrum in $CDCl_3$ for **6**.

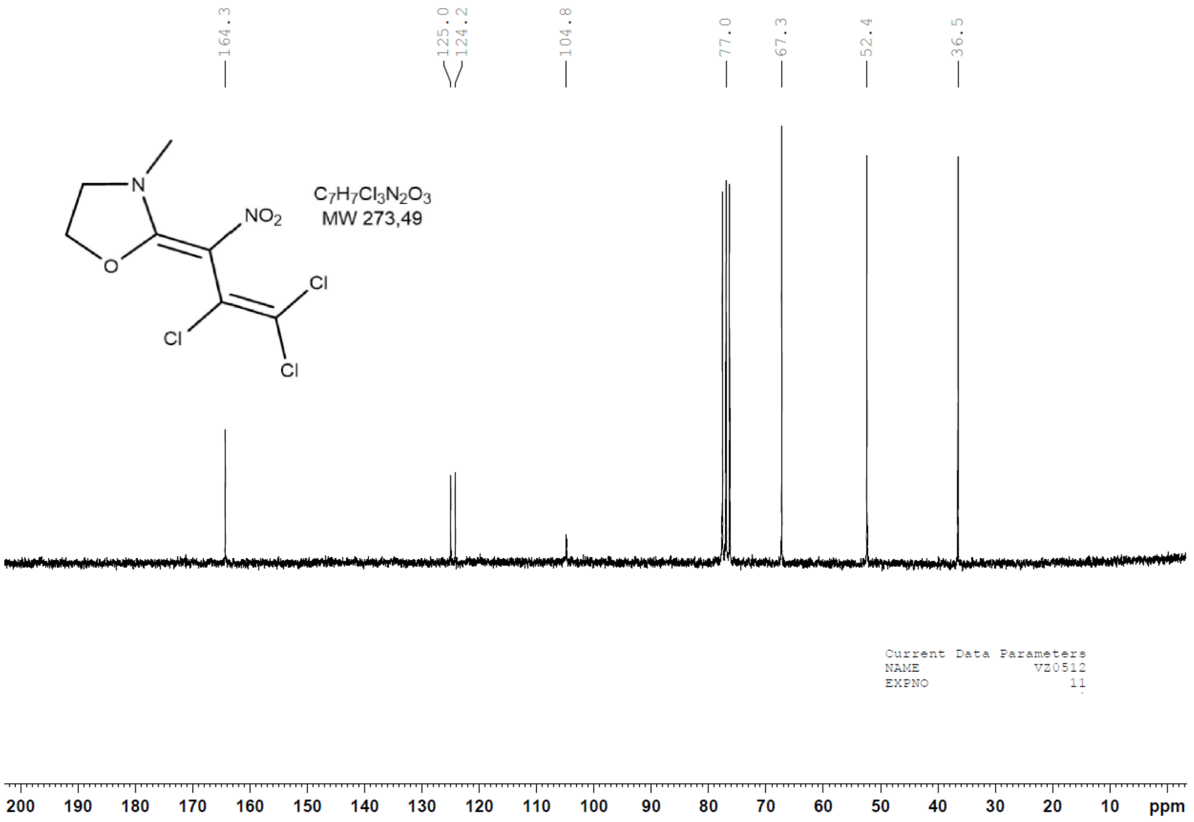

Figure S50. 50 MHz ^{13}C NMR spectrum in $CDCl_3$ for **6**.

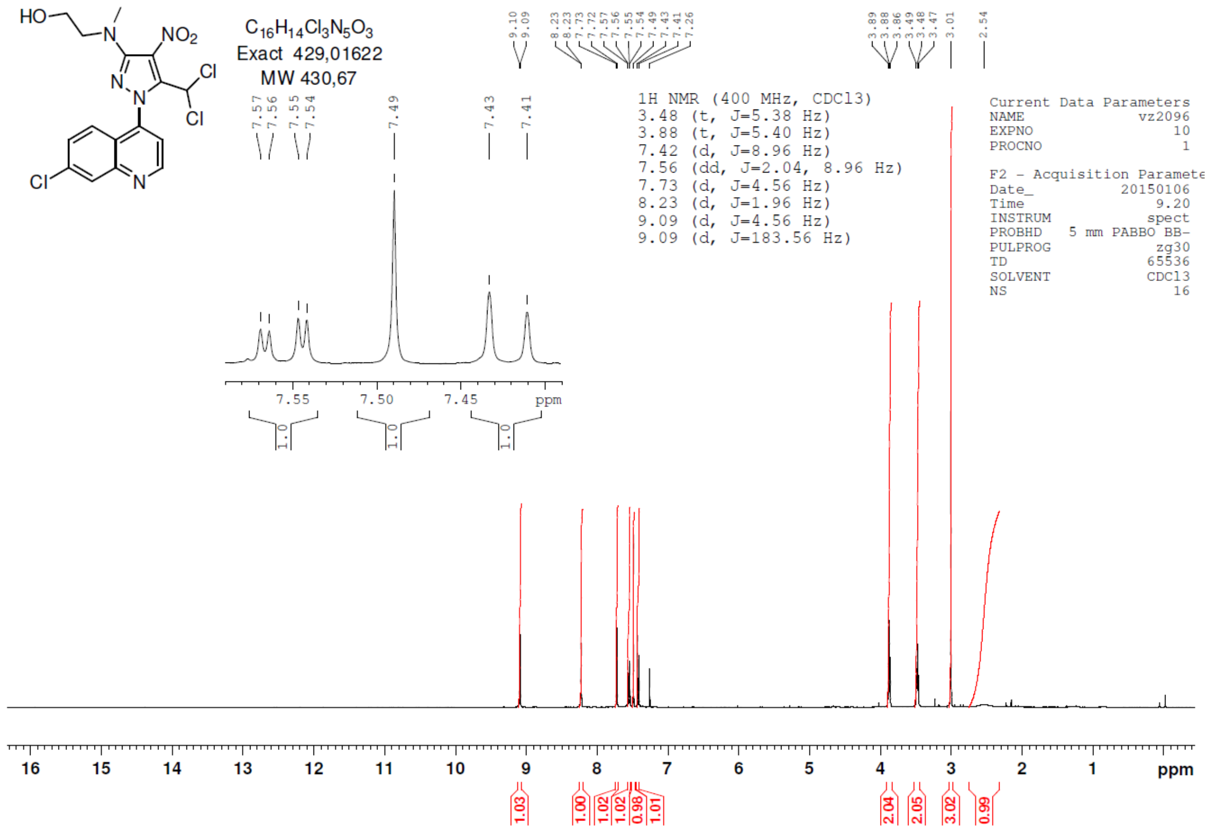

Figure S51. 400 MHz ^{1}H NMR spectrum in $CDCl_3$ for **7**.

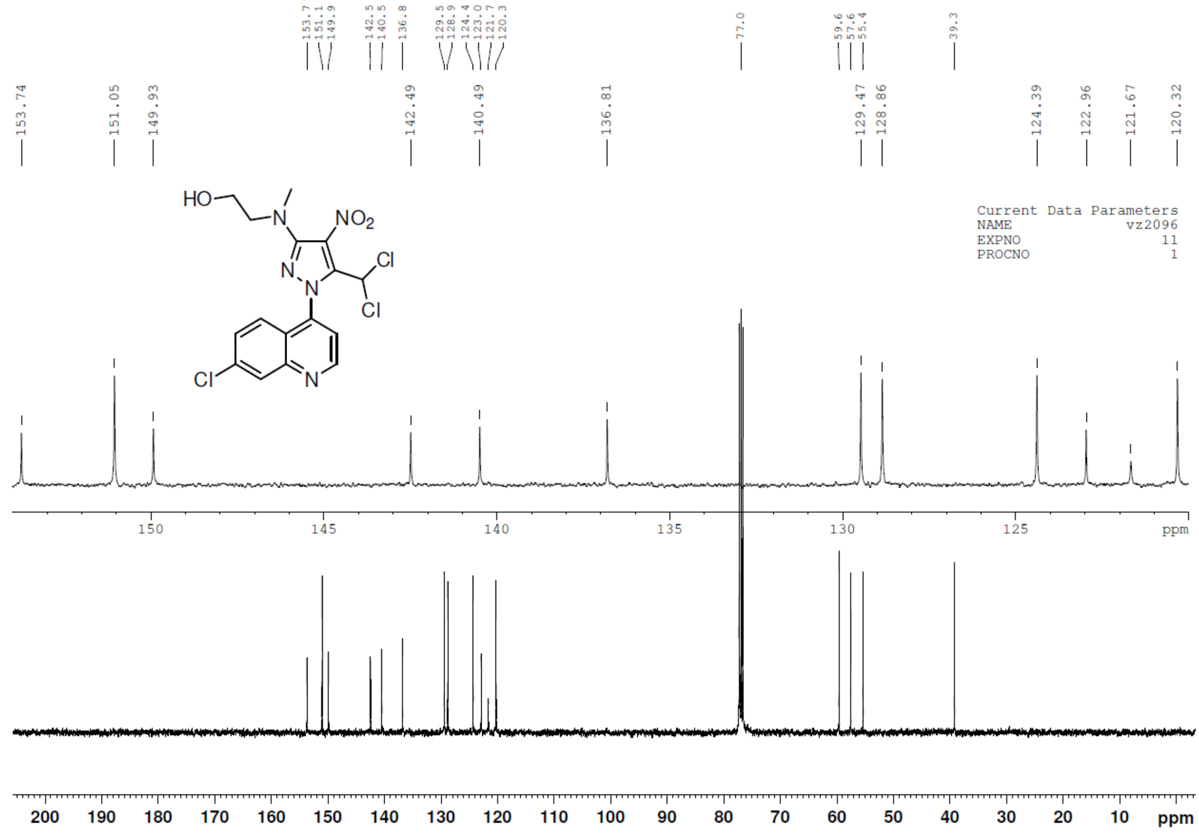

Figure S52. 100 MHz ^{13}C NMR spectrum in $CDCl_3$ for **7**.

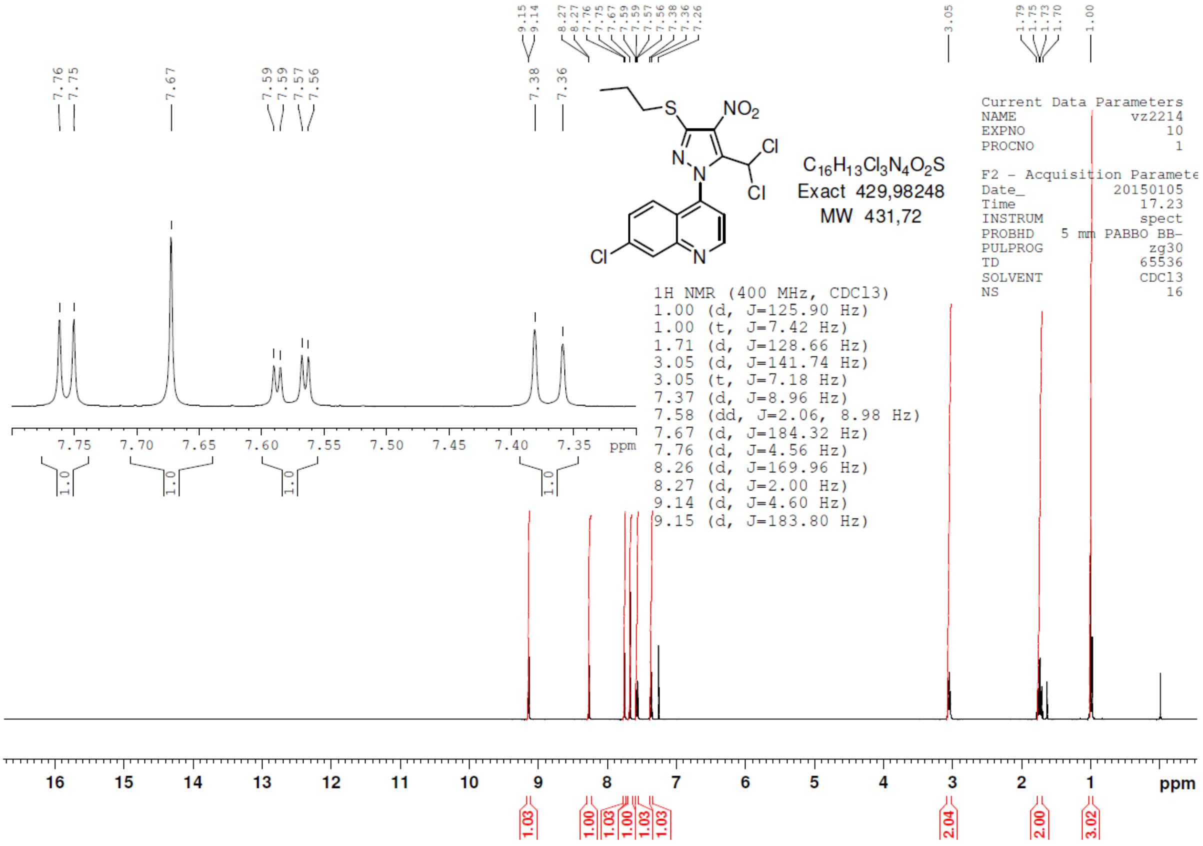

Figure S53. 400 MHz ^{1}H NMR spectrum in $CDCl_3$ for **9a**.

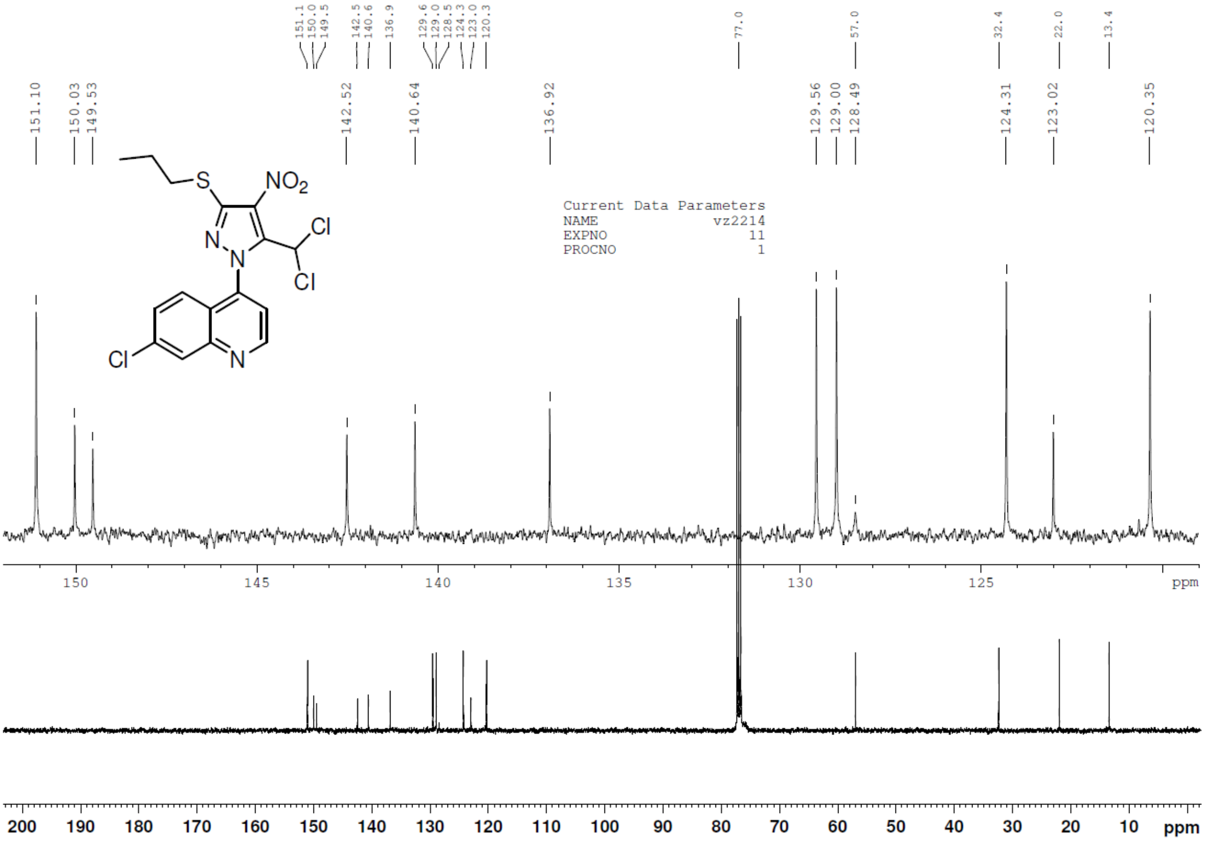

Figure S54. 100 MHz ^{13}C NMR spectrum in $CDCl_3$ for **9a**.

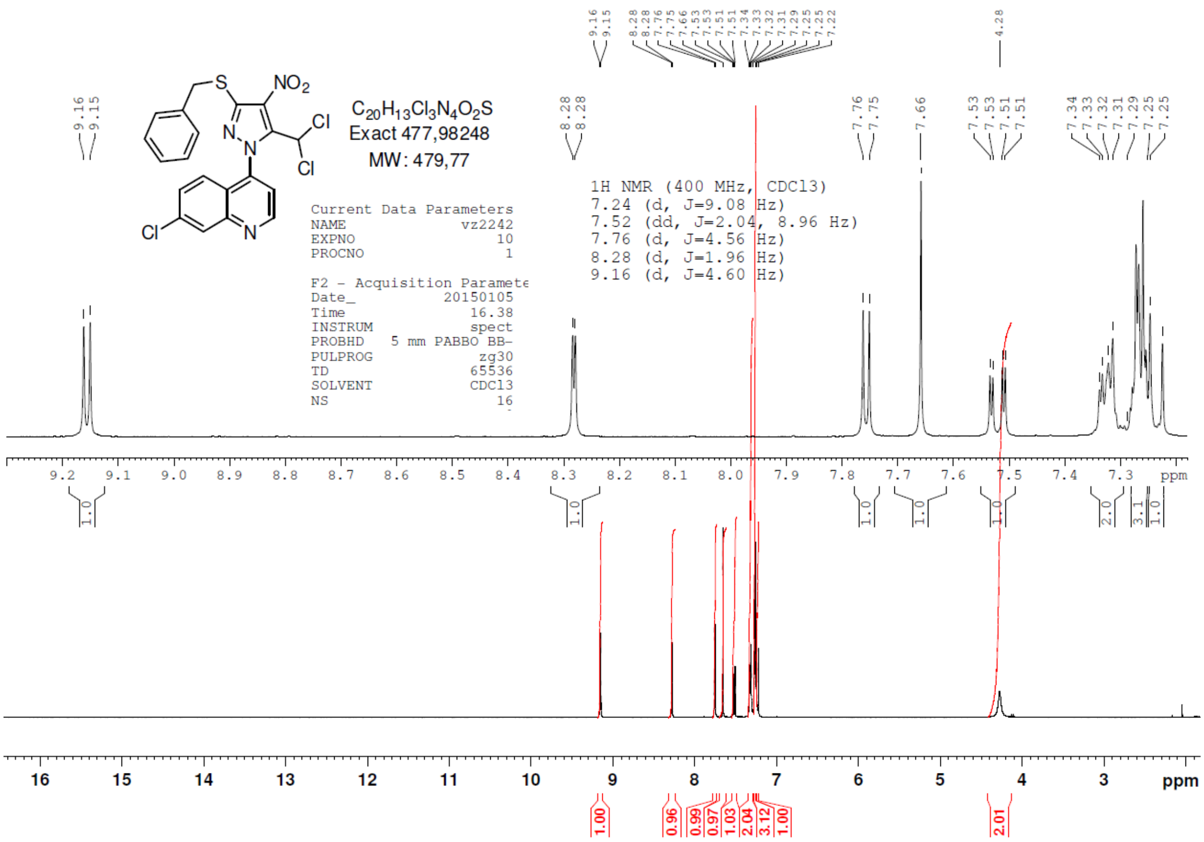

Figure S55. 400 MHz ^{1}H NMR spectrum in $CDCl_3$ for **9b**.

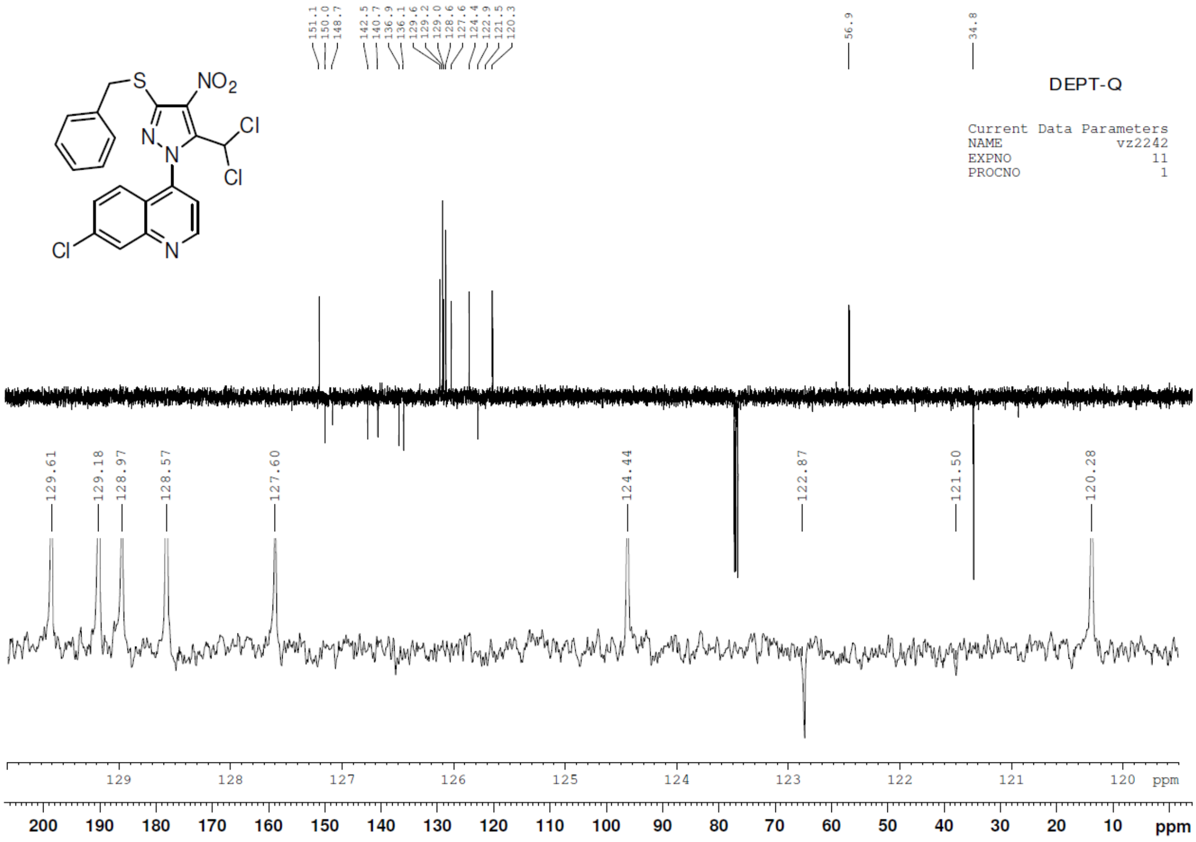

Figure S56. 100 MHz 100 MHz ^{13}C NMR DEPT-Q spectrum in $CDCl_3$ for **9b**.

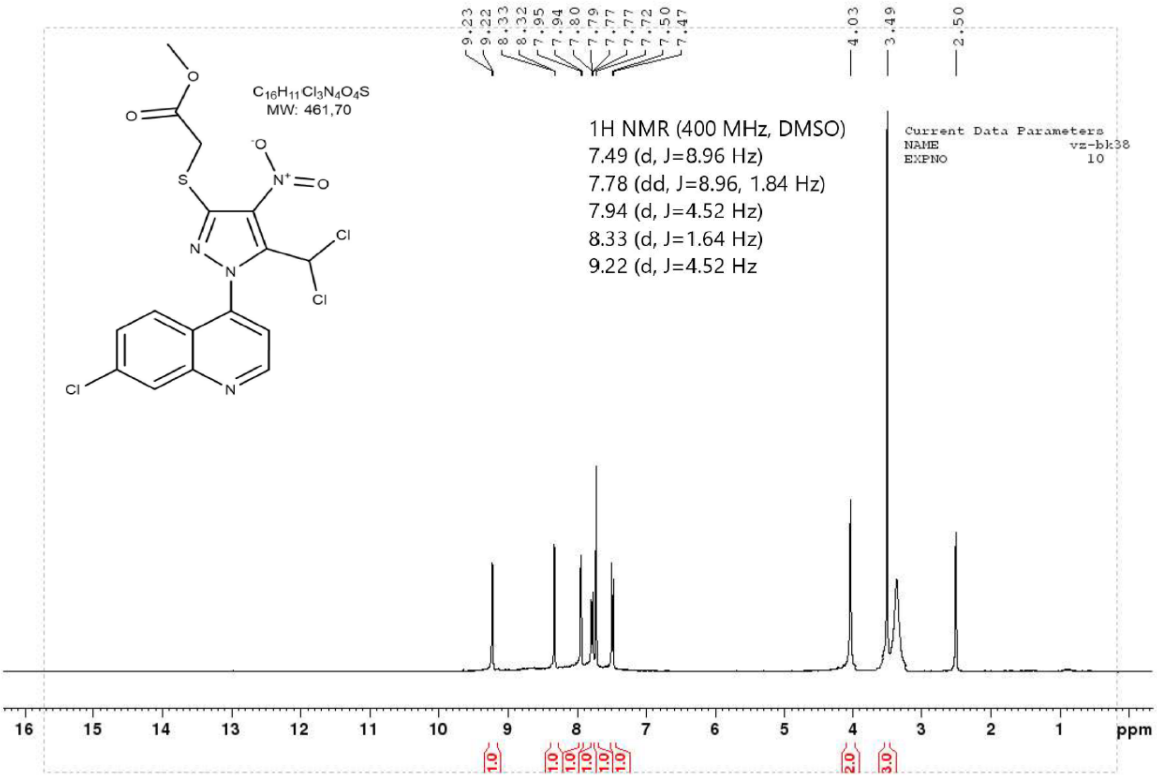

Figure S57. 400 MHz ^{1}H NMR spectrum in DMSO-d_6 for **9c**.

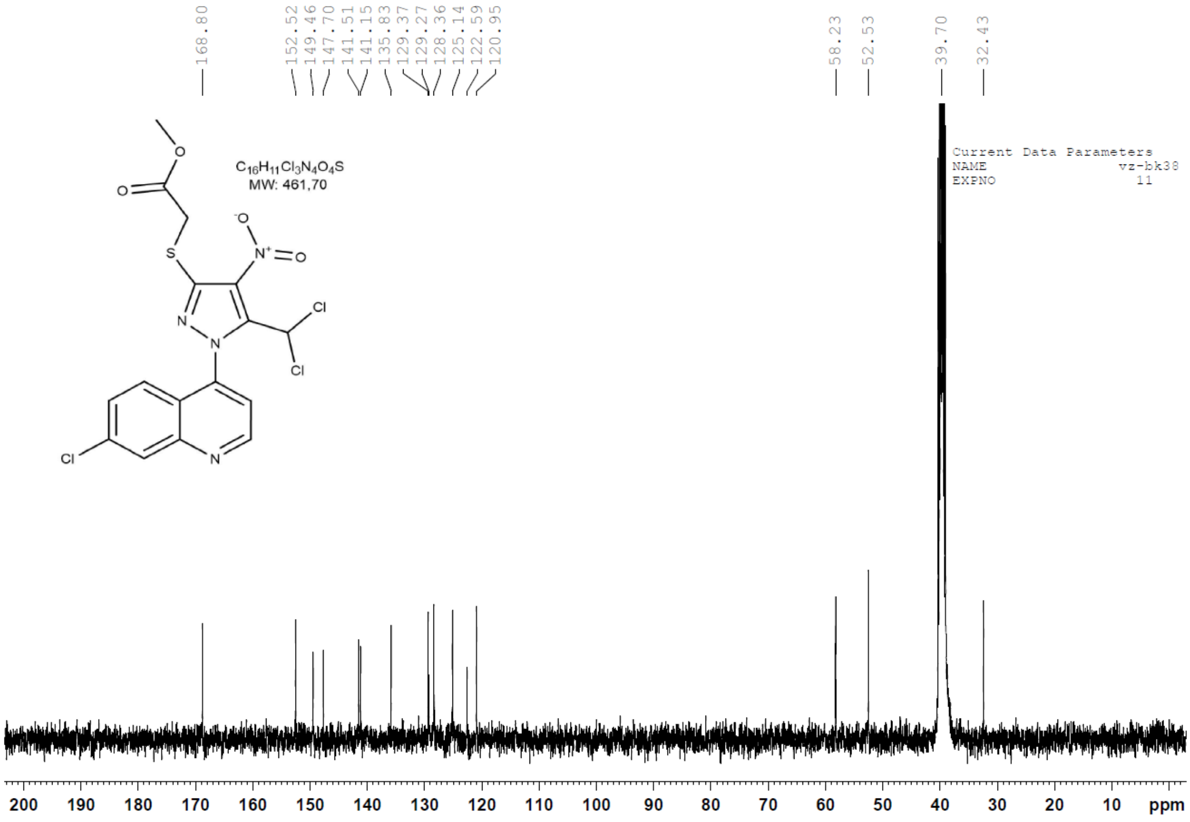

Figure S58. 100 MHz ^{13}C NMR spectrum in DMSO-d_6 for **9c**.

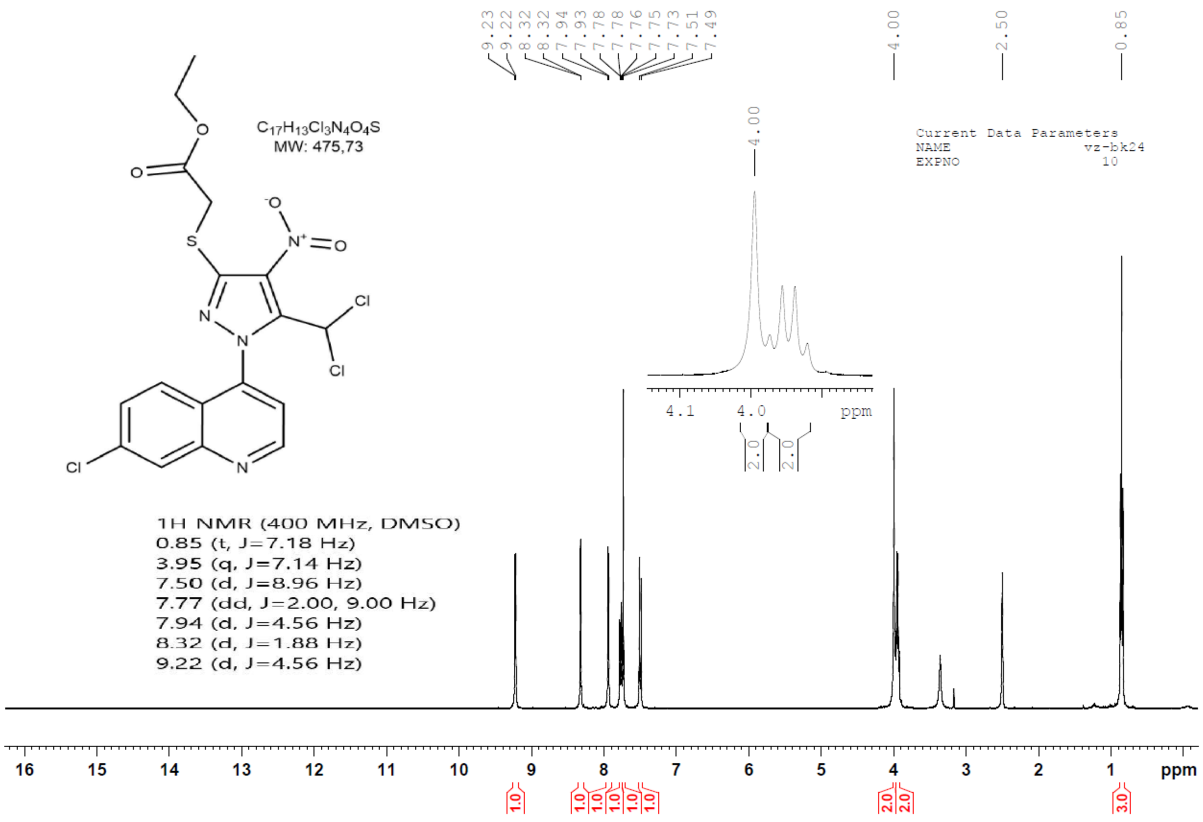

Figure S59. 400 MHz ^{1}H NMR spectrum in DMSO-d_6 for **9d**.

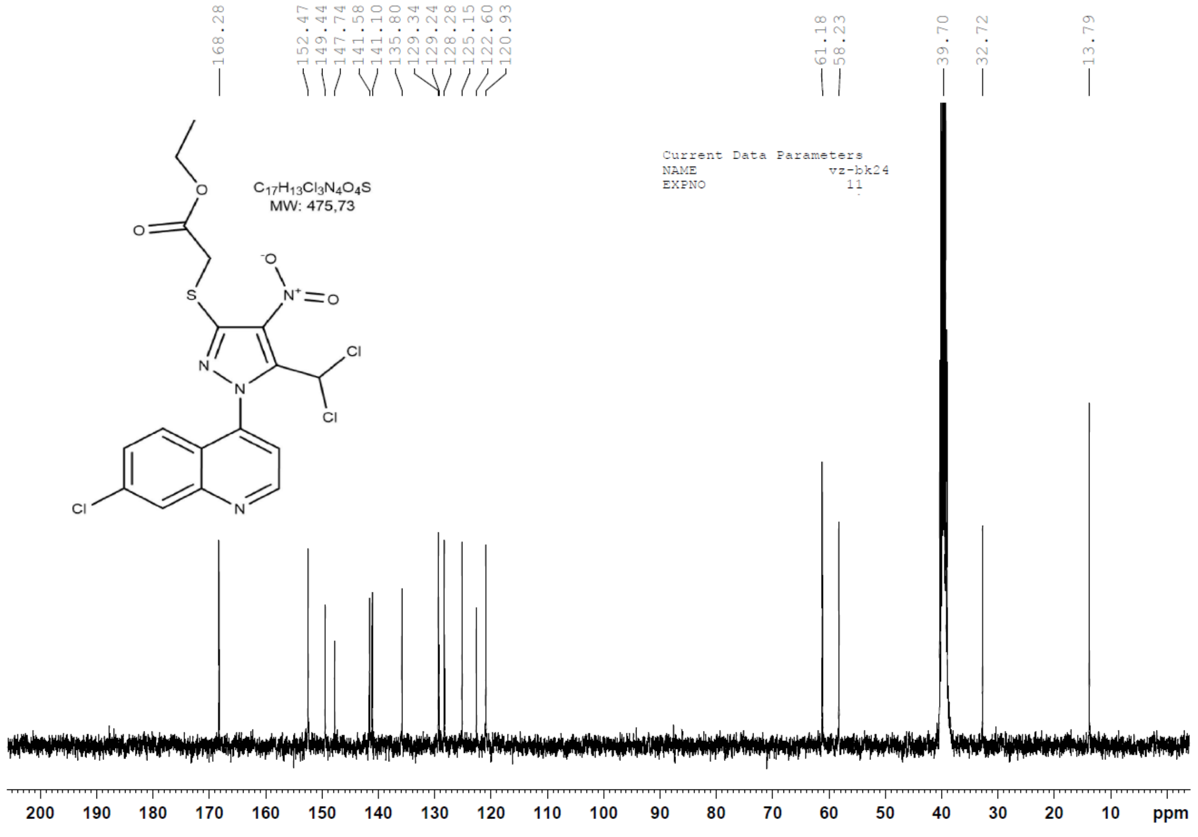

Figure S60. 100 MHz ^{13}C NMR spectrum in DMSO-d_6 for **9d**.

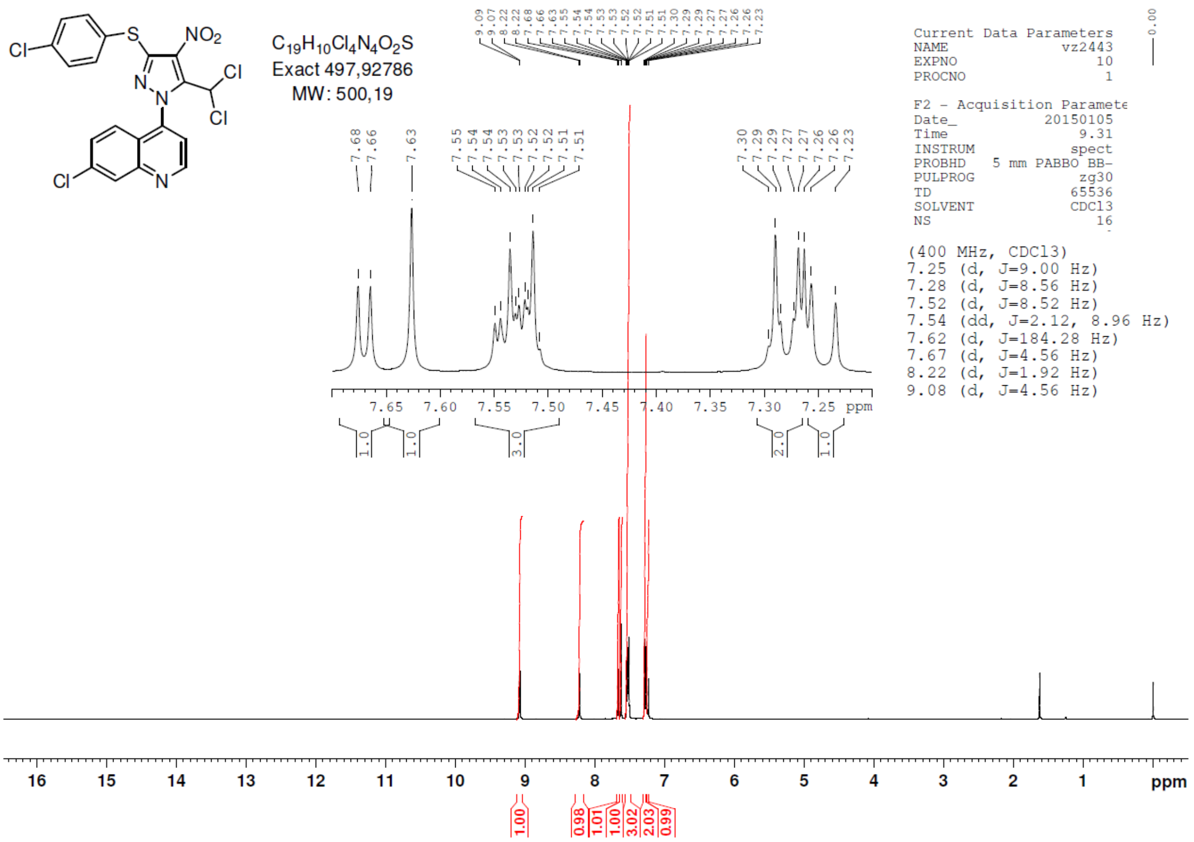

Figure S61. 400 MHz ^{1}H NMR spectrum in $CDCl_3$ for **9e**.

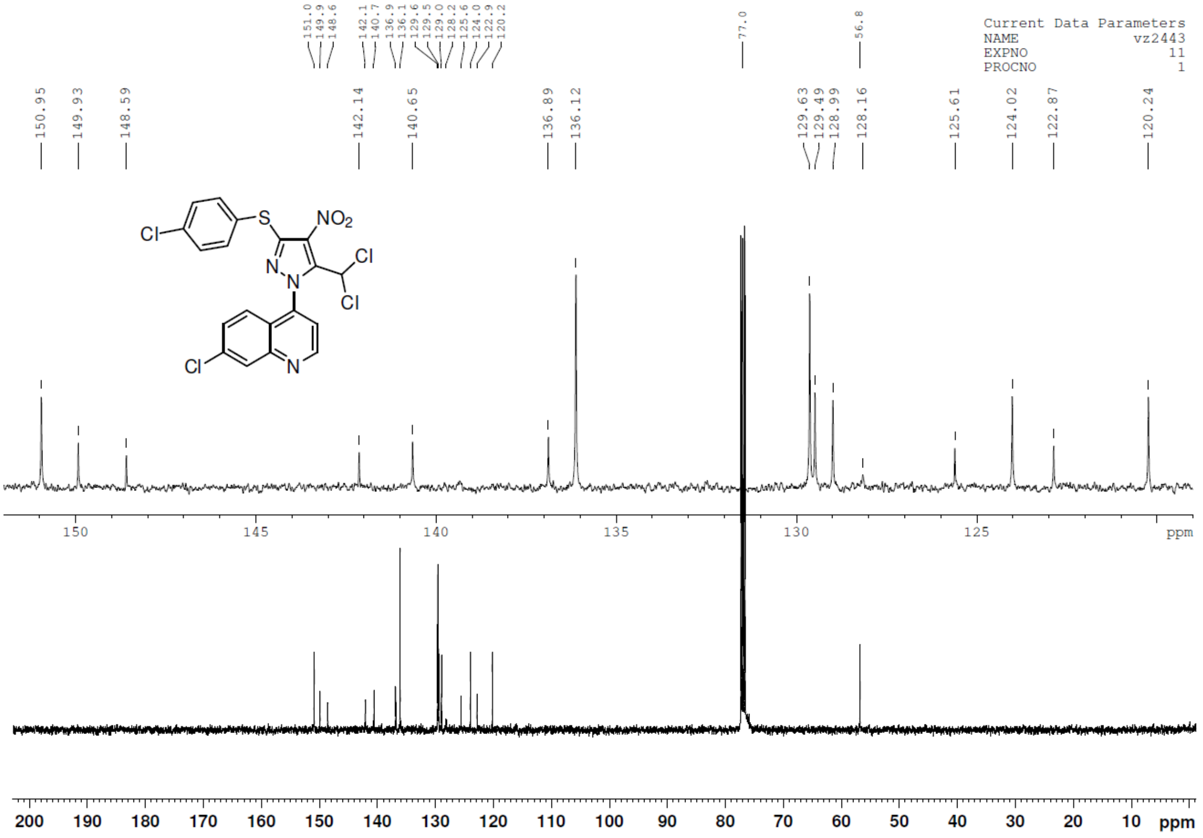

Figure S62. 100 MHz ^{13}C NMR spectrum in $CDCl_3$ for **9e**.

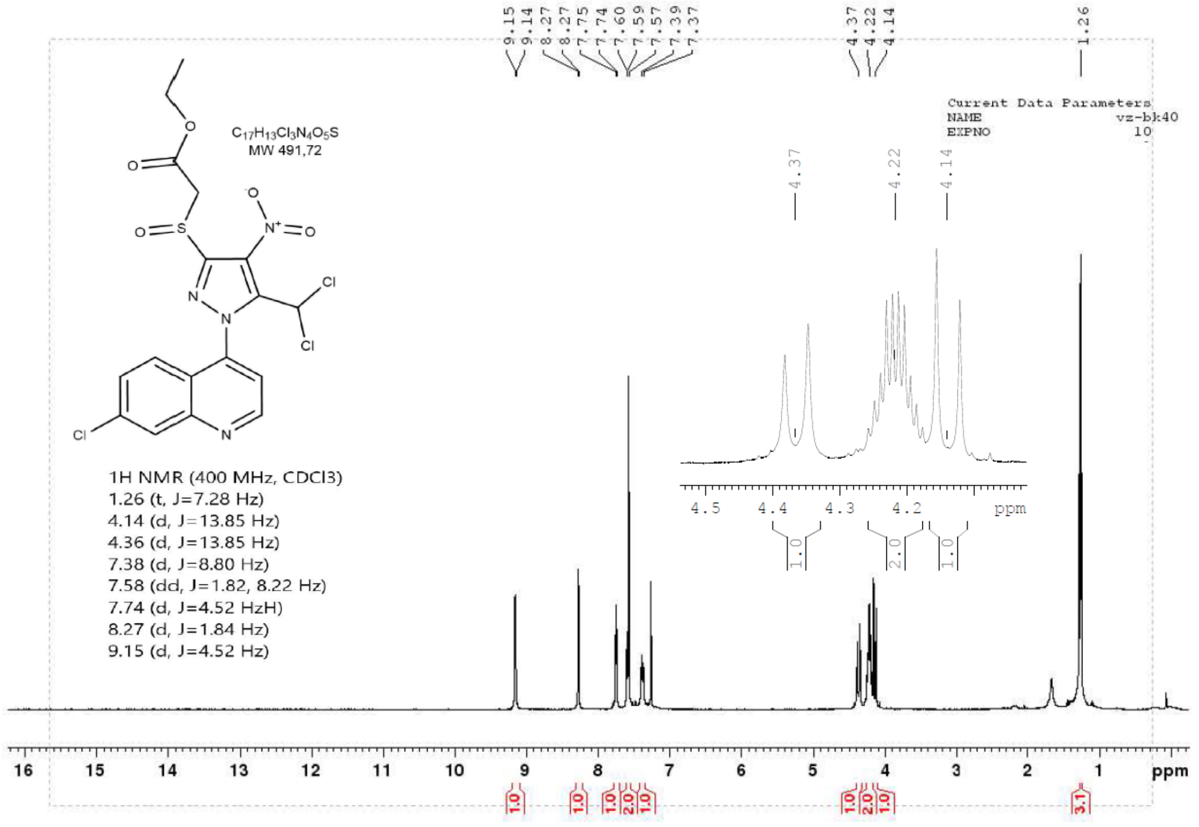

Figure S63. 400 MHz ^{1}H NMR spectrum in $CDCl_3$ for **10d**.

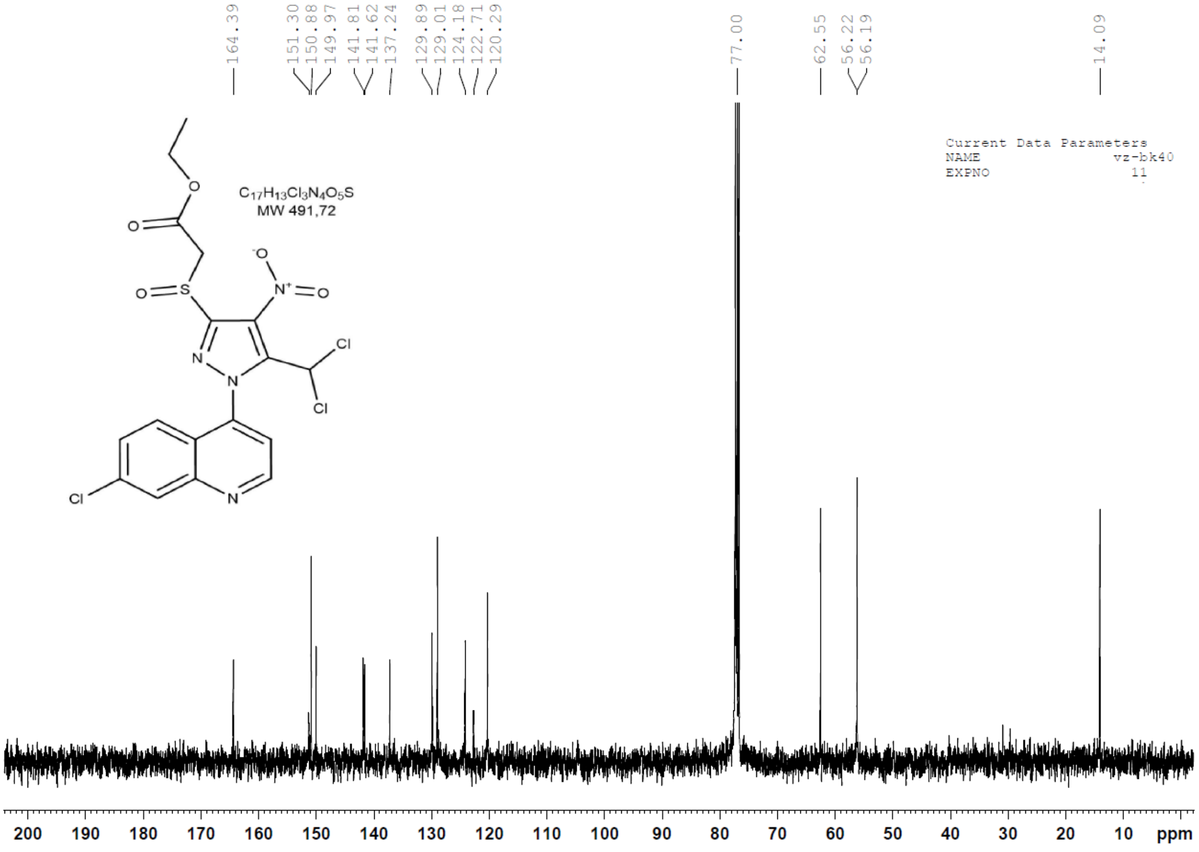

Figure S64. 100 MHz ^{13}C NMR spectrum in $CDCl_3$ for **10d**.

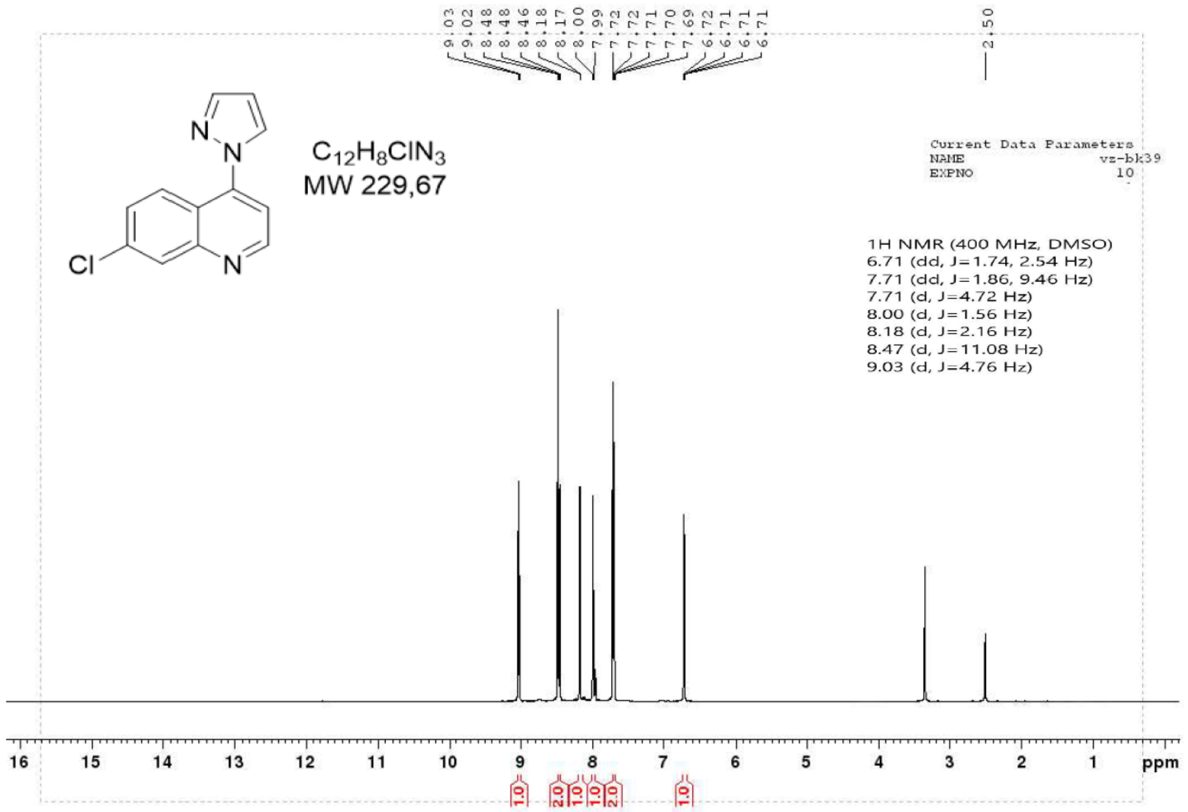

Figure S65. 400 MHz ^{1}H NMR spectrum in DMSO-d_6 for **11**.

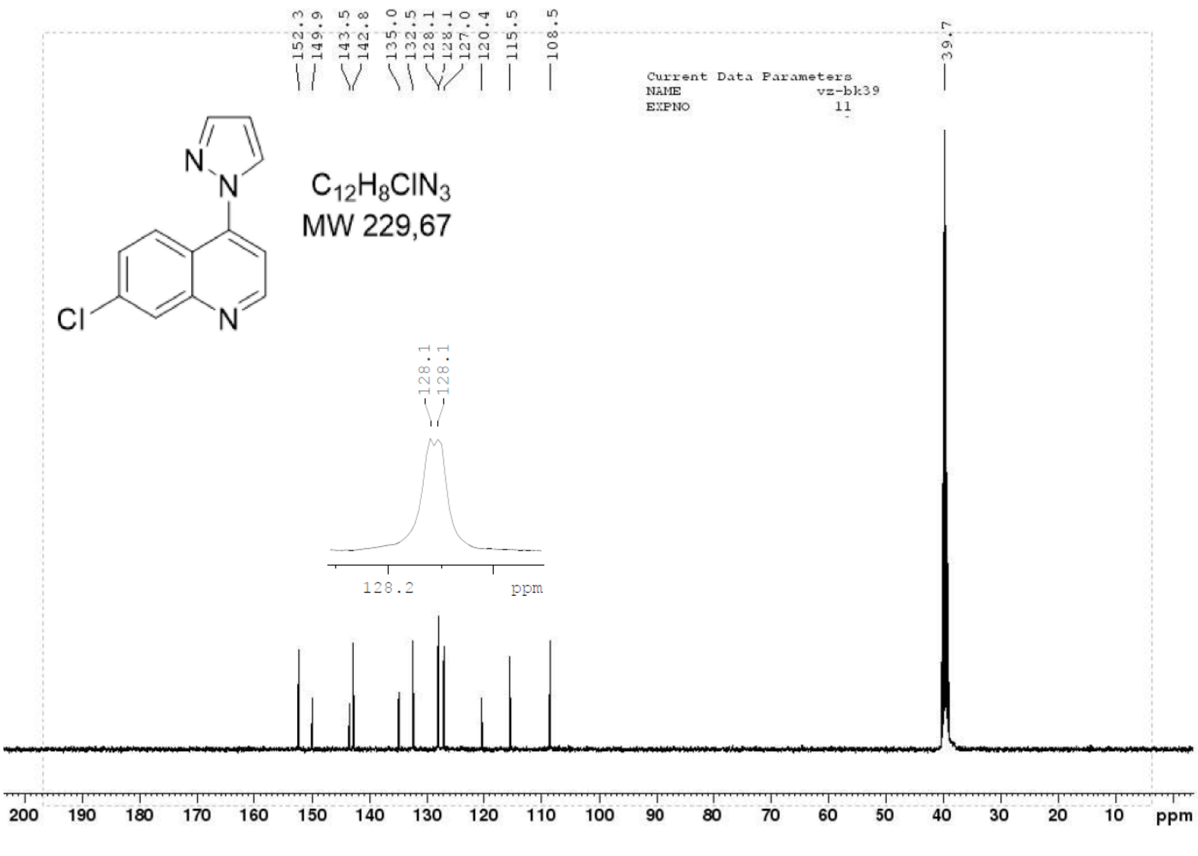

Figure S66. 100 MHz ^{13}C NMR spectrum in DMSO-d_6 for **11**.

5. Experimental procedures of biological assays

5.1 Antibacterial activity

Staphylococcus aureus SH1000 was used as representative Gram-positive bacterial strain and *Escherichia coli K12* and the respective ΔTolC deletion mutant as representative Gram-negative strains. The *E. coli* ΔTolC deletion mutant lacks the channel protein TolC of the AcrAB – TolC efflux pump, so that usually the intracellular concentration of small molecules is increased. *S. aureus* is cultivated in TSB (tryptic soy broth) medium, the *E. coli* strains in LB (lysogeny broth) medium.

The medium is inoculated from glycerol stocks of the bacteria or from colonies from an agar plate. Bacteria are cultivated with shaking at 37 °C over night. On the next day the bacterial suspension from the overnight culture is diluted with fresh medium to OD_{600} = 0.1. The optical density at 600 nm (OD_{600}) indicates the turbidity of the solution, i.e. bacterial growth. Aliquots of 90 µL of the bacterial suspension are given to each well of a half-area 96-well microplate (transparent, sterile). Alternatively, 384 well microplates are used with 60 µL bacterial suspension.

To each well 0.9 µL (or 0.6 µL) of the compound test solution is added preferably using the semi-automatic 96 channel pipettor CyBio® Selma (Analytic Jena GmbH, Jena, Germany). These plates are incubated without shaking at 37 °C. Growth of the bacteria is followed via determination of OD_{600} with the Epoch™2 microplate spectrophotometer (BioTek, Friedrichshall, Germany) at 0 h, 2 h, (4 h), and 24 h. Usually, the activity of test compounds is evaluated with a single, rather high concentration of 100 µM in the assay resulting from the 1:100 dilution of the 10 mM stock solution in DMSO. If growth is significantly inhibited, the influence of the compound concentration on growth inhibition is studied and the IC_{50} value (concentration leading to 50% growth inhibition) is determined using curve fitting by nonlinear regression with the software GraphPad Prism 9.0.2.

5.2 Cytotoxicity

The murine fibroblast cell line L929 is used to evaluate the cytotoxicity of the test compounds via the alamarBlue™ cell viability assay (ThermoFisher Scientific). Cells are cultivated in cell culture medium DMEM, supplemented with 10% FBS (serum) and 1% glutamine, in a cell culture incubator at 37 °C with 10% CO_2 in the atmosphere. 3000 cells in 60 µL medium are seeded in each well of a half-area 96-well microplate, transparent, sterile, cell-culture treated. They are placed in the incubator and allowed to adhere overnight. On the next day 0.6 µL of the test compound solution is added. Cells and compounds are incubated for 72 h in a cell culture incubator at 37 °C with 10% CO_2 in the atmosphere. By this long incubation time also processes are targeted, which are involved in proliferation of the cells. After 72 h, 5 µL of the resazurin solution are added to each well and incubated for another 4 h before fluorescence intensity is determined at λ_{ex} = 540 nm and λ_{em} = 600 nm. Low fluorescence values indicate low viability of the cells, i.e. high cytotoxicity of the test compound.

5.3 Antiviral activity

Vero E6 (ATCC CRL-1586) cells were maintained in DMEM medium supplemented with 10% fetal calf serum (FCS) and 2 mM L-glutamine.

The incubation of cells and viruses was performed at 37 °C in a 5% CO_2 atmosphere. The SARS-CoV-2 strain used in this study is a Zagreb isolate (hCoV-19/Croatia/ZG-297-20/2020, GISAID database ID: EPI_ISL_451934). All work with infectious viruses was performed in a biosafety level 3 facility.

The activity of test compounds against SARS-CoV-2 infection was tested by seeding Vero E6 cells one day before infection at a density of 7 × 10^3 cells per well in a 384-well cell culture plate (Thermo Fisher Scientific) in 40 µL medium. On the day of

infection, 5 µL of compound solutions were added to the cells and incubated for 1 h. Thereafter, cells were infected with 5 µL SARS-CoV-2 at a multiplicity of infection (MOI) of 0.01 and incubated at 37 °C in a 5% CO_2 atmosphere for 72 h. Viability of cells was used as indicator of virus replication, because replication of the virus caused cytopathic effects, i.e. cell damage. Cell viability was determined 72 h post infection with the CellTiter-Glo Luminescent cell viability assay (Promega). Cell-Titer-Glo reagents were prepared according to the manufacturer's instructions and the reaction was initiated by addition of 50 µL reagent per well to cells. Plates were incubated in the dark for 10 min at room temperature prior to luminescence measurement. Luminescence was determined using a Synergy HTX Multi-Mode plate reader (BioTek, Friedrichshall, Germany).

5.4 Antimalarial activity

Cell culture of Plasmodium falciparum. *P. falciparum* 3D7 strain was cultured as described in [7]. Briefly, the strain was cultivated in RBCs (A+) in RPMI 1640 medium supplemented with 0.5% w/v Albumax, 9 mM glucose, 0.2 mM hypoxanthine, 2.1 mM L-glutamine, 25 mM Hepes, and 22 µg mL^{-1} gentamycin at 3.3% haematocrit and 37 °C in a gaseous mixture consisting of 3% O_2, 3% CO_2, and 94% N_2. *Plasmodium falciparum* parasites were synchronized with 5% (w/v) sorbitol [8]. Parasitaemia was counted on Giemsa-stained blood smears.

In vitro activity of a novel inhibitor class against Plasmodium falciparum 3D7. The half maximal effective concentration (EC_{50}) of antimalarial compounds against *P. falciparum* 3D7 parasites was calculated using the SYBR Green I-based fluorescence assay for parasite nucleic acid according to [9] with slight modifications (Table S1).

The assay was performed in a 96-well format with a final volume of 300 µL. First, the DMSO-diluted compounds were serially double diluted in complete medium (100 µL, <1% DMSO) in 96-well microplates. Synchronized ring-stage parasites were added to each well (100 µL, 0.15% parasitaemia, 2.5% final heamatocrit) and incubated for 68 h at 37 °C. Plates were then frozen at −80 °C for 24 h. After thawing the plates for at least 2 h at rt, 100 µL of 1 × SYBR Green (of 10,000 × concentrated stock solution) in lysis buffer (50 mM Tris-HCl, 5 mM EDTA, 0.16% v/v Triton X-100, pH 7.4) were added to each well and again incubated for 2 h at rt in the dark. The fluorescence was measured at λ_{ex} = 494 nm/λ_{em} = 530 nm using a Clariostar plate reader. For EC_{50} calculation, the percentage growth inhibition was plotted against the log drug concentration using the GraphPad Prism 9 software (San Diego, CA, USA).

The results are collected in Table 1.

Prediction of ^{13}C NMR shifts

Table S1 shows the predicted and found (400 MHz, $CDCl_3$) ^{13}C NMR shifts for the C1–C4 atoms of the pyrazole ring and the dichloromethyl group in 5-methylaminopyrazole **5a** and 3-dimethylaminopyrazole **5b**. The prediction of ^{13}C NMR shifts was performed with ACD/Labs Software (2017) of Advanced Chemistry Development, Inc., Toronto, Canada [10].

Table S1: Predicted and found (400 MHz) ^{13}C NMR shifts for pyrazoles **5a**,**b**.

ACD/C-predicted and found (400 MHz) ^{13}C shifts for pyrazoles **5a–b**		^{13}C chemical shift for C1 – C4 atoms in $CDCl_3$			
		C1	C2	C3	C4
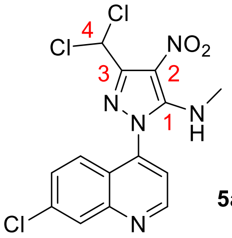 **5a**	found	149.4	114.7	142.4	62.3
	ACD/C	148.3	116.9	144.3	62.2
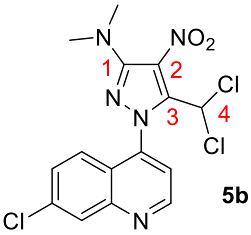 **5b**	found	154.0	121.6	136.7	57.7
	ACD/C	155.0	120.1	135.7	60.8

References

1. Ol'dekop, Yu. A.; Kaberdin, R. V. *J. Org. Chem. USSR (Engl. Transl.*), **1976**, *12*, 1986.
2. Potkin, V. I.; Zapol'skii, V. A.; Kaberdin, R. V. *Dokl. Natl. Acad. Sci. Belarus* **1996**, *40*, 68–71.
3. Vogt, E. Synthese und Charakterisierung 3,4-push-pull substituierter Thiophene für (opto)elektronische Materialien. Dissertation zur Erlangung des Doktorgrades der Naturwissenschaften. Clausthal, **2013**.
4. Zapol'skii, V. A.; Namyslo, J. C.; de Meijere, A.; Kaufmann, D. E. *Beilstein J. Org. Chem*. **2012**, *8*, 621–628. doi:10.3762/bjoc.8.69
5. Ol'dekop, Yu. A.; Kaberdin, R. V.; Potkin, V. I.; Shingel, I. A. *J. Org. Chem. USSR (Engl. Transl.),* **1979**, *15*, 39–41.
6. Singh, S. P.; Tarar, L. S.; Vaid, R. K.; Elguero, J.; Martinez, A. *J. Heterocyclic Chem*., **1989**, *26*, 733–738. doi:10.1002/jhet.5570260340
7. Rahbari, M.; Rahlfs, S.; Jortzik, E.; Bogeski, I.; Becker, K. *PLOS ONE* **2017**, *12* (4), e0174837. doi:10.1371/journal.pone.0174837
8. Lambros, C.; Vanderberg, J. P. *J. Parasitol.* **1979**, *65* (3), 418–420. https://pubmed.ncbi.nlm.nih.gov/383936/
9. Ekland, E. H.; Schneider, J.; Fidock, D. A. *FASEB J.* **2011**, *25* (10), 3583–3593. doi:10.1096/fj.11-187401
10. ACD/Labs Software of Advanced Chemistry Development, Inc., Toronto, Canada, 2017. https://www.acdlabs.com/
